# Air-Stable Tetrazene Radical Cation Salts: Structural Requirements and Oxidation Catalysts

**DOI:** 10.1021/jacs.5c15272

**Published:** 2025-12-15

**Authors:** Ayari Oshiro, Yusuke Sasano, Shu Saito, Yasuyuki Araki, Soichiro Sugiyama, Eunsang Kwon, Shinji Kajimoto, Yuse Kuriyama, Shohei Yoshinaga, Masaya Takahashi, Katsuhiko Sato, Naoki Shida, Yusuke Ishigaki, Mahito Atobe, Yoshiharu Iwabuchi

**Affiliations:** † Graduate School of Pharmaceutical Sciences, 13101 Tohoku University, 6-3 Aoba, Aramaki, Aoba-ku, Sendai 980-8578, Japan; ‡ Institute of Multidisciplinary Research for Advanced Materials (IMRAM), 13101 Tohoku University, 2-1-1 Katahira, Aoba-ku, Sendai 980-8577, Japan; § Department of Chemistry, Faculty of Science, 12810 Hokkaido University, Sapporo 060-0810, Japan; ∥ Research and Analytical Center for Giant Molecules, Graduate School of Science, 13101 Tohoku University, 6-3 Aoba, Aramaki, Aoba-ku, Sendai 980-8578, Japan; ⊥ Graduate School of Science and Engineering, 13154 Yokohama National University, 79-5 Tokiwadai, Hodogaya-ku, Yokohama 240-8501, Japan; # Faculty of Pharmaceutical Science, Tohoku Medical and Pharmaceutical University, 4-4-1 Komatsushima, Aoba-ku, Sendai 981-8558, Japan; □ Department of Chemistry and Life Science, Yokohama National University, 79-5 Tokiwadai, Hodogaya-ku, Yokohama 240-8501, Japan; △ Institute of Advanced Sciences, Yokohama National University, 79-5 Tokiwadai, Hodogaya-ku, Yokohama 240-8501, Japan; ▲ PRESTO, Japan Science and Technology Agency (JST), 4-1-8 Honcho, Kawaguchi, Saitama 332-0012, Japan

## Abstract

In this study, stable tetrazene radical cation salts were synthesized and characterized for the first time. The radical cation derived from 1,2-di­(2-azaadamantan-2-yl)­diazene (DAD) was isolated as an air-stable solid, retaining its integrity for at least 120 days at ambient temperature (∼25 °C) and pressure. X-ray crystallography and electron spin-resonance spectroscopy revealed the delocalization of the unpaired electron over the tetrazene core and into the adamantane framework. DAD undergoes two well-separated, reversible redox processes and displays high catalytic activity for alcohol oxidation under mild conditions. Systematic structural modifications identified the key framework features governing the radical cation stability and catalytic performance.

## Graphical abstract

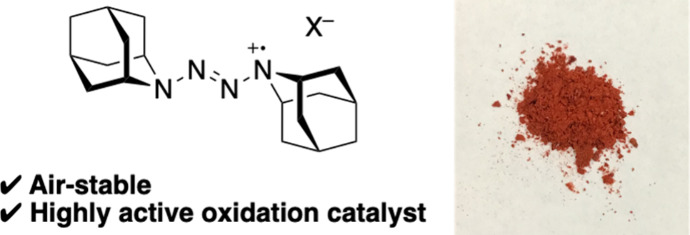

## Introduction

A radical is a chemical species containing at least one unpaired electron, which renders it highly reactive and short-lived. However, structural and electronic factors can confer sufficient stability for a radical to persist, and in certain cases, to be isolated as a stable species that persists for extended periods under ambient conditions.^1^ Stable radicals have attracted considerable interest due to their unique spin, magnetic, redox, and photophysical properties. Radical cation species are particularly intriguing because their counteranions can modulate these properties on demand.^2^ Because trivalent nitrogen atoms in organic compounds are prone to oxidation, various stable radical cation salts containing nitrogen atoms have been synthesized.^3,4^ However, the structural motifs that give rise to stable radicals that can persist for extended periods at room temperature and in air and can be isolated in pure form are limited. Most known examples are nitrogen radicals conjugated with an aromatic π-electron system such as triarylamine, phenothiazine, and dihydrophenazine (Figure 1a).

**Figure 1:**
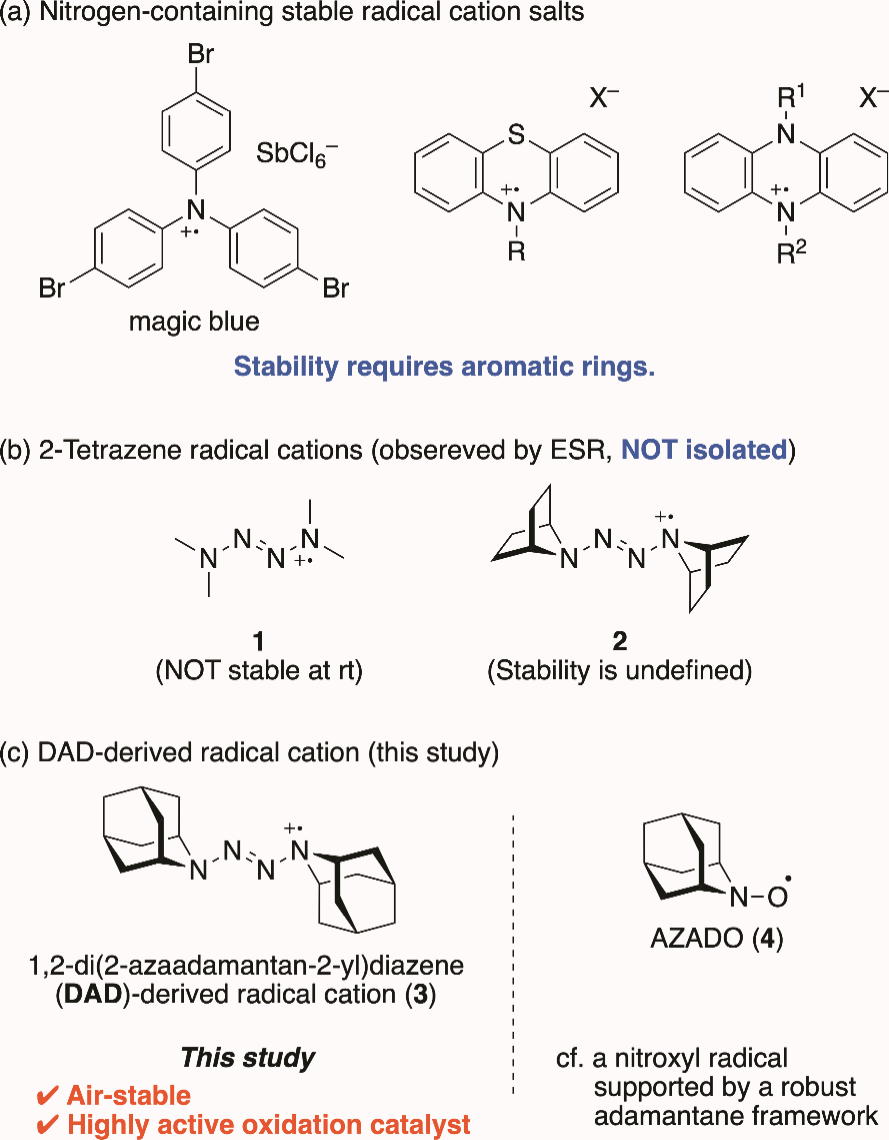
(a) Nitrogen-containing radical cation salts. (b) 2-Tetrazene radical cations. (c) DAD-derived radical cation (this study).

In this context, we report a new class of stable radical cation salts that lack aromatic rings, namely 2-tetrazene radical cation salts. 2-Tetrazene is a structural unit comprising four serially connected nitrogen atoms.^5^ Various tetrazene compounds have been synthesized as ligands for metal complexes and energetic materials. However, reports of radical cations produced by the one-electron oxidation of 2-tetrazene have been scarce. Using electron spin resonance (ESR) spectroscopy, **1** (Figure 1b), a radical cation derived from tetramethyltetrazene, and **2** (Figure 1b), a tetrazene radical cation with bicyclic structures, were analyzed by Tolles et al.^6^ and Nelsen et al.,^7^ respectively. However, **1** is unstable at room temperature, and stability of **2** has not been established in previous studies. Thus, no isolated or structurally characterized tetrazene radical cation salts have been reported to date.

We designed tetrazene radical cation **3** (Figure 1c) to exist in a stable form, with 1,2-di­(2-azaadamantan-2-yl)­diazene (DAD, **8**, Scheme 1) serving as its neutral precursor. This design was inspired by our previous work on the stable nitroxyl radical 2-azaadamantane *N*-oxyl (AZADO, **4**, Figure 1c) which possesses a highly robust adamantane skeleton.^8^ The rigid azaadamantane framework in AZADO supports its radical character, leading us to hypothesize that incorporation of both ends of a 2-tetrazene unit into azaadamantane skeletons will similarly support the corresponding radical cations. We also anticipated that **3** can serve as an oxidation catalyst analogous to AZADO. In this study, we successfully isolated air-stable tetrazene radical cation salts derived from DAD and demonstrated their high catalytic activities for alcohol oxidation.

**Scheme 1:**
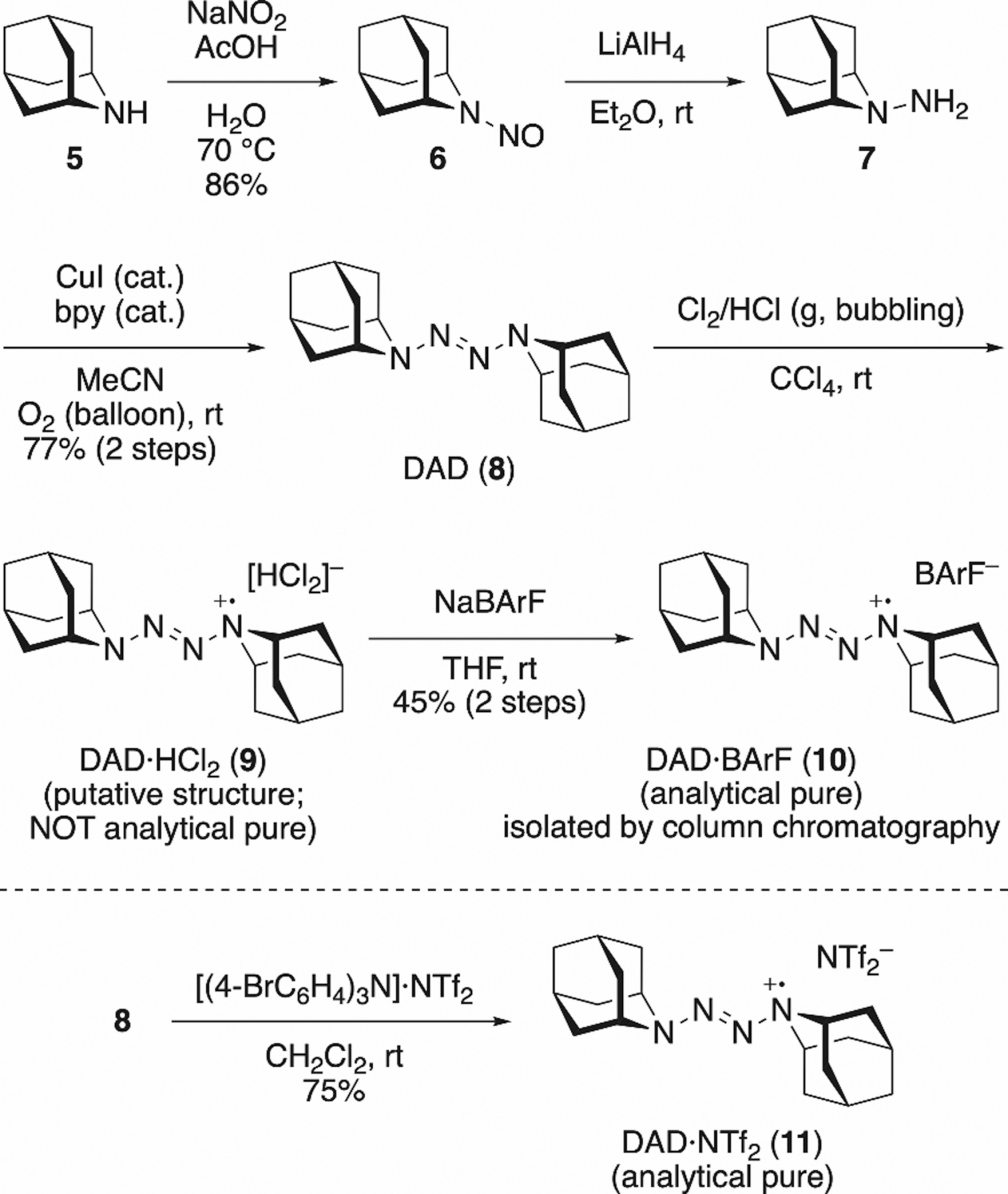
Synthesis of DAD and Its Radical Cation Salts

## Results and Discussion

### Synthesis and Characterization of Air-Stable Tetrazene Radical Cation Salts

This study commenced with the synthesis of DAD (**8**) and its corresponding radical cation salts from 2-azaadamantane (**5**) used as the precursor (Scheme 1).^8b^ Hydrazine **7**, obtained by nitrosation of **5** followed by reduction with LiAlH_4_, was oxidized according to the procedure described by Lacôte et al.^9^ to synthesize **8**.^10^ The oxidation of **8** by bubbling a chlorine/hydrogen chloride mixed gas afforded the DAD radical cation with hydrogen dichloride as the counteranion DAD·HCl_2_ (**9**; putative structure).^11^ Purification of **9** was challenging, and analytically pure samples could not be obtained. Therefore, we attempted to isolate the DAD radical cation by anion exchange with tetrakis­[3,5-bis­(trifluoromethyl)­phenyl]­borate (BArF). The resulting DAD radical cation with BArF as the counteranion DAD·BArF (**10**) was isolated as a red solid by silica gel column chromatography. Compound **10** displayed an absorption band at 460 nm (ε = 2.5 × 10^3^ M^–1^ cm^–1^) in its ultraviolet–visible (UV–Vis) spectrum (Figure 2a). Comparison of the absorbances of analytically pure **10** and **9** (of unknown purity) indicated that the purity of **9** was 87%. Notably, **10** was stable in the solid state for at least 120 d in ambient air at room temperature (∼25 °C) and atmospheric pressure, while **9**, which is water-soluble, showed little decomposition after being kept in an aqueous solution for over 7 d (Figure 2b). In addition, the oxidation of **8** using tris­(4-bromophenyl)­ammoniumyl bis­(trifluoromethanesulfonyl)­imide (NTf_2_^–^) as the oxidant afforded DAD·NTf_2_ (**11**), the corresponding tetrazene radical cation salt.

**Figure 2:**
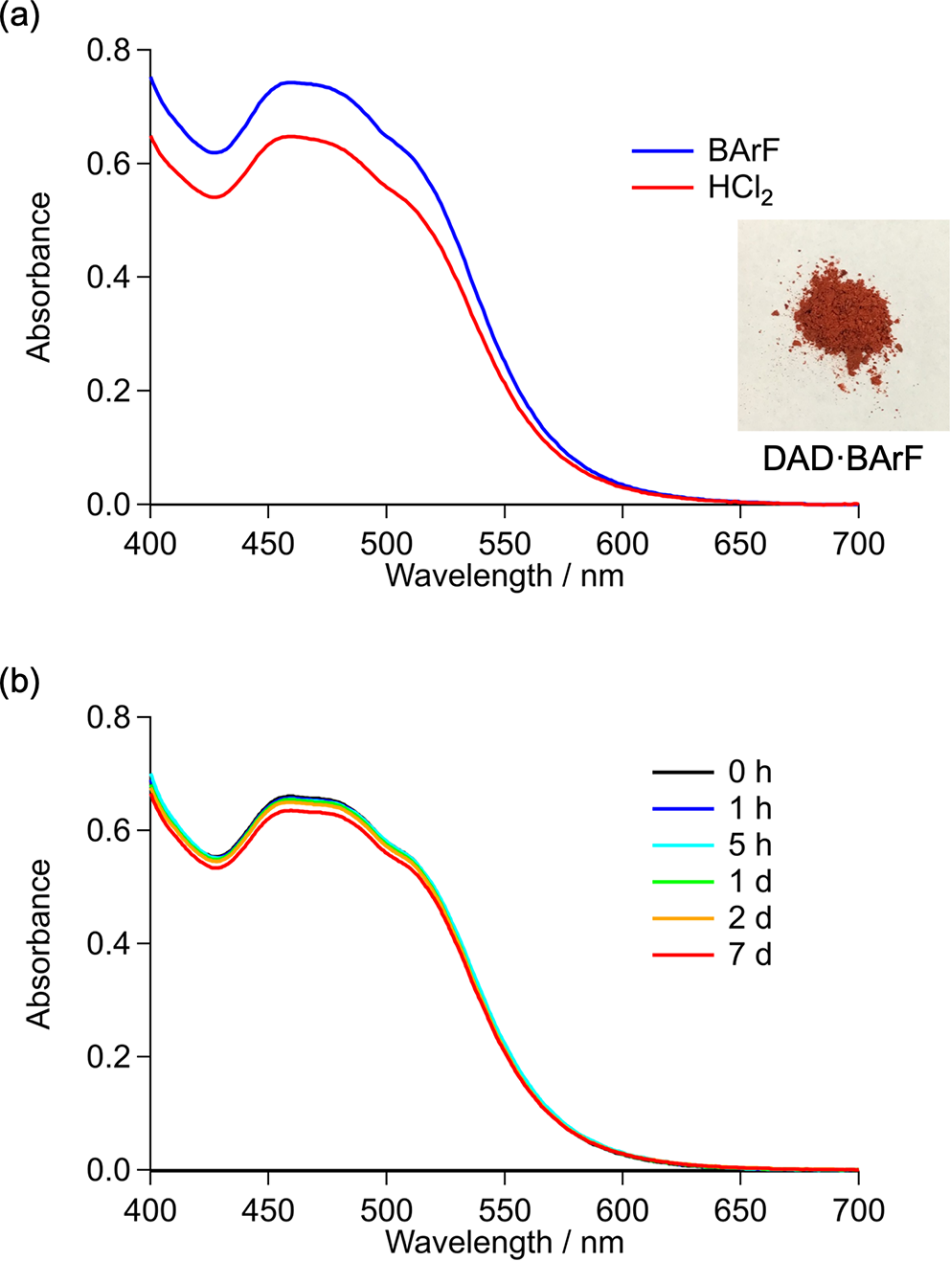
(a) UV–Vis spectra of DAD·BArF and DAD·HCl_2_ in 0.3 mM-MeCN, and photograph of solid DAD·BArF. (b) Time-dependent UV–Vis spectra of DAD·HCl_2_ in 0.3 mM aqueous solution (7 d).

With the isolated **10** in hand, its structure and spectroscopic properties were investigated. A single crystal suitable for X-ray crystallographic analysis was obtained via recrystallization from *n*-hexane/CHCl_3_. The crystal structure revealed tetrazene-derived cations and BArF anions in a 1:1 ratio, indicating that the DAD-derived component was a monovalent cation (Figure 3). The four nitrogen atoms in the DAD cation exhibited nearly identical N–N bond lengths of ∼ 1.31 Å, consistent with the delocalization of the unpaired electron over the tetrazene framework. This structural feature is further supported by density functional theory (DFT) calculations (see Supporting Information for details).

**Figure 3:**
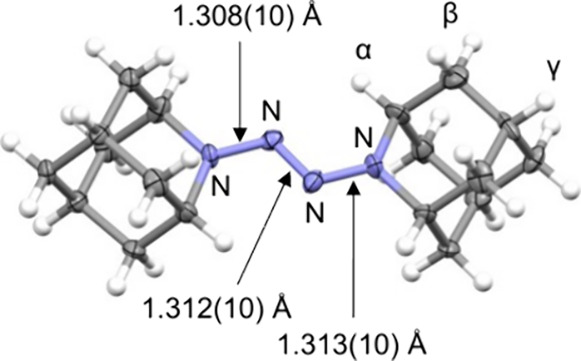
Crystal structure of DAD·BArF. The thermal ellipsoids are drawn at a probability level of 50% and counteranions are omitted for clarity.

To elucidate the radical nature of **10** in detail, its ESR spectrum was measured (Figure 4). The spectrum exhibited a broad line width exceeding the field modulation width (0.03 mT). ESR measurements at various concentrations revealed a proportional relationship between the concentration and signal intensity, indicating that intermolecular interactions between radicals do not contribute to the broad line width (Figure S6). Therefore, this broadening is attributed to the intrinsic nature of radical cations.

**Figure 4:**
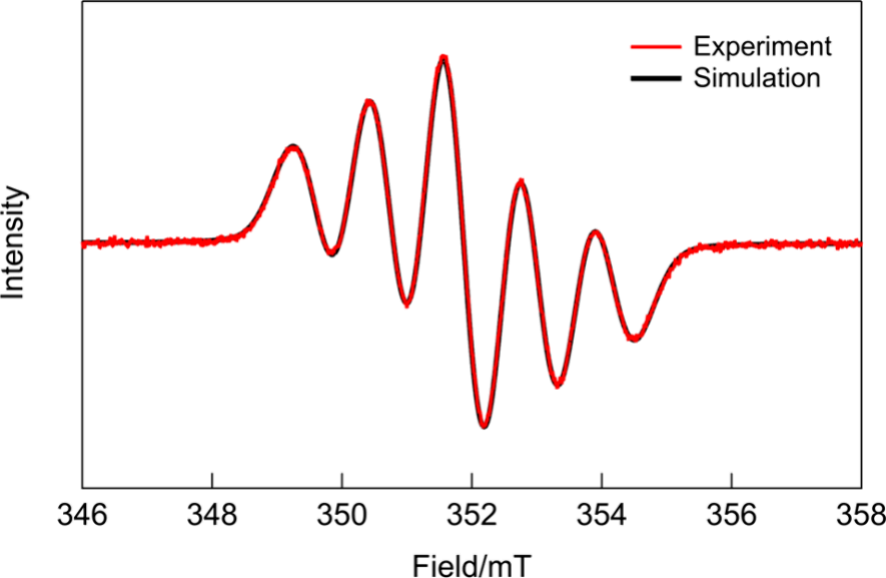
Experimental ESR spectrum of DAD·BArF in dichloromethane at room temperature and the ESR spectrum obtained by simulation.

These ESR features were rationalized using a spin-density analysis based on DFT calculations. In addition to the four nitrogen atoms, significant spin density was found on the β- and γ-hydrogen atoms of the adamantane skeleton (Figure 5; see also Figure S7), and at least six hydrogen atoms exhibited hyperfine coupling constants greater than 1.5 mT. A simulation of the ESR spectrum considering a spin system comprised by six hydrogen and four nitrogen atoms reproduced the experimental spectrum, yielding the hyperfine coupling constants shown in Table 1.

**Figure 5:**
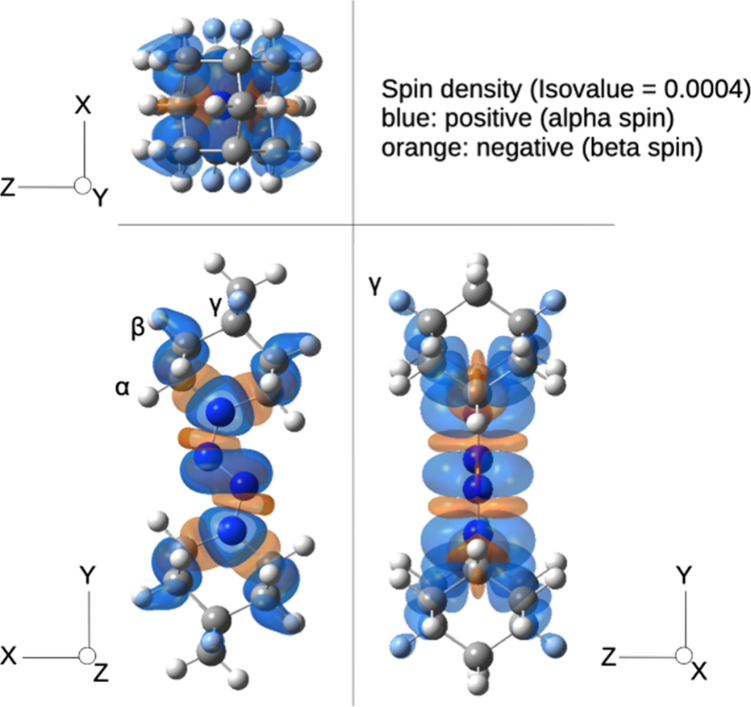
Calculated spin density distribution of the DAD-derived radical cation **3** (PCM-UB3LYP/EPR-III, isovalue = 0.0004).

**Table 1:** Hyperfine Coupling Constants Evaluated via ESR Simulation

atom	hyperfine coupling constant/gauss	number of nuclei
N	11.2	2
N	0.916	2
H	1.29	6

The ESR simulation results highlight two key points. (i) The radical cation likely possesses *C*_*2*_ symmetry with the origin at the midpoint of the central nitrogen atoms, as indicated by the even number of equivalent atoms contributing to hyperfine coupling, which is consistent with the X-ray crystallographic results. (ii) The electron spin of the DAD radical cations is delocalized over nearly the entire molecule, including the C­(sp^3^)–H bonds of the adamantane framework, demonstrating that the unpaired electron density extends beyond the tetrazene core.^12^

### Structure–Stability Relationships of Tetrazene Radical Cation Salts

As demonstrated above, DAD·BArF (**10**) can be isolated and remains stable in air at room temperature despite lacking aromatic rings. To identify the structural factors responsible for the stability of tetrazene radical cation salts, we synthesized a series of derivatives in which the cage framework of adamantane was systematically modified, and examined whether their radical cations could be isolated. Specifically, we designed two compounds shortened by one carbon atom relative to DAD (**8**)—namely, 1,2-di­(9-azanoradamantan-9-yl)­diazene (Nor-DAD, **12**, Figure 6), which retains the tricyclic framework, and 1,2-di­(9-azabicyclo[3.3.1]­nonan-9-yl)­diazene (DAND, **13**, Figure 6), which has a bicyclic framework. We also designed 1,2-di­(8-azabicyclo[3.2.1]­octan-8-yl)­diazene (DAOD, **14**, Figure 6), which is shortened by one methylene unit relative to **13**, and 1,2-di­(7-azabicyclo[2.2.1]­heptan-7-yl)­diazene (DAHD, **15**, Figure 6), which lacks an additional methylene unit relative to **14**. These tetrazenes were prepared from the corresponding secondary amines using the same procedure as that used for the preparation of **8** (see Supporting Information for details).

**Figure 6:**
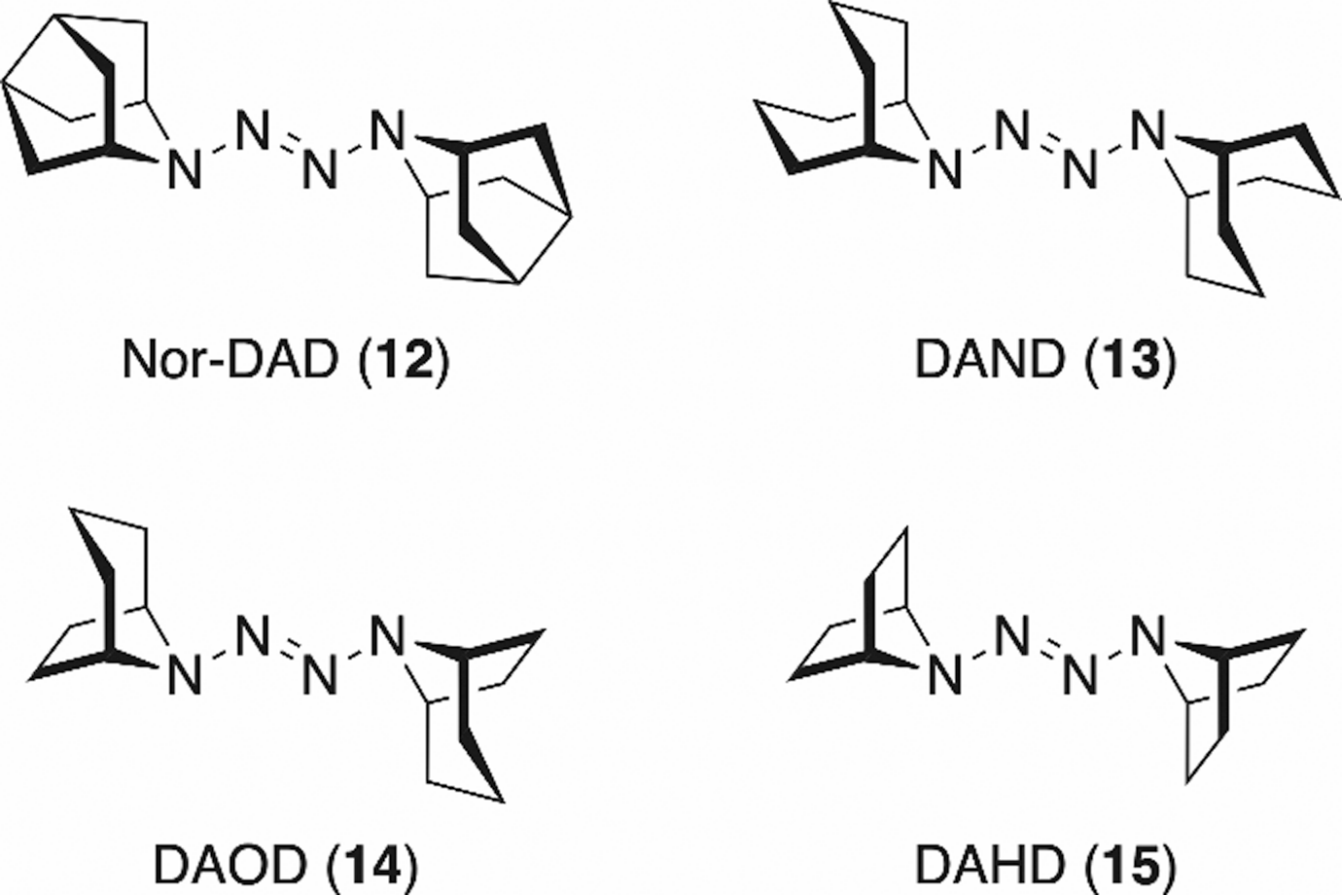
Cage-modified tetrazene derivatives.

The radical cations were then generated by oxidation with chlorine, followed by anion exchange with BArF^–^ under the same conditions as for DAD·BArF (**10**) (Scheme 2). Nor-DAD·BArF (**16**) and DAND·BArF (**17**) were obtained as red solids similar to **10**. By contrast, oxidation of DAOD (**14**) with chlorine/hydrogen chloride produced a red solid, presumably the corresponding radical cation·HCl_2_ salt, but this material gradually lost its color upon standing in air at room temperature, while anion exchange with BArF^–^ did not afford salt **18**. Furthermore, treatment of DAHD (**15**) with chlorine and hydrogen chloride yielded a white precipitate, and no radical cation formation was detected.

**Scheme 2:**
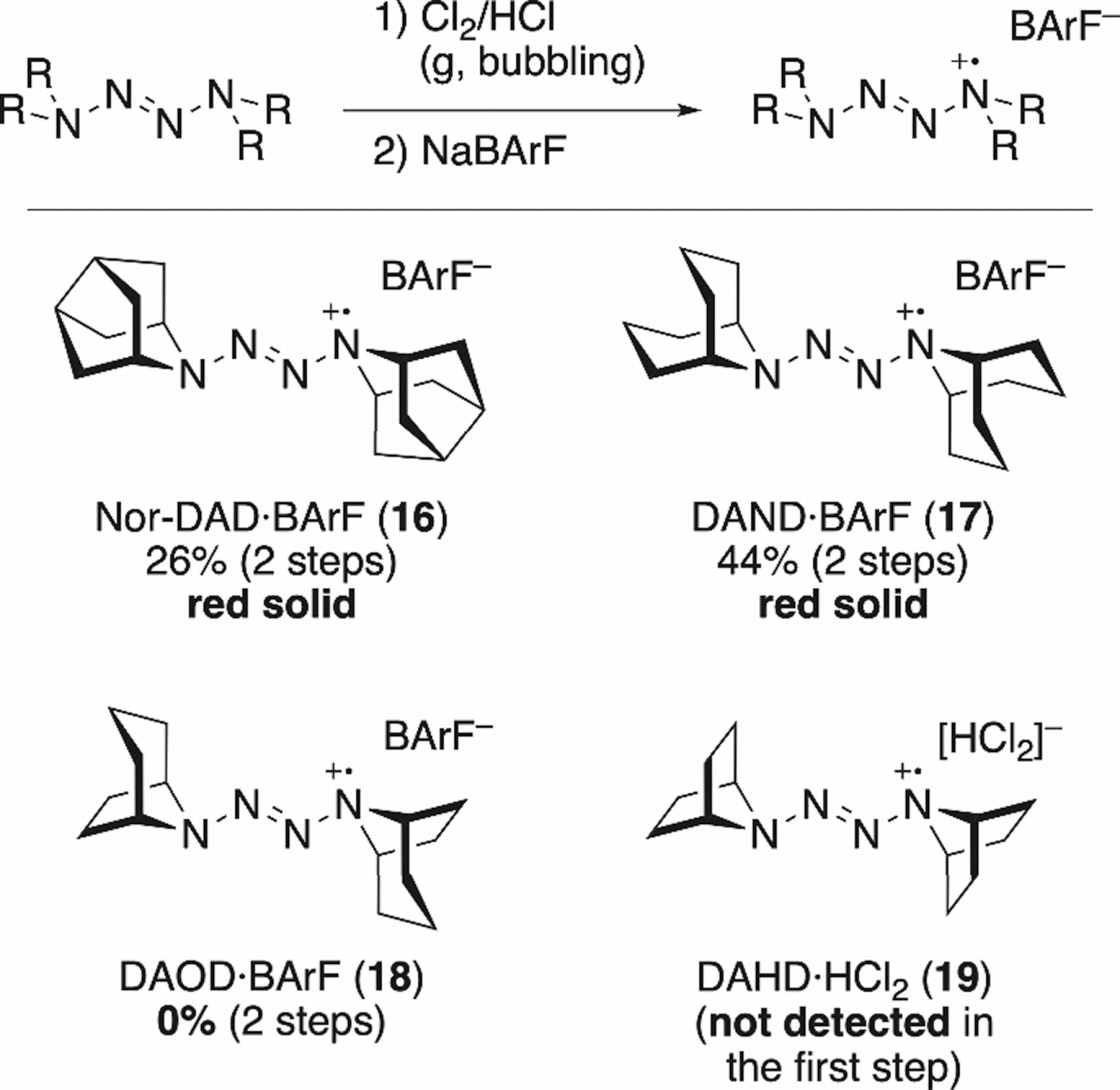
Attempts to Synthesize Tetrazene Radical Cation Salts

These results indicate that the main framework plays a crucial role in the stability of the tetrazene radical cation salts. In particular, the presence of a C–H bond at the γ-position of nitrogen—that is, a bicyclic framework incorporating a piperidine ring—appears to be essential. This requirement is consistent with the ESR results which revealed a significant spin density at the γ-position C–H bonds. Overall, these findings establish a clear structure–stability relationship for tetrazene radical cation salts, demonstrating that γ-position C–H bonds within a rigid bicyclic framework are a key structural requirement for the existence of air-stable, isolable form of these salts.

### Redox Properties and Catalytic Applications

Stable organic radicals generally exhibit distinct redox activity. In particular, nitroxyl radicals such as AZADO (**4**) have been widely recognized as highly useful catalysts in organic synthesis that owing to their unique redox properties enable efficient alcohol oxidation and other selective transformations.^1a,13^ Although the redox behavior of tetrazenes has previously been investigated by cyclic voltammetry (CV), their potential as oxidation catalysts has not been explored.^14^ In this study, we examined the redox properties of tetrazenes and their radical cation salts and evaluated their catalytic performance in alcohol oxidation. Electrochemical measurements and catalytic experiments were performed to elucidate the influence of the structural features of the tetrazenes on their redox behaviors and catalytic activities.

To evaluate the redox properties of DAD (**8**), CV measurements were performed in acetonitrile (black trace in Figure 7). DAD (**8**) exhibited two well-separated reversible redox waves, with midpoint potentials of 0.01 and 0.95 V vs Fc/Fc^+^, assigned to (i) the **8**/**3** couple and (ii) the **3**/DAD dication **20** couple (Scheme 3).

**Figure 7:**
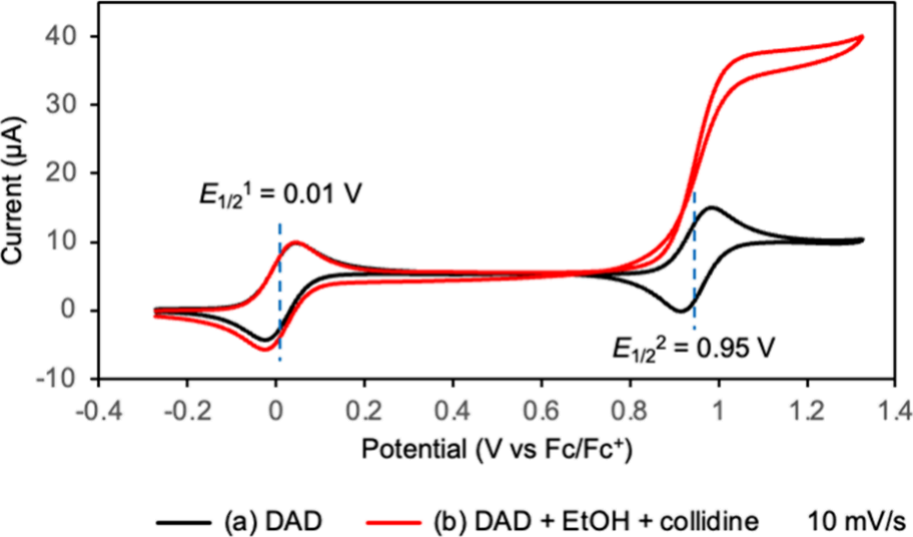
Cyclic voltammograms of DAD (1 mM) in the (a) absence and (b) presence of EtOH (100 mM) and 2,4,6-collidine (10 mM) in MeCN (containing 100 mM TBAPF_6_) at a scan rate of 10 mV s^–1^.

**Scheme 3:**
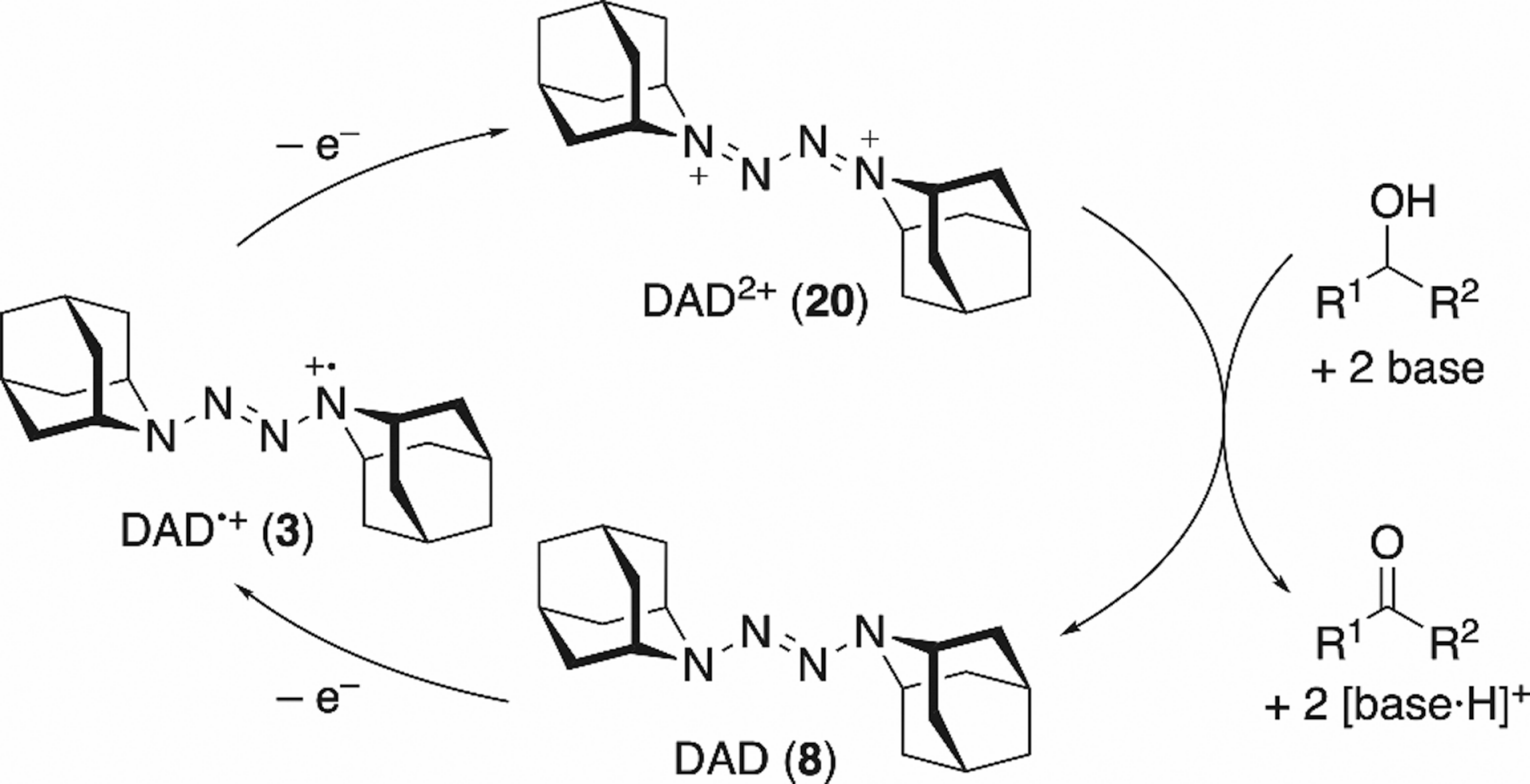
Plausible Mechanism

The catalytic activity of **8** for alcohol oxidation was examined by adding ethanol to an acetonitrile solution in the presence of 2,4,6-collidine as a base. Under these conditions, the oxidation current associated with the second redox wave increased markedly, and the corresponding reduction wave disappeared (red trace in Figure 7), indicating clear electrocatalytic behavior and implicating **20** as an active oxidant. These observations are consistent with a catalytic cycle in which **20** oxidizes alcohols to the corresponding carbonyl compounds while being reduced back to **8**, enabling **8** and **3** to catalyze alcohol oxidation (Scheme 3).

To evaluate the synthetic utility of the catalytic wave observed in the CV experiment, a preparative-scale constant-current electrochemical oxidation of menthol was conducted using a catalytic amount of **8** (Scheme 4). Menthol is a sterically hindered secondary aliphatic alcohol that is difficult to oxidize.^8d^ Under these conditions, the reaction produced menthone in 90% yield, demonstrating that the catalytic cycle involving **8**, **3**, and **20** (Scheme 3) operates effectively under preparative conditions. These electrochemical findings prompted us to investigate whether the same catalytic system could operate under nonelectrochemical conditions.

**Scheme 4:**
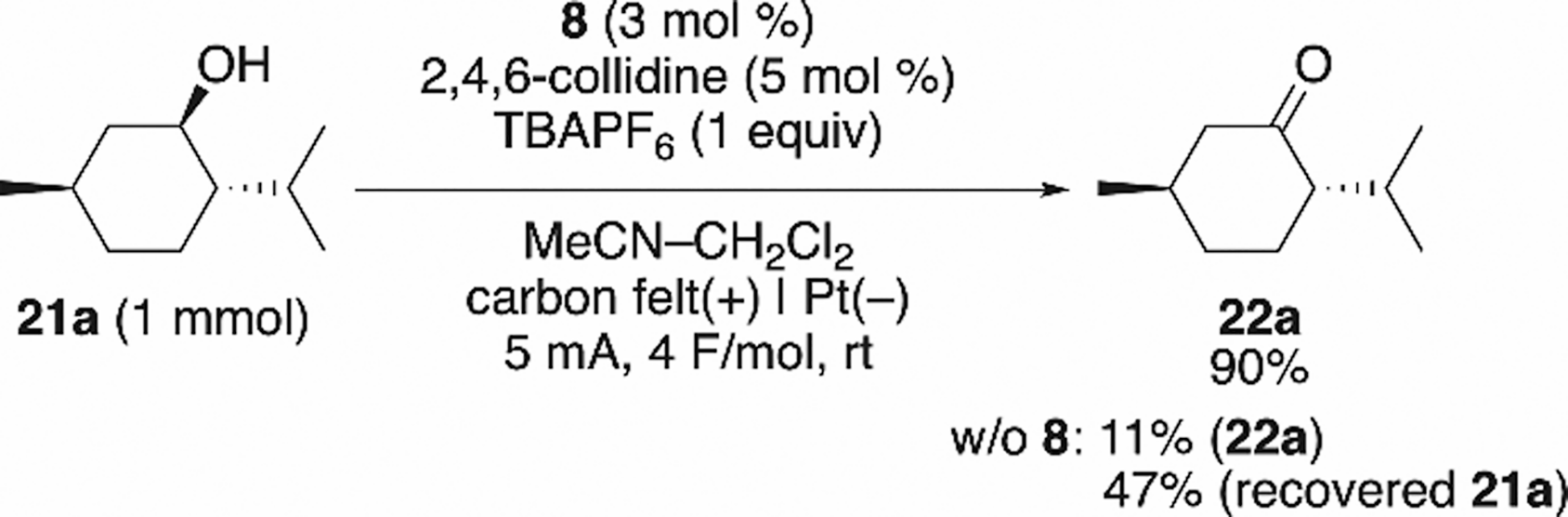
Preparative-Scale Constant-Current Electrochemical Oxidation of Menthol Catalyzed by DAD

Building on these electrochemical observations, we assessed the catalytic performance of DAD (**8**) and its radical cation salts **9**, **10**, and **11** in alcohol oxidation under nonelectrochemical conditions. The catalytic activities of **8**, **9**, **10**, and **11** were evaluated for the oxidation of menthol using sodium hypochlorite as the terminal oxidant (Table 2). After reaction time of 30 min, 0.5 mol % AZADO (**4**) promoted the reaction with a conversion of 39%, while **8** gave a better (60%) conversion even with 0.25 mol % catalyst loading (entries 1–3, Table 2). To promote the formation of **20**, 10 mol % of KBr was added because it can generate a small amount of hypobromous acid (HOBr) from sodium hypochlorite, thereby accelerating oxidation.^15^ As a result, full conversion was achieved with only 0.1 mol % **8** (entry 4). The counteranions of the DAD radical cation affect the catalytic efficiency. Notably, 0.1 mol % HCl_2_ salt **9** and 0.1 mol % NTf_2_ salt **11** catalyzed the reaction for complete conversion, while 0.5 mol % BArF salt **10** did not complete the reaction (entries 5–7).^16^ When the catalyst loading of **8** was reduced, 0.02 mol % **8** gave a nearly complete (98%) conversion with a trace amount of unreacted substrate remaining (entry 8). Similarly, under KBr-added conditions, **4** showed comparable performance, affording 99% conversion at the same catalyst loading (0.02 mol %, entries 9 and 10).

**Table 2:**
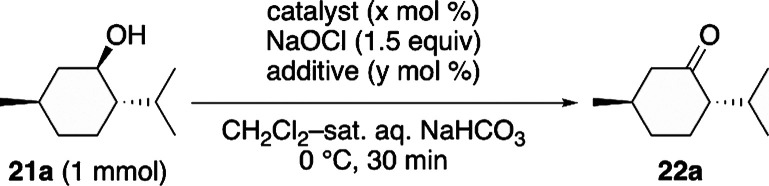
Evaluation of Catalytic Efficiency of Tetrazenes and Their Radical Cation Salts

entry	catalyst (x)	additive (y)	GC conv.
1	AZADO (0.5)	none	39%
2	DAD (0.5)	none	76%
3	DAD (0.25)	none	60%
4	DAD (0.1)	KBr (10)	100%
5	**9** (0.1)	KBr (10)	100%
6	**10** (0.5)	KBr (10)	94%^a^
7	**11** (0.1)	KBr (10)	100%
8	DAD (0.02)	KBr (10)	98%
9	AZADO (0.1)	KBr (10)	100%
10	AZADO (0.02)	KBr (10)	99%
11	Nor-DAD (0.1)	KBr (10)	100%
12	DAND (0.1)	KBr (10)	100%
13	DAOD (0.1)	KBr (10)	94%
14	DAHD (0.1)	KBr (10)	13%
15	none	KBr (10)	0%
16	none	none	0%

a Reaction time = 1 h.

The difference in the catalytic efficiency observed between HCl_2_ salt **9** and BArF salt **10**, can be attributed to differences in the rate of the reoxidation step, which is considered to be the rate-limiting step for alcohol oxidation under nitroxyl radical catalyst/sodium hypochlorite conditions.^17^ Because sodium hypochlorite is present in the aqueous phase, the reoxidation rate is expected to be correlated with the hydrophilicity of the catalyst. To test this hypothesis, the hydrophilicities of **9** and **10** were compared. In an ethyl acetate–water partition experiment, **10** was predominantly extracted into the organic phase, whereas **9** was extracted into the aqueous phase. Therefore, the higher hydrophilicity of **9** likely facilitated faster reoxidation, leading to its superior catalytic performance.

Encouraged by the high catalytic activity of DAD (**8**), we examined the effect of the main framework on the catalytic performance. Nor-DAD (**12**) and DAND (**13**) exhibited activities comparable to those of **8** (entries 11 and 12, Table 2), whereas DAOD (**14**) showed slightly diminished activity (entry 13), and DAHD (**15**) displayed a markedly lower activity (entry 14). This order of catalytic efficiency correlates well with the stability trends of the corresponding radical cations described above, with more stable radical cations showing higher catalytic activity. Furthermore, both the radical cation stability and catalytic activity were correlated with the shape of the CV traces (see Supporting Information for chromatograms). Tetrazenes **12** and **13** displayed CV profiles nearly identical to those of **8**, whereas **14** exhibited somewhat distorted voltammograms upon ethanol addition, and **15** showed no evidence of the two reversible redox processes. Notably, under control conditions without tetrazene or AZADO (**4**), formation of menthone was not observed (entries 15 and 16).

With the optimal reaction conditions in hand, the substrate scope of the **8**- and **9**-catalyzed alcohol oxidation reactions was examined (Table 3). Both catalysts yielded nearly identical results across the tested substrates. Bulky secondary alcohols **21a** and **21c**–**21e** were oxidized to the corresponding ketones in good yields with 0.1–0.5 mol % of either **8** or **9**. The reaction also tolerated a variety of functional groups, including silyl ether (**21d**), acetal (**21e**), ester (**21f**), carbamate (**21g**), and nitrobenzene (**21h**). Primary alcohols **21h** and **21i** were efficiently oxidized to their corresponding aldehydes in high yields. These results highlight the broad applicability and high efficiencies of both catalysts for alcohol oxidation.

**Table 3:**
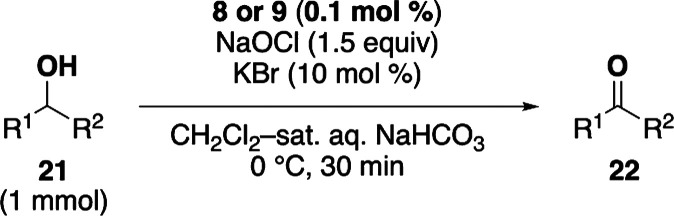

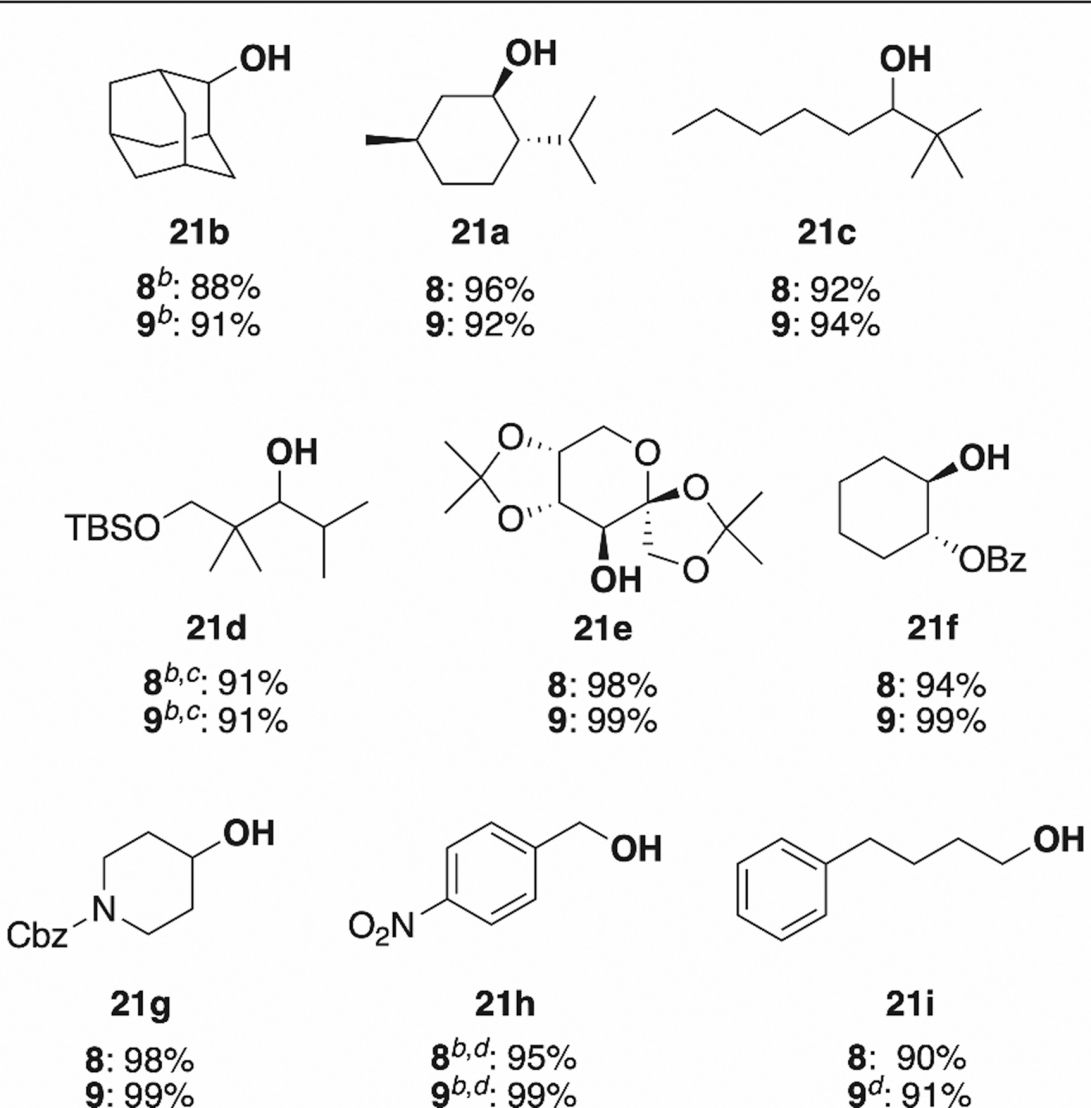
Scope of DAD- and DAD·HCl_2_-Catalyzed Alcohol Oxidation^a^

a Structures of substrates and isolated yields of carbonyl products.

b Reaction time of 1 h.

c 0.5 mol % of **8** or **9**.

d 1.2 equiv of NaOCl.

To gain mechanistic insight into **8**-catalyzed alcohol oxidation, we next performed a series of experiments to examine the catalyst integrity and the nature of the active oxidant. To verify whether the catalyst maintains the tetrazene framework under the reaction conditions, the large-scale oxidation of isopropanol (183 mmol) with sodium hypochlorite using **8** (25 mg) as the catalyst was conducted, and the catalyst-derived materials were carefully analyzed after completion of the reaction (Scheme 5a). Compound **8** was recovered in 92% yield, and no other isolable decomposition products, including AZADO (**4**), were detected. These results indicate that **8** functions as a catalyst while retaining the tetrazene structure throughout the reaction. In a complementary experiment, the stoichiometric oxidation of menthol with DAD·BArF (**10**) was examined (Scheme 5b). Menthone formation was not observed, indicating that the radical cation itself does not have sufficient oxidizing ability toward alcohols. This observation is consistent with the CV data (Figure 7, red trace). Together, these findings support the hypothesis that sodium hypochlorite generates dication **20** under the reaction conditions, analogous to the process occurring under electrochemical oxidation (Scheme 3). Dication **20** is therefore considered the most likely active oxidant in the catalytic cycle.^18^

**Scheme 5:**
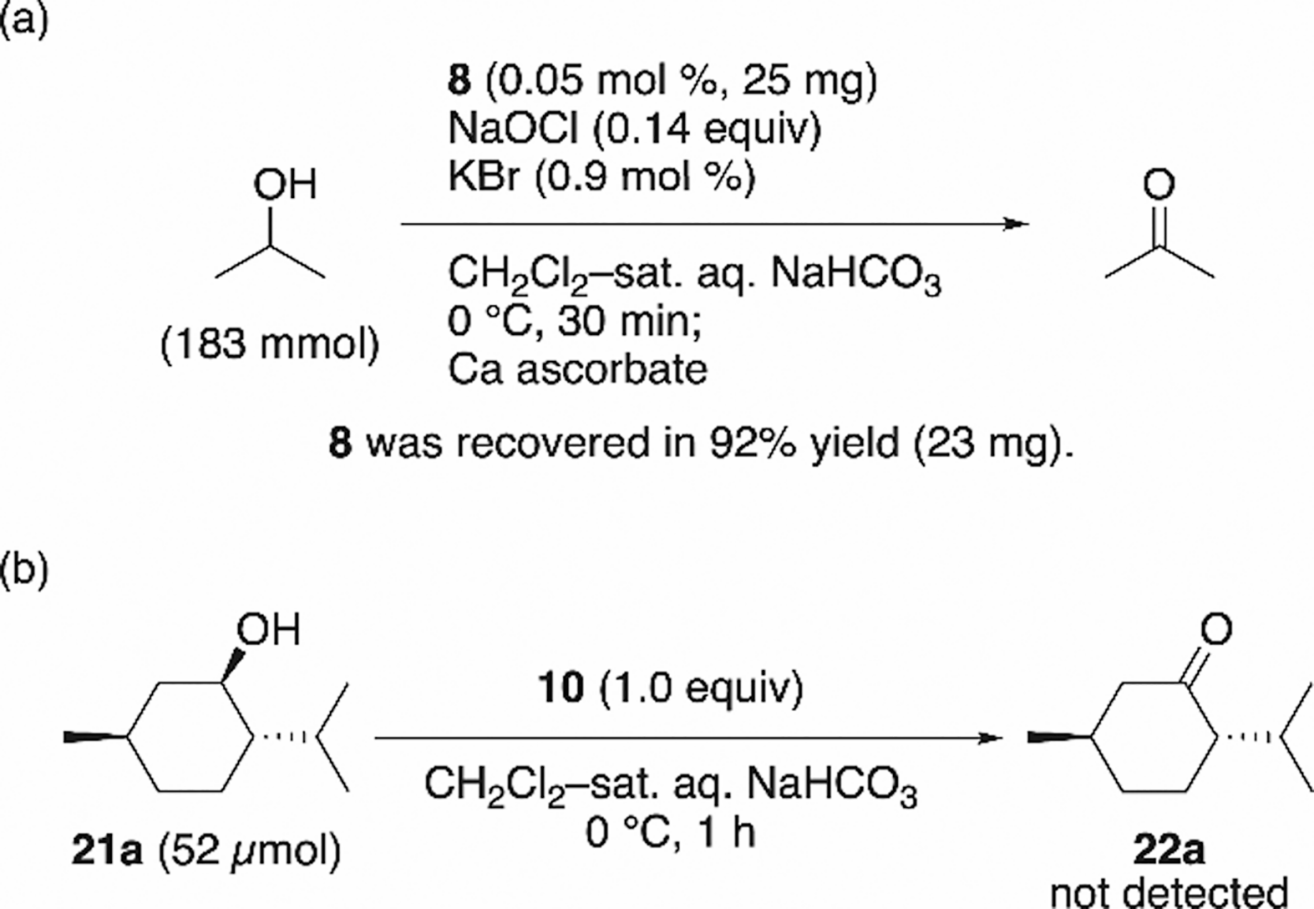
Mechanistic Studies on DAD-Catalyzed Alcohol Oxidation^a^

a (a) Recovery and analysis of catalyst-derived materials after large-scale oxidation. (b) Stoichiometric oxidation of menthol with the DAD radical cation salt to evaluate its intrinsic oxidizing activity.

## Conclusions

In summary, we synthesized and structurally characterized stable tetrazene radical cation salts capped with adamantane, representing the first example of the isolation and crystal structure determination of a tetrazene radical cation. Systematic variation of the cage framework revealed that a bicyclic piperidine-type structure in which γ-position C–H bonds participate in spin delocalization is a key structural requirement for stability under ambient conditions. Notably, the DAD radical cations are stable despite the absence of aromatic rings, exhibit two well-separated reversible redox waves, and serve as highly efficient catalysts for the oxidation of alcohols to carbonyl compounds under mild conditions. Furthermore, the choice of the counteranion was found to significantly affect both the isolability and catalytic performance of the radical cations, underscoring the importance of the counteranion as a design element. These findings not only establish design principles for stable nitrogen-containing radical cations but also expand the range of their potential applications in redox catalysis.

## Supplementary Material



## Notes

Experimental details and copies of NMR spectra (PDF)
