# Supplementary material for: Air-Stable Tetrazene Radical Cation Salts: Structural Requirements and Oxidation Catalysts
Source: J Am Chem Soc. 2025 Dec 15;148(1):632–9. doi: 10.1021/jacs.5c15272 (PMC12814318; doi:10.1021/jacs.5c15272)

Supporting Information

Air-stable Tetrazene Radical Cation Salts: Structural Requirements and Oxidation Catalysts

Ayari Oshiro,[†] Yusuke Sasano,*[,†] Shu Saito,[†] Yasuyuki Araki,[‡] Soichiro Sugiyama,[§] Eunsang Kwon,[‖] Shinji Kajimoto,[†] Yuse Kuriyama,[†] Shohei Yoshinaga,[⊥] Masaya Takahashi,[†] Katsuhiko Sato,[#] Naoki Shida,[⊥] Yusuke Ishigaki,[§] Mahito Atobe,[⊥] and Yoshiharu Iwabuchi*[,†]

[†]Graduate School of Pharmaceutical Sciences, Tohoku University, 6-3 Aoba, Aramaki, Aoba-ku, Sendai 980-8578, Japan

[‡]Institute of Multidisciplinary Research for Advanced Materials (IMRAM), Tohoku University, 2-1-1 Katahira, Aoba-ku, Sendai, 980-8577, Japan

[§]Department of Chemistry, Faculty of Science, Hokkaido University, Sapporo 060-0810, Japan

[‖]Research and Analytical Center for Giant Molecules, Graduate School of Science, Tohoku University, 6-3 Aoba, Aramaki, Aoba-ku, Sendai 980-8578, Japan

[⊥]Graduate School of Science and Engineering, Yokohama National University, 79-5 Tokiwadai, Hodogaya-ku, Yokohama 240-8501, Japan

[#]Faculty of Pharmaceutical Science, Tohoku Medical and Pharmaceutical University, 4-4-1 Komatsushima, Aoba-ku, Sendai 981-8558, Japan

Table of Contents

1. General Methods · · · S3

2. Synthesis of Tetrazenes and Their Radical Cation Salts · · · S4

3. X-ray Crystallography and Related Theoretical Calculations · · · S18

4. ESR Analysis and Related Theoretical Calculations · · · S33

5. Cyclic Voltammetry · · · S38

6. Preparative-Scale Electrochemical Oxidation · · · S41

7. Availability of Alcohol Substrates · · · S43

8. General Procedures for DAD-catalyzed Alcohol Oxidation · · · S45

9. Purification Methods and Spectral Data of Carbonyl Products · · · S46

10. Recovery and Analysis of Catalyst-Derived Materials · · · S50

11. Differential Scanning Calorimetry Analysis · · · S51

12. References · · · S52

13. ^{1}H and ^{13}C NMR Spectral Copies · · · S55

1. General Methods

All reactions were carried out under an argon atmosphere with dehydrated solvents under anhydrous conditions, unless otherwise noted. Dehydrated THF and CH_2Cl_2 were purchased from Kanto Chemical Co., Inc. Other solvents were dehydrated and distilled according to standard protocols. Reagents were obtained from commercial suppliers and used without further purification, unless otherwise noted.

Reactions were monitored by thin-layer chromatography (TLC) carried out on 0.25 mm silica gel plates (Merck $60F_{254}$) or by gas chromatography (GC) using an Agilent 7890A GC system equipped with a flame ionization detector (FID) and an Agilent HP-5 capillary column (30 m × 0.320 mm i.d., 0.25 μm film thickness). Column chromatography was performed using silica gel (Kanto Chemical Co., Inc., Silica Gel 60N, spherical, neutral, particle size 63–210 μm), unless otherwise noted.

Infrared (IR) spectra were recorded on a JASCO FT/IR-4600 spectrometer and are reported in wavenumbers (cm^{-1}). UV–vis absorption spectra were recorded on a Hitachi U-3310 spectrophotometer. Proton nuclear magnetic resonance (^{1}H NMR) spectra were recorded on a Varian 400-MR (400 MHz) or a JEOL JNM-ECZL400S (400 MHz) spectrometer. Chemical shifts (δ) are reported in parts per million (ppm) relative to tetramethylsilane (TMS; 0.00 ppm) in $CDCl_3$. Coupling constants (J) are reported in hertz (Hz). Carbon-13 nuclear magnetic resonance (^{13}C NMR) spectra were recorded on a Varian 400-MR (100 MHz) spectrometer. Chemical shifts are reported in ppm relative to the center line of the triplet of $^{13}CDCl_3$ (77.0 ppm). Low- and high-resolution mass spectra (MS, HRMS) were recorded on a JEOL JMS-700 or JMS-T100GC for electron impact (EI) and on a JEOL JMS-T100LP for fast atom bombardment (FAB). Elemental analyses were performed on a Yanaco CHN CORDER MT-6.

2. Synthesis of Tetrazenes and Their Radical Cation Salts

Synthesis of 1,2-di(2-azaadamantan-2-yl)diazene (DAD) radical cation salts

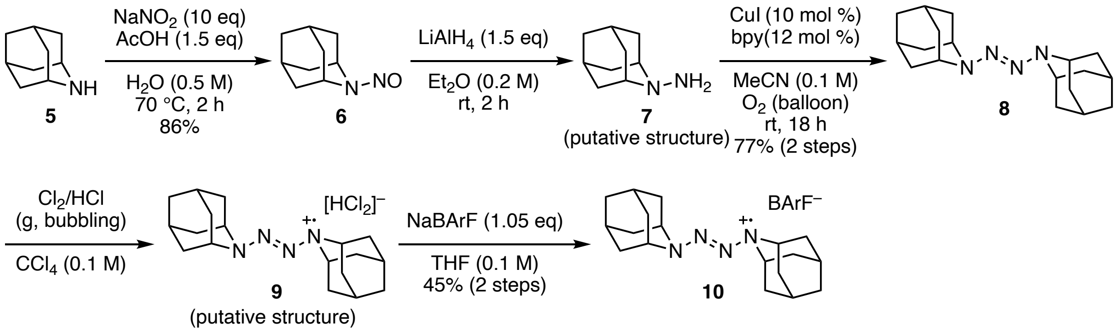

2-Nitroso-2-azaadamantane (6)

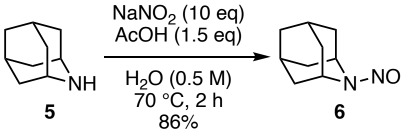

To a solution of 2-azaadamantane[1] (500 mg, 3.64 mmol) in H_2O (7.3 mL) were added AcOH (0.33 mL, 5.8 mmol) and $NaNO_2$ (2.51 g, 36.4 mmol) at room temperature, and the mixture was stirred at 70 °C for 2 h. After cooling to room temperature, the mixture was extracted with Et_2O. The combined organic layers were washed with brine, dried over Na_2SO_4, and concentrated under reduced pressure. The residue was purified by column chromatography (AcOEt/*n*-hexane = 1:5 → 1:2) to give nitrosoamine **6** (520 mg, 3.13 mmol, 86%) as a yellow solid.

IR (neat, cm^{-1}): 2927, 2855, 1424, 1374, 1337, 1290, 1268, 1189, 1086, 1024, 950, 758; ^{1}H NMR (400 MHz, $CDCl_3$): δ 5.38 (s, 1H), 4.93 (s, 1H), 2.19 (s, 2H), 2.02 (s, 4H), 1.99–1.93 (m, 2H), 1.81 (d, J = 12.8 Hz, 2H), 1.73 (d, J = 12.8 Hz, 2H); ^{13}C NMR (100 MHz, $CDCl_3$): δ 55.2, 44.3, 36.7, 35.3, 34.9, 27.1; MS (EI) *m/z*: 166 (M^+), 136 (100%); HRMS–EI (*m/z*): M^+ calcd for $C_9H_{14}N_2O$, 166.1106; found, 166.1108.

1,2-Di(2-azaadamantan-2-yl)diazene (8)

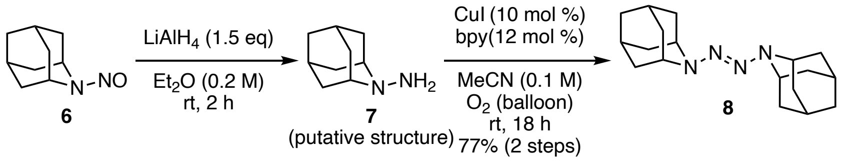

To a solution of $LiAlH_4$ (68.5 mg, 1.80 mmol) in Et_2O (3.0 mL) was added a solution of nitrosoamine **6** (200 mg, 1.20 mmol) in Et_2O (3.0 mL) at 0 °C. The mixture was stirred at room temperature for 2 h, then H_2O and 10% aqueous NaOH were added slowly at 0 °C, and Et_2O was added to allow efficient stirring. After being stirred at room temperature for 30 min, the mixture was filtered through a Celite pad. The filtrate was concentrated under reduced pressure to give putative hydrazine **7** (182 mg) as a colorless solid, which was used in the next reaction without further purification.

CuI (22.8 mg, 0.120 mmol) and 2,2′-bipyridine (22.4 mg, 0.143 mmol) were dissolved in acetonitrile (6.0 mL) open to the air. A solution of putative hydrazine **7** (182 mg) in acetonitrile (6.0 mL) was then slowly added over 3.5 h using a syringe pump at room temperature, and the mixture was stirred for 18 h under an O_2 atmosphere. TMEDA (35.8 μL, 0.239 mmol) was added, and the mixture was stirred for a few minutes. The mixture was concentrated under reduced pressure, diluted with water, and extracted with CH_2Cl_2. The combined organic layers were washed with brine, dried over Na_2SO_4, and concentrated under reduced pressure. The residue was purified by column chromatography (AcOEt/*n*-hexane = 1:20) to give tetrazene **8** (139 mg, 0.463 mmol, 77%, 2 steps) as a colorless solid.

IR (neat, cm^{-1}): 2922, 2846, 1447, 1376, 1083, 1004, 945; ^1H NMR (400 MHz, $CDCl_3$): δ 4.07 (s, 4H), 2.02 (s, 4H), 2.00 (d, *J* = 11.5 Hz, 8H), 1.84 (s, 4H), 1.66 (d, *J* = 11.5 Hz, 8H); ^{13}C NMR (100 MHz, $CDCl_3$): δ 51.1, 36.5, 34.0, 27.3; MS (EI) *m/z*: 300 (M^+), 80 (100%); HRMS–EI (*m/z*): M^+ calcd for $C_{18}H_{28}N_4$, 300.2314; found, 300.2308.

DAD radical cation tetrakis[3,5-bis(trifluoromethyl)phenyl]borate (BArF) salt (10)

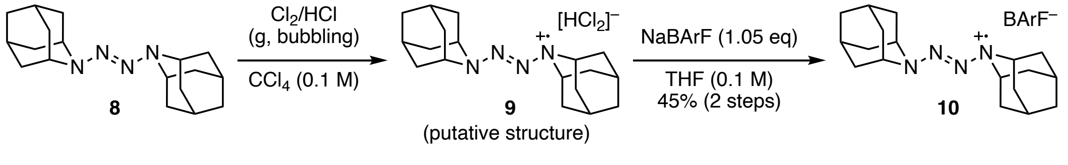

Chlorine/hydrogen chloride mixed gas, generated by adding concentrated hydrochloric acid to MnO_2 at 50 °C, was bubbled into a solution of tetrazene **8** (100 mg, 333 μmol) in CCl_4 (3.3 mL) at room temperature until no further precipitate formed. The resulting insoluble red solid was collected by filtration and washed several times with Et_2O. The solid on the glass filter was rinsed into a round-bottom flask with CH_2Cl_2, and concentrated under reduced pressure to give putative hydrogen dichloride salt **9** (89.2 mg) as a red solid, which was used in the next reaction without further purification.

To a solution of putative hydrogen dichloride salt **9** (89.2 mg) in THF (2.7 mL) was added NaBArF (247 mg, 0.279 mmol) at 0 °C. The mixture was stirred at room temperature for 2 h, then water was added, and the mixture was extracted with CH_2Cl_2 (3 ×). The combined organic layers were dried over Na_2SO_4 and concentrated under reduced pressure. The residue was purified by column chromatography (AcOEt/*n*-hexane = 1:2 → MeOH/$CHCl_3$ =1:20) to give BArF salt **10** (175 mg, 0.150 mmol, 45%) as a red solid.
Anal. Calcd for $C_{18}H_{28}N_4$: C, 51.61; H, 3.46; N, 4.81. Found: C, 51.51; H, 3.60; N, 4.86. IR (neat, cm^{-1}): 2943, 1608, 1450, 1354, 1277, 1120, 893, 837, 775, 714, 675; MS (FAB) *m/z*: 300 ($DAD^{•+}$), 136 (100%); HRMS–FAB (*m/z*): $DAD^{•+}$ calcd for $C_{18}H_{28}N_4$,300.2314; found, 300.2327.

DAD radical cation bis(trifluoromethanesulfonyl)imide (NTf_2) salt (11)

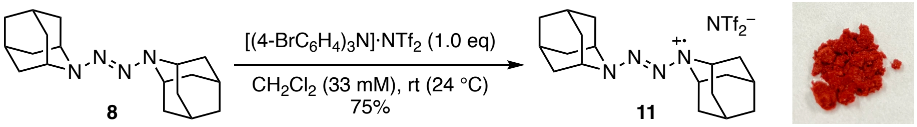

To a solution of tetrazene **8** (10.0 mg, 33.3 μmol) in CH_2Cl_2 (1.0 mL) was added tris(4-bromophenyl)ammoniumyl bis(trifluoromethanesulfonyl)imide[2] (25.4 mg, 33.3 μmol) at room temperature (24 °C) and the mixture was stirred for 10 min. The solvent was then removed under reduced pressure. Upon addition of Et_2O, the radical cation salt precipitated. The precipitate was collected by filtration, washed three times with Et_2O, and dried in vacuo to give NTf_2 salt **11** (14.5 mg, 25.0 μmol, 75%) as a red solid.

A single crystal suitable for X-ray diffraction analysis was obtained by recrystallization from Et_2O (see Section 3-4 for details).
Anal. Calcd for $C_{20}H_{28}F_6N_5O_4S_2$: C, 41.38; H, 4.86; N, 12.06. Found: C, 41.27; H, 4.79; N, 12.09; IR (neat, cm^{-1}): 2933, 2864, 1450, 1352, 1180, 1059, 947; MS (FAB) *m/z*: 300 ($DAD^{•+}$), 300 (100%); HRMS–FAB (*m/z*): $DAD^{•+}$ calcd for $C_{18}H_{28}N_4$, 300.2314; found, 300.2322.

Synthesis of 1,2-di(9-azanoradamantane-9-yl)diazene (Nor-DAD) radical cation salts

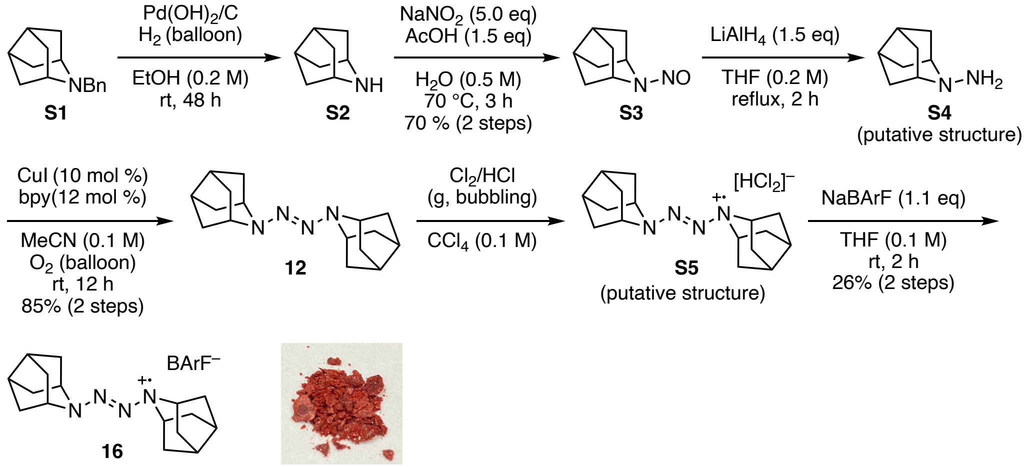

9-Nitroso-9-azanoradamantane (S3)

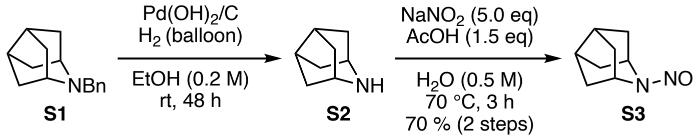

To a solution of benzylamine **S1**[3] (602 mg, 2.82 mmol) in EtOH (14 mL) was added $Pd(OH)_2/C$ (20% Pd, wetted with ca. 50% water, 60.2 mg) under an Ar atmosphere. The flask was evacuated and purged with H_2 three times, and the mixture was stirred at room temperature under a H_2 atmosphere (balloon) for 48 h. The Pd catalyst was removed by filtration through a Celite pad, and the filtrate was concentrated under reduced pressure to give amine **S2** (385 mg) as a yellow solid, which was used in the next reaction without further purification.

To a solution of amine **S2** (385 mg) in H_2O (6.2 mL) were added AcOH (268 μL, 4.69 mmol) and $NaNO_2$ (1.08 g, 15.6 mmol) at room temperature, and the mixture was stirred at 70 °C for 3 h. After cooling to room temperature, the mixture was extracted with AcOEt. The combined organic layers were washed with brine, dried over Na_2SO_4, and concentrated under reduced pressure. The residue was purified by column chromatography (AcOEt/*n*-hexane = 1:10 → 1:5) to give nitrosoamine **S3** (300 mg, 1.97 mmol, 70%, 2 steps) as a pale yellow solid.

IR (neat, cm^{-1}): 2973, 2920, 2879, 1456, 1418, 1354, 1321, 1308, 1274, 1237, 1179, 1076, 1040, 987, 964, 917, 788, 764; ^1H NMR (400 MHz, $CDCl_3$): δ 5.62 (s, 1H), 5.20 (s, 1H), 2.89 (quint, *J* = 5.6 Hz, 2H), 1.97–1.87 (m, 2H), 1.84–1.72 (m, 4H), 1.56 (d, *J* = 12.0 Hz,

2H); ^{13}C NMR (100 MHz, $CDCl_3$): δ 64.0, 52.7, 42.6, 41.8, 36.3; MS (EI) *m/z*: 152 (M^+), 95 (100%); HRMS–EI (*m/z*): M^+ calcd for $C_8H_{12}N_2O$, 152.0950; found, 152.0949.

1,2-Di(9-azanoradamantane-9-yl)diazene (Nor-DAD) (12)

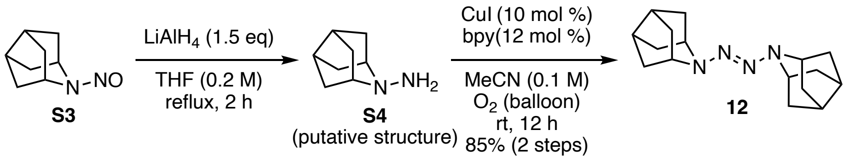

To a solution of $LiAlH_4$ (75.2 mg, 1.98 mmol) in THF (2.6 mL) was added a solution of nitrosoamine **S3** (201 mg, 1.32 mmol) in THF (4.0 mL) at 0 °C. The mixture was stirred at reflux for 2 h, then H_2O and 10% aqueous NaOH were added slowly at 0 °C, and CH_2Cl_2 was added to allow efficient stirring. After being stirred at room temperature for 30 min, the mixture was filtered through a Celite pad. The filtrate was concentrated under reduced pressure to give putative hydrazine **S4** (190 mg) as a yellow solid, which was used in the next reaction without further purification.

CuI (26.2 mg, 137 μmol) and 2,2′-bipyridine (25.8 mg, 165 μmol) were dissolved in acetonitrile (7.7 mL) open to the air. A solution of putative hydrazine **S4** (190 mg) in acetonitrile (6.0 mL) was then slowly added over 3.5 h using a syringe pump at room temperature. The flask was evacuated and purged with O_2 three times, and the mixture was stirred at room temperature under an O_2 (balloon) atmosphere for 12 h. TMEDA (41.2 μL, 275 μmol) was added, and the mixture was stirred for a few minutes. The mixture was concentrated under reduced pressure, diluted with water, and extracted with CH_2Cl_2. The combined organic layers were washed with brine, dried over Na_2SO_4, and concentrated under reduced pressure. The residue was purified by column chromatography (AcOEt/*n*-hexane = 1:20) to give tetrazene **12** (153 mg, 562 μmol, 85%, 2 steps) as a colorless solid.

IR (neat, cm^{-1}): 2965, 2909, 2867, 1450, 1353, 1319, 1211, 1058, 1040, 1031, 964, 943, 903, 830, 783, 734; ^{1}H NMR (400 MHz, $CDCl_3$): δ 4.36 (s, 4H), 2.62 (quint, J = 5.5 Hz, 4H), 1.74 (d, J = 10.5 Hz, 8H), 1.66–1.56 (m, 8H); ^{13}C NMR (100 MHz, $CDCl_3$): δ 60.7, 40.8, 36.5; MS (EI) *m/z*: 272 (M^+), 67 (100%); HRMS–EI (*m/z*): M^+ calcd for $C_{16}H_{24}N_4$, 272.2001; found, 272.2003.

Nor-DAD radical cation BArF salt (16)

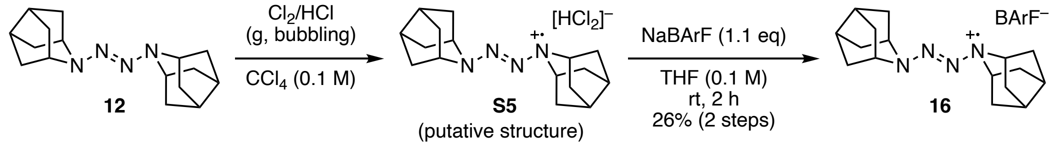

Chlorine/hydrogen chloride mixed gas, generated by adding concentrated hydrochloric acid to MnO_2 at 50 °C, was bubbled into a solution of tetrazene **12** (67.6 mg, 248 μmol) in CCl_4 (2.5 mL) at room temperature until no further precipitate formed. The resulting insoluble red solid was collected by filtration and washed several times with CCl_4. The solid on the glass filter was rinsed into a round-bottom flask with CH_2Cl_2 and concentrated under reduced pressure to give putative hydrogen dichloride salt **S5** (70.9 mg) as a red solid.

To a solution of putative hydrogen dichloride salt **S5** (70.9 mg) in THF (2.0 mL) was added NaBArF (201 mg, 227 μmol) at 0 °C. The mixture was stirred at room temperature for 2 h, then water was added, and the mixture was extracted with CH_2Cl_2 (5 ×). The combined organic layers were dried over Na_2SO_4 and concentrated under reduced pressure. The residue was purified by column chromatography (AcOEt/*n*-hexane =1:2 → MeOH/$CHCl_3$ =1:20) to give BArF salt **16** (73.9 mg, 65.1 μmol, 26%, 2 steps) as a red solid.

Anal. Calcd for $C_{48}H_{40}BF_{24}N_4$: C, 50.77; H, 3.20; N, 4.93. Found: C, 50.79; H, 3.39; N, 4.89; IR (neat, cm^{-1}): 2979, 1606, 1354, 1277, 1122, 887, 766, 714, 673; MS (FAB) *m/z*: 272 (Nor-DAD$^{\bullet+}$), 124 (100%); HRMS–FAB (*m/z*): Nor-DAD$^{\bullet+}$ calcd for $C_{16}H_{24}N_4$, 272.2001; found, 272.2012.

Synthesis of 1,2-di(9-azabicyclo[3.3.1]nonan-9-yl)diazene (DAND) radical cation salts

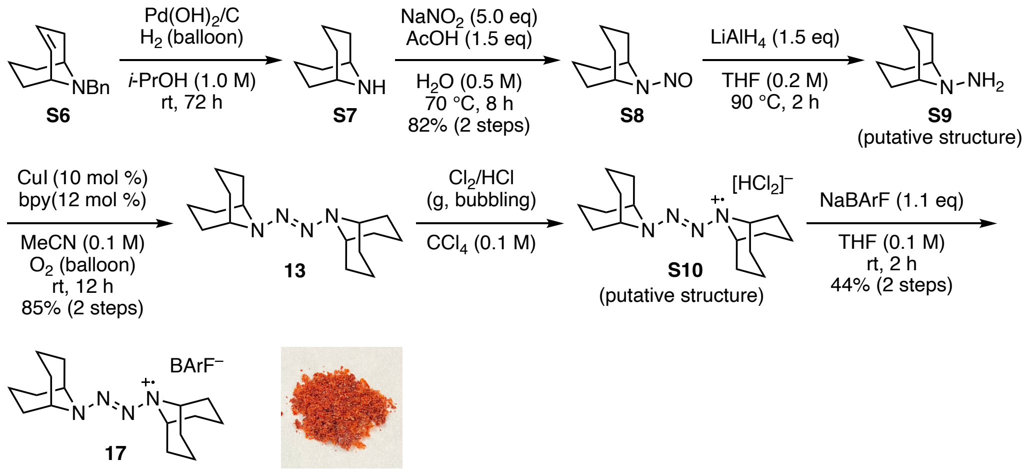

9-Nitroso-9-azabicyclo[3.3.1]nonane (S8)

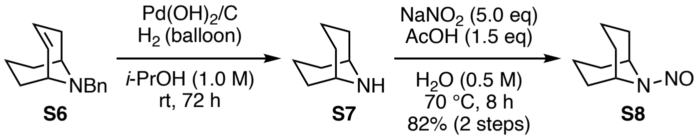

To a solution of benzylamine **S6**[4] (1.00 g, 4.69 mmol) in *i*-PrOH (4.7 mL) was added $Pd(OH)_2$/C (20% Pd, wetted with ca. 50% water, 100 mg) under an Ar atmosphere. The flask was evacuated and purged with H_2 three times, and the mixture was stirred at room temperature under a H_2 atmosphere (balloon) for 72 h. The Pd catalyst was removed by filtration through a Celite pad, and the filtrate was concentrated under reduced pressure to give amine **S7** (580 mg) as a colorless solid, which was used in the next reaction without further purification.

To a solution of amine **S7** (580 mg) in H_2O (9.3 mL) were added AcOH (397 μL, 6.95 mmol) and $NaNO_2$ (1.60 g, 23.2 mmol) at room temperature, and the mixture was stirred at 70 °C for 8 h. After cooling to room temperature, the mixture was extracted with AcOEt. The combined organic layers were washed with brine, dried over Na_2SO_4, and concentrated under reduced pressure. The residue was purified by column chromatography (AcOEt/*n*-hexane = 1:10 → 1:5) to give nitrosoamine **S8** (595 mg, 3.86 mmol, 82%, 2 steps) as a pale yellow solid.

IR (neat, cm^{-1}): 2947, 2916, 2854, 1423, 1369, 1277, 1192, 1111, 1018, 914, 733; ^1H NMR (400 MHz, $CDCl_3$): δ 5.34–5.24 (m, 1H), 4.86 (s, 1H), 2.26–1.93 (m, 6H), 1.79–1.71 (m, 4H), 1.69–1.59 (m, 2H); ^{13}C NMR (100 MHz, $CDCl_3$): δ 54.3, 43.3, 31.3, 29.2,

20.0; MS (EI) *m/z*: 154 (M^+), 96 (100%); HRMS–EI (*m/z*): M^+ calcd for $C_8H_{14}N_2O$, 154.1106; found, 154.1106.

1,2-Di(9-azabicyclo[3.3.1]nonan-9-yl)diazene (DAND) (13)

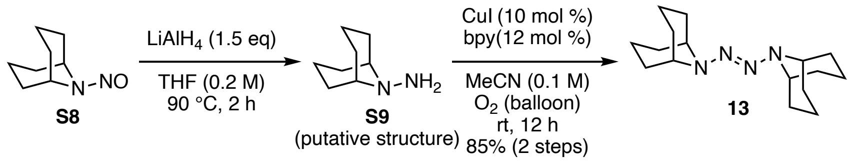

To a solution of $LiAlH_4$ (92.3 mg, 2.43 mmol) in THF (3.0 mL) was added a solution of nitrosoamine **S8** (250 mg, 1.62 mmol) in THF (5.0 mL) at 0 °C. The mixture was stirred at 90 °C for 2 h, then H_2O and 10% aqueous NaOH were added slowly at 0 °C, and CH_2Cl_2 was added to allow efficient stirring. After being stirred at room temperature for 30 min, the mixture was filtered through a Celite pad. The filtrate was concentrated under reduced pressure to give putative hydrazine **S9** (252 mg) as a yellow oil, which was used in the next reaction without further purification.

Putative hydrazine **S9** (252 mg) was divided into two equal portions (126 mg each). CuI (17.1 mg, 89.9 μmol) and 2,2′-bipyridine (16.8 mg, 108 μmol) were dissolved in acetonitrile (3.0 mL) open to the air. A solution of putative hydrazine **S9** (126 mg, 899 μmol) in acetonitrile (6.0 mL) was then slowly added over 3 h using a syringe pump at room temperature. The flask was evacuated and purged with O_2 three times, and the mixture was stirred at room temperature under an O_2 (balloon) atmosphere for 12 h. TMEDA (26.9 μL, 180 μmol) was added, and the mixture was stirred for a few minutes. The two reaction mixtures were then combined and concentrated under reduced pressure. The residue was diluted with water and extracted with CH_2Cl_2. The combined organic layers were washed with brine, dried over Na_2SO_4, and concentrated under reduced pressure. The residue was purified by column chromatography (AcOEt/*n*-hexane = 1:10) to give tetrazene **13** (190 mg, 687 μmol, 85%, 2 steps) as a colorless solid.

IR (neat, cm^{-1}): 2927, 2846, 1435, 1367, 1300, 1244, 1103, 1076, 1012, 928, 895, 800, 748; ^1H NMR (400 MHz, $CDCl_3$): δ 4.05 (s, 4H), 2.15–1.95 (m, 12H), 1.66–1.56 (m, 12H); ^{13}C NMR (100 MHz, $CDCl_3$): δ 50.4, 28.2, 20.8; MS (EI) *m/z*: 276 (M^+), 96 (100%); HRMS–EI (*m/z*): M^+ calcd for $C_{16}H_{28}N_4$, 276.2314; found, 276.2326.

DAND radical cation BArF salt (17)

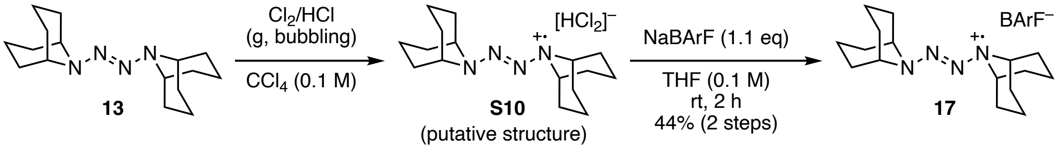

Chlorine/hydrogen chloride mixed gas, generated by adding concentrated hydrochloric acid to MnO_2 at 50 °C, was bubbled into a solution of tetrazene **13** (88.4 mg, 320 μmol) in CCl_4 (3.2 mL) at room temperature until no further precipitate formed. The resulting insoluble red solid was collected by filtration and washed several times with CCl_4. The solid on the glass filter was rinsed into a round-bottom flask with CH_2Cl_2 and concentrated under reduced pressure to give putative hydrogen dichloride salt **S10** (91.8 mg) as a red solid.

To a solution of putative hydrogen dichloride salt **S10** (91.8 mg) in THF (2.6 mL) was added NaBArF (257 mg, 290 μmol) at 0 °C. The mixture was stirred at room temperature for 2 h, then water was added, and the mixture was extracted with CH_2Cl_2 (3 ×). The combined organic layers were dried over Na_2SO_4 and concentrated under reduced pressure. The residue was purified by column chromatography (AcOEt/*n*-hexane= 1:2 → MeOH/$CHCl_3$ =1:20) to give BArF salt **17** (161 mg, 141 μmol, 44%, 2 steps) as a red solid.

Anal. Calcd for $C_{48}H_{40}BF_{24}N_4$: C, 50.59; H, 3.54; N, 4.92. Found: C, 50.56; H, 3.61; N, 4.93; IR (ATR, neat, cm^{-1}): 1352, 1277, 1165, 1122; MS (FAB) *m/z*: 276 ($DAND^{\bullet+}$), 124 (100%); HRMS–FAB (*m/z*): $DAND^{\bullet+}$ calcd for $C_{16}H_{28}N_4$, 276.2314; found, 276.2292.

Synthesis of 1,2-di(8-azabicyclo[3.2.1]octan-8-yl)diazene (DAOD) (14)

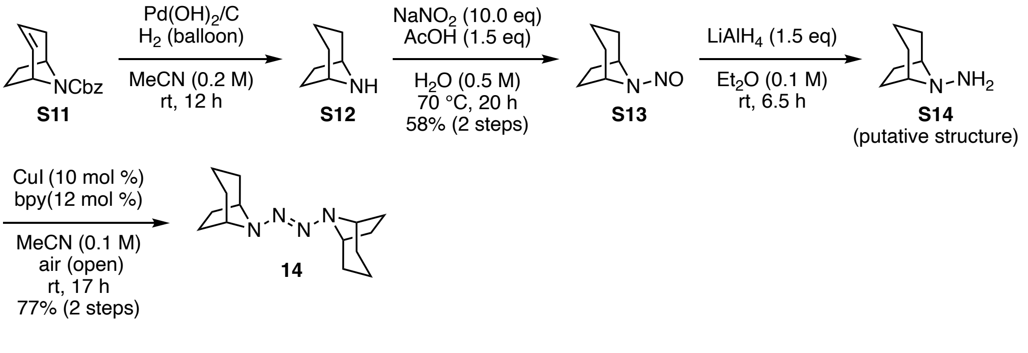

8-Nitroso-8-azabicyclo[3.2.1]octane (S13)

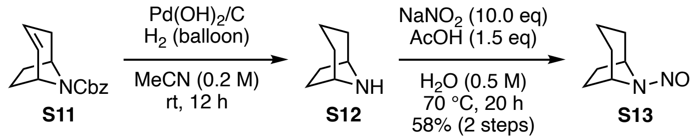

To a solution of carbamate **S11**[5] (1.43 g, 5.88 mmol) in acetonitrile (29 mL) was added $Pd(OH)_2/C$ (20% Pd, wetted with ca. 50% water, 143 mg) under an Ar atmosphere. The flask was evacuated and purged with H_2 three times, and the mixture was stirred at room temperature under a H_2 atmosphere (balloon) for 12 h. The Pd catalyst was removed by filtration through a Celite pad, and the filtrate was concentrated under reduced pressure to give 9-azabicyclo[3.3.1]nonane (**S12**) (697 mg) as a colorless solid, which was used in the next reaction without further purification.

To a solution of amine **S12** (697 mg) in H_2O (13 mL) were added AcOH (0.56 mL, 9.40 mmol) and $NaNO_2$ (4.32 g, 62.7 mmol) at room temperature, and the mixture was stirred at 70 °C for 20 h. After cooling to room temperature, the mixture was extracted with Et_2O. The combined organic layers were washed with brine, dried over Na_2SO_4, and concentrated under reduced pressure. The residue was purified by column chromatography (AcOEt/*n*-hexane = 1:5) to give nitrosoamine **S13** (479 mg, 3.42 mmol, 58%, 2 steps) as a pale yellow solid.

IR (neat, cm^{-1}): 2942, 2878, 2854, 1405, 1347, 1331, 1302, 1280, 1083, 1033, 951, 912, 841, 730; ^1H NMR (400 MHz, $CDCl_3$): δ 5.16–5.04 (m, 1H), 4.94 (d, J = 7.2 Hz, 1H), 2.24–2.10 (m, 1H), 2.00–1.45 (m, 9H); ^{13}C NMR (100 MHz, $CDCl_3$): δ 58.4, 50.6, 34.2, 30.4, 26.2, 25.6, 16.4; MS (EI) *m/z*: 140 (M^+), 110 (100%); HRMS–EI (*m/z*): M^+ calcd for $C_7H_{12}N_2O$, 140.0950; found, 140.0956.

1,2-Di(8-azabicyclo[3.2.1]octan-8-yl)diazene (DAOD) (14)

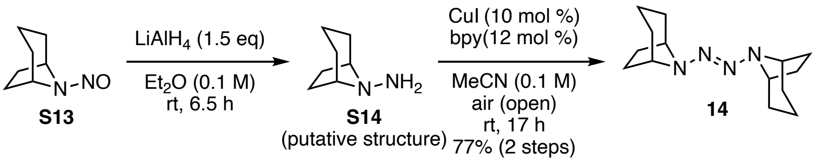

To a solution of $LiAlH_4$ (109 mg, 2.88 mmol) in Et_2O (13 mL) was added a solution of nitrosoamine **S13** (300 mg, 1.92 mmol) in Et_2O (6.0 mL) at 0 °C. The mixture was stirred at room temperature for 6.5 h, then H_2O and 10% aqueous NaOH were added slowly at 0 °C, and Et_2O was added to allow efficient stirring. After being stirred at room

temperature for 30 min, the mixture was filtered through a Celite pad. The filtrate was concentrated under reduced pressure to give putative hydrazine **S14** (262 mg) as a colorless solid, which was used in the next reaction without further purification.

CuI (39.5 mg, 208 μmol) and 2,2′-bipyridine (38.9 mg, 249 μmol) were dissolved in acetonitrile (8.0 mL) open to the air. A solution of putative hydrazine **S14** (262 mg) in acetonitrile (12.0 mL) was then slowly added over 7 h using a syringe pump at room temperature, and the mixture was stirred for 17 h under open air. TMEDA (62 μL, 0.41 mmol) was added, and the mixture was stirred for a few minutes. The mixture was concentrated under reduced pressure, diluted with water, and extracted with CH_2Cl_2. The combined organic layers were washed with brine, dried over Na_2SO_4, and concentrated under reduced pressure. The residue was purified by column chromatography (AcOEt/*n*-hexane = 1:15) to give tetrazene **14** (183 mg, 737 μmol, 77%, 2 steps) as a colorless solid. IR (neat, cm^{-1}): 2942, 2868, 1470, 1442, 1335, 1228, 1076, 1016, 967, 951, 755; ^1H NMR (400 MHz, $CDCl_3$): δ 4.17 (s, 4H), 1.93–1.81 (m, 4H), 1.81–1.71 (m, 4H), 1.71–1.53 (m, 6H), 1.53–1.39 (m, 6H); ^{13}C NMR (100 MHz, $CDCl_3$): δ 58.0, 29.8, 26.5, 17.0; MS (EI) *m/z*: 248 (M^+), 110 (100%); HRMS–EI (*m/z*): M^+ calcd for $C_{14}H_{24}N_4$, 248.2001; found, 248.2007.

Attempted synthesis of DAOD radical cation BArF salt (18)

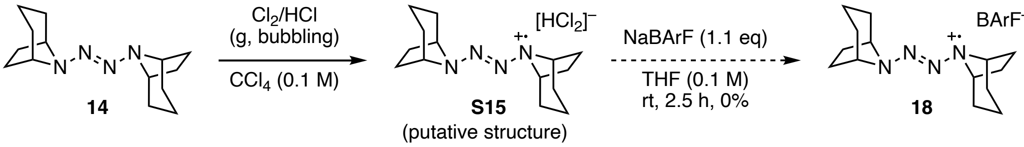

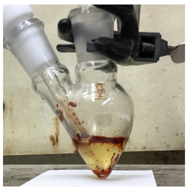

Chlorine/hydrogen chloride mixed gas, generated by adding concentrated hydrochloric acid to MnO_2 at 50 °C, was bubbled into a solution of tetrazene **14** (94.0 mg, 378 μmol) in CCl_4 (3.8 mL) at room temperature until no further precipitate formed. The resulting insoluble red solid was collected by filtration and washed several times with CCl_4. The solid on the glass filter was rinsed into a round-bottom flask with CH_2Cl_2 and concentrated under reduced pressure to give putative hydrogen dichloride salt **S15** (118 mg) as a red solid, which was used in the next reaction without further purification.

To a solution of putative hydrogen dichloride salt **S15** (118 mg) in THF (4.2 mL) was added NaBArF (405 mg, 457 μmol) at 0 °C. The mixture was stirred at room temperature for 2.5 h. However, the red color gradually faded, and BArF salt **18** could not be isolated.

Synthesis of 1,2-di(7-azabicyclo[2.2.1]heptan-7-yl)diazene (DAHD) (15)

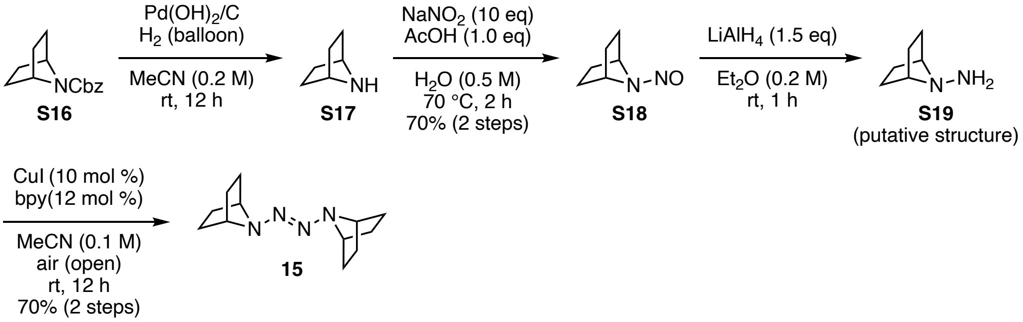

7-Nitroso-7-azabicyclo[2.2.1]heptane (S18)

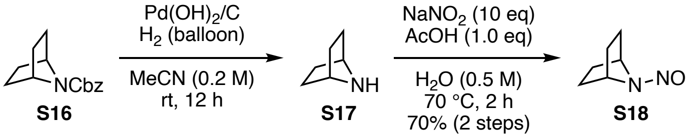

To a solution of carbamate **S16**[5] (3.78 g, 16.3 mmol) in acetonitrile (82 mL) was added $Pd(OH)_2/C$ (20% Pd, wetted with ca. 50% water, 380 mg) under an Ar atmosphere. The flask was evacuated and purged with H_2 three times, and the mixture was stirred at room temperature under a H_2 atmosphere (balloon) for 12 h. The Pd catalyst was removed by filtration through a Celite pad, and the filtrate was concentrated under reduced pressure to give 7-azabicyclo[2.2.1]heptane (**S17**) (1.61 g) as a colorless solid, which was used in the next reaction without further purification.

Amine **S17** (1.61 g) was divided into three equal portions (537 mg each). To a solution of amine **S17** (537 mg) in H_2O (11 mL) were added AcOH (0.33 mL, 5.8 mmol) and $NaNO_2$ (3.77 g, 54.7 mmol) at room temperature, and the mixture was stirred at 70 °C for 2 h. After cooling to room temperature, the three reaction mixtures were combined and extracted with Et_2O. The combined organic layers were washed with brine, dried over Na_2SO_4, and concentrated under reduced pressure. The residue was purified by column chromatography (AcOEt/*n*-hexane = 1:5 → 1:2) to give nitrosoamine **S18** (1.44 g, 11.4 mmol, 70%, 2 steps) as a yellow solid.

IR (neat, cm^{-1}): 2984, 2971, 2953, 2913, 2874, 1398, 1333, 1308, 1148, 1128, 868, 731, 712; ^{1}H NMR (400 MHz, $CDCl_3$): δ 4.98 (t, J = 4.7 Hz, 1H), 4.89 (t, J = 4.9 Hz, 1H), 2.05–1.92 (m, 2H), 1.83–1.73 (m, 2H), 1.73–1.63 (m, 2H), 1.60–1.50 (m, 2H); ^{13}C NMR (100 MHz, $CDCl_3$): δ 57.6, 51.2, 29.3, 27.3; MS (EI) *m/z*: 126 (M^+), 41 (100%); HRMS–EI (*m/z*): M^+ calcd for $C_6H_{10}N_2O$, 126.0793; found, 126.0787.

1,2-Di(7-azabicyclo[2.2.1]heptan-7-yl)diazene (DAHD) (15)

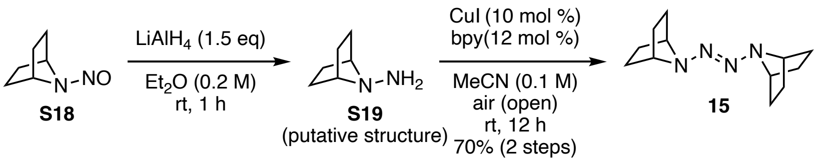

To a solution of $LiAlH_4$ (100 mg, 2.64 mmol) in Et_2O (4.0 mL) was added a solution of nitrosoamine **S18** (250 mg, 1.76 mmol) in Et_2O (5.0 mL) at 0 °C. The mixture was stirred at room temperature for 1 h, then H_2O and 10% aqueous NaOH were added slowly at 0 °C, and Et_2O was added to allow efficient stirring. After being stirred at room temperature for 30 min, the mixture was filtered through a Celite pad. The filtrate was concentrated under reduced pressure to give putative hydrazine **S19** (161 mg) as a colorless solid, which was used in the next reaction without further purification.

CuI (27.3 mg, 144 μmol) and 2,2′-bipyridine (26.9 mg, 172 μmol) were dissolved in acetonitrile (8.0 mL) open to the air. A solution of putative hydrazine **S19** (161 mg) in acetonitrile (6.0 mL) was then slowly added over 3.5 h using a syringe pump at room temperature, and the mixture was stirred for 12 h under open air. TMEDA (34 μL, 0.23 mmol) was added, and the mixture was stirred for a few minutes. The mixture was diluted with water, and extracted with CH_2Cl_2. The combined organic layers were washed with brine, dried over Na_2SO_4, and concentrated under reduced pressure. The residue was purified by column chromatography (AcOEt/*n*-hexane = 1:4) to give tetrazene **15** (135 mg, 613 μmol, 70%, 2 steps) as a colorless solid.

IR (neat, cm^{-1}): 3007, 2995, 2971, 2953, 2871, 1448, 1306, 1259, 1195, 1179, 1114, 1073, 1047, 977, 875, 807, 702; ^{1}H NMR (400 MHz, $CDCl_3$): δ 4.09 (quint, J = 2.8 Hz, 4H), 1.74–1.65 (m, 8H), 1.40–1.31 (m, 8H); ^{13}C NMR (100 MHz, $CDCl_3$): δ 58.5, 27.8; MS (EI) *m/z*: 220 (M^+), 220 (100%); HRMS–EI (*m/z*): M^+ calcd for $C_{12}H_{20}N_4$, 220.1688; found, 220.1697.

Attempted synthesis of DAHD radical cation salt

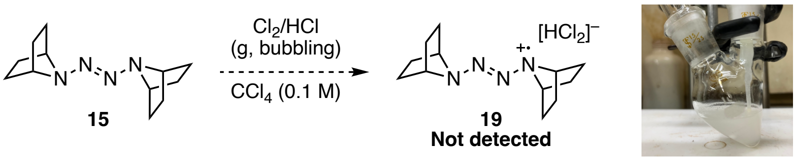

Chlorine/hydrogen chloride mixed gas, generated by adding concentrated hydrochloric acid to MnO_2 at 50 °C, was bubbled into a solution of tetrazene **15** (81.0 mg, 368 μmol) in CCl_4 (3.7 mL) at room temperature. No red solid was formed, indicating that the radical cation was not generated under these conditions.

3. X-ray Crystallography and Related Theoretical Calculations

3-1. Summary and Discussion of the Structural Characterization of 10

As described in the main text, X-ray crystallography of DAD·BArF (**10**) indicates that the distances between the four nitrogen atoms in **10** are approximately 1.31Å, suggesting that the electrons on the nitrogen atoms would be delocalized (Figures S1a and S3).

This observation was further corroborated by DFT calculations. The optimized structure of **10**, which was calculated using the crystal structure as the initial configuration, also exhibited nearly identical distances between the four nitrogen atoms (Figures S1b and S4, Table S3).

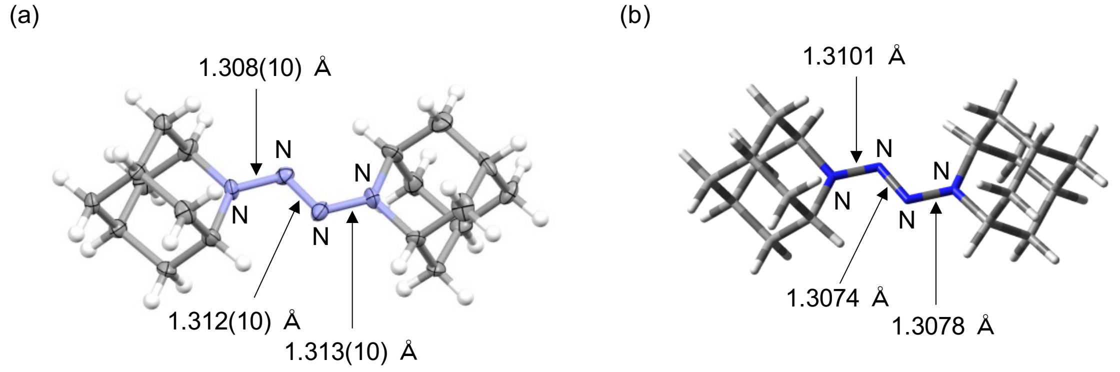

Figure S1. (a) Crystal Structure of DAD·BArF. The thermal ellipsoids are drawn with a 50% probability level and counter anions are omitted for clarity. (b) Optimized structure of DAD·BArF at the UB3LYP/6-31G(d) level of theory in the gas phase (counter anions are omitted for clarity).

On the other hand, the structure optimization calculation of neutral DAD (see Note) showed a double bond character between the two central nitrogen atoms (Figure S2a and Table S1).

Note: Structural optimization calculations were performed under zero charge and singlet spin multiplicity, using the geometry of DAD with the counter anion removed from the crystal structure.

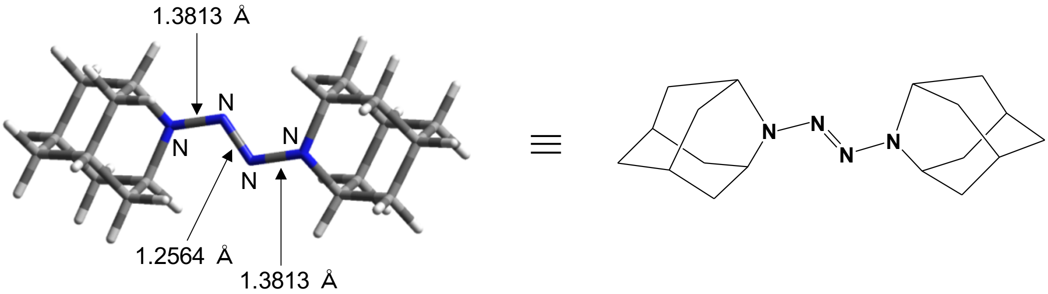

Figure S2. Optimized structure of neutral DAD at the RB3LYP/6-31G(d) level of theory in the gas phase.

To further study the bonding nature of the nitrogen in DAD, Natural Bond Orbital (NBO) charges and Wiberg Bond Indices (WBIs) analyses were performed. Table S1 presents the NBO partial charges and WBIs of the DADs. The nitrogen-nitrogen bonds in **10** and the DAD cation (Table S5) indicate their strong conjugated bond characteristics. However, the NBO charge of the neutral DAD indicated ionic bonding, and the WBI analysis showed that the central nitrogen-nitrogen had a double bond character.

Table S1. Calculated Natural bond order (NBO) partial charges and Wiberg bond indices (WBIs) for **10**, DAD radical cation and neutral DAD.

	NBO partial charges*				WBIs*		
	N^a	N^c	N^c	N^a	(N^a- N^c)	(N^c- N^c)	(N^c- N^a)
**10**	-0.117	-0.062	-0.051	-0. 114	1.3664	1.3876	1.3747
DAD cation	-0.102	-0.062	-0.062	-0.102	1.3734	1.3850	1.3734
Neutral DAD	-0.354	-0.096	-0.096	-0.354	1.1220	1.6999	1.1222

*2-azaadamantane (N^a)-central(N^c)-central(N^c)-(N^a) 2-azaadamantane

3-2. Experimental Details for X-Ray Crystallography of 10

Single crystals of DAD·BArF (**10**) suitable for X-ray crystallography were obtained by recrystallization from $CHCl_3$/*n*-hexane. A suitable crystal was selected and mounted on a Bruker D8 goniometer diffractometer. The crystal was kept at 100(1) K during data collection. Using Olex2,[6] the structure was solved with the olex2.solve[7] structure solution program using Charge Flipping and refined with the SHELXL[8] refinement package using Least Squares minimization.

Crystallographic data of **10** has been deposited with Cambridge Crystallographic Data Center, deposition no. CCDC 2325043.

Table S2. Crystal data and structure refinements for **10**.

CCDC number	2325043
Empirical formula	$C_{50}H_{40}BF_{24}N_4$
Formula weight	1163.67
Temperature/K	100(1)
Crystal system	monoclinic
Space group	Cc
a/Å	15.0246(13)
b/Å	13.8861(10)
c/Å	23.3019(19)
α/°	90
β/°	96.968(3)
γ/°	90
Volume/Å^3	4825.6(7)
Z	4
ρ_{calc}g/cm^3	1.602
μ/mm^{-1}	0.159
F(000)	2356.0
Crystal size/mm^3	0.2 × 0.13 × 0.07
Radiation	MoKα (λ = 0.71073)

2Θ range for data collection/°	4.51 to 52.772
Index ranges	$-18 \le h \le 18$, $-16 \le k \le 17$, $-29 \le l \le 28$
Reflections collected	33792
Independent reflections	9221 [R_{int} = 0.0819, R_{sigma} = 0.0762]
Data/restraints/parameters	9221/194/749
Goodness-of-fit on F^2	1.071
Final R indexes [I>=2σ (I)]	R_1 = 0.0836, wR_2 = 0.2116
Final R indexes [all data]	R_1 = 0.0905, wR_2 = 0.2172
Largest diff. peak/hole / e $Å^{-3}$	0.62/-0.61

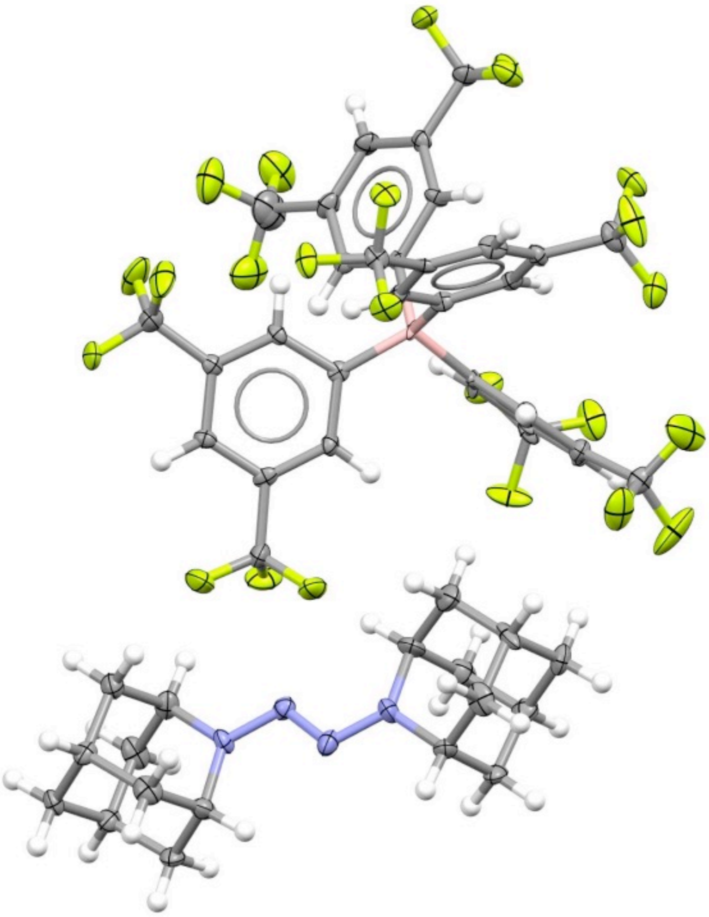

Figure S3. X-ray structure of **10**. The thermal ellipsoids are drawn with a 50% probability level. Color code of atoms: hydrogen, white; carbon, gray; fluorine, green; boron, pale pink; nitrogen, blue.

3-3. Theoretical Calculations Related to X-Ray Crystallography of 10

Density functional theory calculations were performed with Gaussian 16 program package[9] and compared with structures determined by X-ray crystallography. Vibrational mode analysis was performed for all structures to ensure that they have zero imaginary frequency.

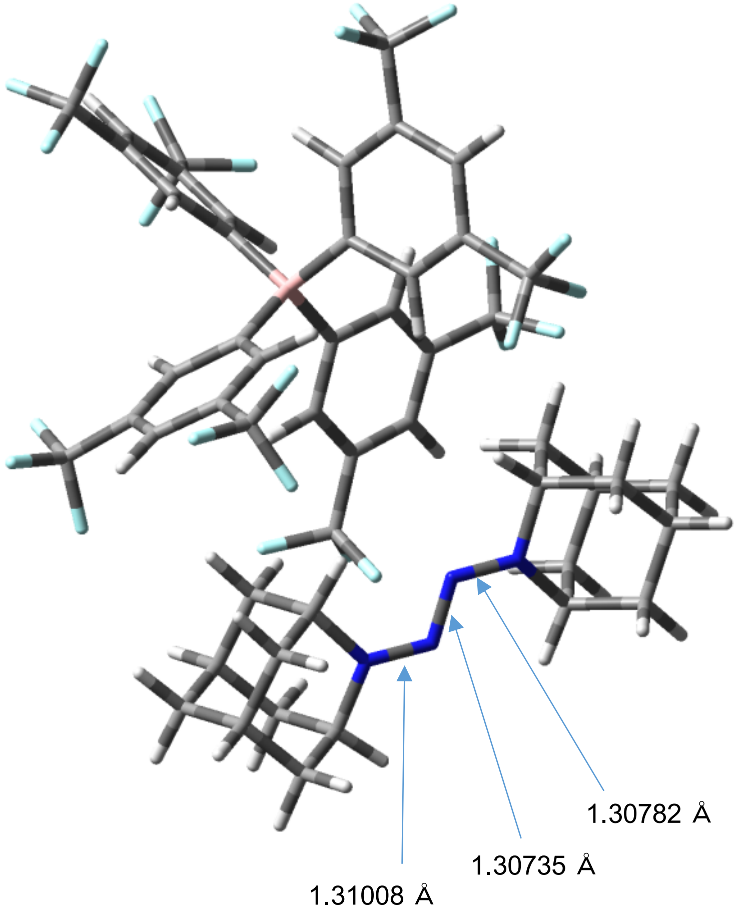

Figure S4. Optimized structure of **10** at the UB3LYP/6-31G (d) level of theory in the gas phase.

Table S3. Cartesian coordinates (Angstroms) for **10** (at the UB3LYP/6-31G (d) level of theory in gas phase).

Point Group: C1

Imaginary Freq: 0

Sum of electronic and thermal Free Energies = -4569.494 Hartree

Symbol	X	Y	Z
C	1.484336	1.506654	-0.30254
F	-0.43148	-0.0337	4.795039
F	1.721593	-0.12462	4.510845
F	0.840278	1.473297	5.702252
F	-1.71	5.664275	8.535238
F	3.570916	6.989267	-0.03558
F	-2.65218	8.158062	-1.70561
F	3.990936	5.958321	1.828893
F	-6.01025	3.944585	4.324243
F	4.251791	8.117722	1.692777
C	-1.98317	5.653512	6.179562
C	-3.34285	5.368184	6.307506
H	-3.83328	5.425203	7.272638
C	-0.62209	7.610605	2.433108
H	-1.65919	7.766259	2.719956
C	-2.51767	6.129845	0.225222
H	-1.95471	7.044306	0.378097
C	-3.40357	4.905048	3.923597
H	-3.98912	4.603092	3.061522
C	-0.17641	2.778457	3.423192
H	-0.56807	3.082355	4.388526
C	-4.17389	4.942449	-1.09306
H	-4.86945	4.903059	-1.92431
C	-3.71509	7.337157	-1.62482
B	-1.21672	5.020002	2.32918
F	2.676355	2.115467	-0.51298
C	1.072303	1.137278	2.145432
H	1.62703	0.20853	2.08853
C	-0.34308	10.09272	2.585074
F	-1.58567	10.26044	2.049335
F	-0.49362	10.32791	3.924159

F	0.429615	11.08591	2.108475
F	-4.12543	7.067498	-2.88279
C	0.871006	1.918943	1.007543
F	0.085703	5.881722	7.320171
C	0.147206	3.110925	1.086856
H	-0.00541	3.681525	0.174746
F	-4.35811	1.51175	0.082582
C	-3.98188	3.831728	-0.26941
F	0.695271	1.828395	-1.35363
F	-6.20823	5.9819	5.047142
C	3.461149	7.086932	1.310612
F	-4.72388	8.073876	-1.0517
C	-0.39631	3.586946	2.296744
F	-5.92992	4.352881	6.463039
C	-4.85903	2.629136	-0.47358
F	-1.33087	7.523343	7.473768
C	0.209084	8.728064	2.305217
F	-6.09287	2.826016	0.085808
C	-3.05673	3.875552	0.776118
H	-2.91678	2.98255	1.377843
F	1.710805	0.174023	-0.36289
F	-5.0808	2.375499	-1.78265
C	1.550342	8.574472	1.956187
H	2.203089	9.433463	1.860171
C	-5.53066	4.796998	5.256223
C	-4.0488	5.000199	5.163519
C	1.186048	6.176385	1.858218
H	1.606838	5.191316	1.685672
C	0.672759	0.725982	4.586589
C	-2.28813	5.024495	1.061569
C	-2.03348	5.171971	3.764914
C	0.539326	1.577312	3.354185

C	-0.17146	6.29721	2.202746
C	-1.22849	6.163386	7.375135
C	2.031209	7.285259	1.738429
C	-3.4396	6.095597	-0.82838
C	-1.35084	5.558741	4.936991
H	-0.29155	5.790498	4.878952
C	-9.08116	7.347908	2.562629
H	-9.49516	7.846161	3.441814
C	-6.1764	9.990364	5.461693
H	-7.23284	9.753863	5.350394
C	-7.81146	7.659251	-0.06317
H	-7.26055	7.260978	-0.92119
H	-7.90275	8.741733	-0.21498
C	-5.50846	9.133054	6.56365
H	-5.691	8.072532	6.360803
H	-5.98725	9.377156	7.520173
C	-8.92772	5.823995	2.784425
H	-9.92747	5.394122	2.922113
H	-8.35434	5.641467	3.698865
C	-8.2176	5.209416	1.56199
H	-8.09645	4.13074	1.708956
C	-6.82469	5.852695	1.426656
H	-6.27952	5.429389	0.578991
H	-6.22751	5.672304	2.324852
C	-9.90782	7.629146	1.292225
H	-10.0341	8.710584	1.15828
H	-10.9062	7.197743	1.431827
C	-5.94906	11.4934	5.726203
H	-6.43057	11.74338	6.679421
H	-6.43947	12.08957	4.946843
C	-4.04745	9.929844	4.1338
H	-3.70726	9.64725	3.137806

N	-7.72474	7.903972	2.369889
N	-5.4996	9.650845	4.182123
N	-5.9897	8.945437	3.195922
C	-3.77622	10.94156	6.885203
H	-4.19868	11.20745	7.863322
H	-2.70185	11.1582	6.932038
N	-7.24234	8.610237	3.362255
C	-3.827	11.42928	4.407937
H	-4.27801	12.02796	3.607
H	-2.74835	11.61349	4.388201
C	-4.43687	11.79004	5.776387
H	-4.28244	12.85669	5.977604
C	-6.97406	7.37348	1.199034
H	-6.00913	7.872321	1.157364
C	-3.38535	9.071958	5.236331
H	-2.30838	9.262179	5.220233
H	-3.53573	8.010046	5.01533
C	-9.19853	7.003319	0.074048
H	-9.78711	7.198502	-0.83009
C	-3.99771	9.439914	6.6015
H	-3.51726	8.836503	7.377958
C	-9.04204	5.480863	0.28534
H	-8.54364	5.028726	-0.58119
H	-10.0307	5.010054	0.366267

Table S4. Cartesian coordinates (Angstroms) for neutral DAD (at the RB3LYP/6-31G (d) level of theory in gas phase).

Point Group: C1

Imaginary Freq: 0

Sum of electronic and thermal Free Energies = -921.74369 Hartree

Symbol	X	Y	Z
C	2.551013	1.356719	0.03492

H	2.001998	2.302903	0.035832
C	-2.22156	1.021179	0.532641
H	-1.46224	1.700206	0.923126
C	2.656224	-1.44002	0.88891
H	3.103736	-2.44293	0.857633
H	1.778429	-1.4962	1.542627
C	-2.65586	1.439919	-0.88893
H	-1.7781	1.495825	-1.54275
H	-3.10305	2.442978	-0.85765
C	3.775605	1.431991	-0.89224
H	4.46891	2.196739	-0.51836
H	3.459284	1.733514	-1.89817
C	4.461531	0.051227	-0.93375
H	5.336072	0.088645	-1.5962
C	3.442822	-0.97535	-1.46782
H	3.894626	-1.97502	-1.51851
H	3.122064	-0.70595	-2.48144
C	2.992966	0.97753	1.464987
H	2.116549	0.959209	2.124957
H	3.683236	1.73682	1.857411
C	-3.44238	0.97547	1.467906
H	-3.89396	1.975235	1.518608
H	-3.12161	0.705993	2.481503
C	-2.55139	-1.35684	-0.03501
H	-2.00264	-2.30317	-0.036
N	1.628682	0.337765	-0.50866
N	-1.62877	-0.33819	0.508516
N	-0.35061	-0.52137	0.01776
C	-4.90067	0.352936	-0.49092
H	-5.3992	1.332034	-0.46626
H	-5.63484	-0.36841	-0.87626
N	0.350462	0.520632	-0.01819

C	-3.7759	-1.4318	0.892329
H	-3.45952	-1.73342	1.898212
H	-4.46952	-2.19631	0.518536
C	-4.46142	-0.05083	0.933945
H	-5.3359	-0.08802	1.596495
C	2.221939	-1.02145	-0.53269
H	1.462885	-1.70076	-0.92324
C	-2.99343	-0.97752	-1.46502
H	-3.684	-1.73656	-1.8574
H	-2.1171	-0.95946	-2.12514
C	3.672132	-0.40826	1.426805
H	3.990162	-0.69436	2.437854
C	-3.67215	0.408479	-1.42673
H	-3.99023	0.6947	-2.43773
C	4.900745	-0.35238	0.491178
H	5.399585	-1.33132	0.466613
H	5.634639	0.369207	0.876601

Table S5. Cartesian coordinates (Angstroms) for DAD radical cation (at the UB3LYP/6-31G (d) level of theory in gas phase).

Point Group: C1

Imaginary Freq: 0

Sum of electronic and thermal Free Energies = -921.54811 Hartree

Symbol	X	Y	Z
C	2.620037	1.354879	0.000284
H	2.017814	2.265837	0.000525
C	-2.28031	1.134728	0.000277
H	-1.4741	1.86562	0.00054
C	3.154327	-1.24298	1.270969
H	3.626172	-2.233	1.27307
H	2.523365	-1.1818	2.166386
C	-3.15345	1.242712	-1.27118

H	-2.52183	1.181347	-2.16612
H	-3.62528	2.232739	-1.27379
C	3.491909	1.237786	-1.26973
H	4.21058	2.065893	-1.27035
H	2.86607	1.348696	-2.16408
C	4.21539	-0.12416	-1.26533
H	4.837785	-0.20947	-2.16363
C	3.153652	-1.24276	-1.27116
H	3.6255	-2.23278	-1.27368
H	2.522117	-1.18145	-2.16616
C	3.492611	1.237562	1.269776
H	2.867296	1.348321	2.164511
H	4.211284	2.06567	1.27012
C	-3.15452	1.243025	1.270954
H	-3.62635	2.23305	1.272929
H	-2.52366	1.181876	2.166443
C	-2.62002	-1.35486	0.000435
H	-2.01783	-2.26584	0.000818
N	1.667526	0.222171	0.000433
N	-1.66752	-0.22219	0.000661
N	-0.39375	-0.52147	0.000569
C	-5.09469	0.244923	-0.00081
H	-5.62399	1.206545	-0.00116
H	-5.86302	-0.53889	-0.00103
N	0.393762	0.521451	0.00052
C	-3.4928	-1.2375	1.269804
H	-2.86762	-1.34823	2.164631
H	-4.21147	-2.06561	1.27005
C	-4.21629	0.124445	1.264615
H	-4.83928	0.209944	2.162477
C	2.28033	-1.13475	0.000195
H	1.474112	-1.86565	0.000296

C	-3.49174	-1.23783	-1.26971
H	-4.21041	-2.06594	-1.27034
H	-2.8658	-1.34879	-2.16398
C	4.216103	-0.12439	1.264741
H	4.838955	-0.20986	2.162699
C	-4.21522	0.124122	-1.26545
H	-4.83746	0.209414	-2.16386
C	5.094686	-0.2449	-0.00053
H	5.623989	-1.20652	-0.00078
H	5.863004	0.538913	-0.00069

3-4. Experimental Details for X-Ray Crystallography of 11

Single crystals of DAD·NTf_2 (**11**) suitable for X-ray crystallography were obtained by recrystallization from Et_2O. A suitable crystal was selected and mounted on a Rigaku XtaLAB Synergy with HyPix diffractometer. The crystal was kept at 150 K during data collection. Using Olex2,[6] the structure was solved with the olex2.solve[7] structure solution program using Charge Flipping and refined with the SHELXL[8] refinement package using Least Squares minimization.

Crystallographic data of **11** has been deposited with Cambridge Crystallographic Data Center, deposition no. CCDC 2493644.

Table S6. Crystal data and structure refinements for **11**.

CCDC number	2493644
Empirical formula	$C_{20}H_{28}N_5O_4F_6S_2$
Formula weight	580.59
Temperature/K	150
Crystal system	monoclinic
Space group	$P2_1/c$
a/Å	10.5267(2)
b/Å	11.9543(2)
c/Å	19.8359(4)
α/°	90
β/°	98.9616(19)
γ/°	90
Volume/Å^3	2465.68(8)
Z	4
ρ_{calc}g/cm^3	1.564
μ/mm^{-1}	2.730
F(000)	1204.0
Crystal size/mm^3	0.14 × 0.06 × 0.01
Radiation	CuKα (λ = 1.54184)

2Θ range for data collection/°	8.664 to 154.07
Index ranges	-13 ≤ h ≤ 13, -15 ≤ k ≤ 13, -24 ≤ l ≤ 25
Reflections collected	23113
Independent reflections	5037 [R_{int} = 0.0474, R_{sigma} = 0.0351]
Data/restraints/parameters	5037/0/334
Goodness-of-fit on F^2	1.067
Final R indexes [*I*>=2σ (*I*)]	R_1 = 0.0542, wR_2 = 0.1362
Final R indexes [all data]	R_1 = 0.0629, wR_2 = 0.1415
Largest diff. peak/hole / e Å^{-3}	0.46/-0.26

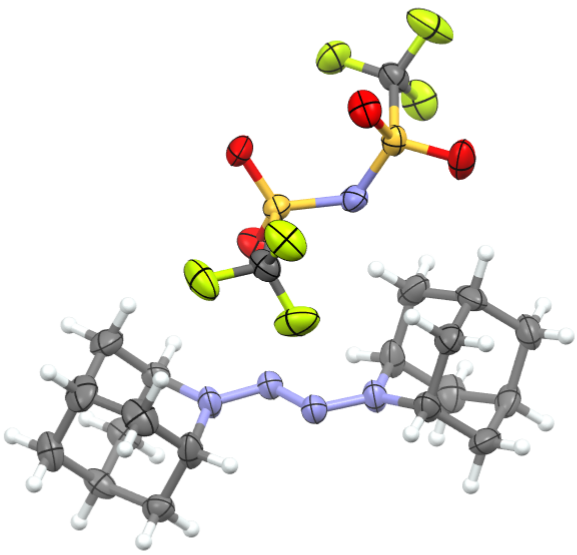

Figure S5. X-ray structure of **11**. The thermal ellipsoids are drawn with a 50% probability level. Color code of atoms: hydrogen, white; carbon, gray; fluorine, green; nitrogen, blue; oxygen, red; sulfur, yellow.

4. ESR Analysis and Related Theoretical Calculations

4-1. Experimental Details for ESR Analysis

The steady-state ESR spectra were obtained at room temperature with Bruker E580 spectrometer. Samples for ESR were prepared in dichloromethane at different concentrations and analyzed using 2 mm quartz tubes. The microwave frequencies were 9.867237 GHz for 1.27 mM, 9.860746 GHz for 0.658 mM and 9.867927 GHz for 0.146 mM solution, respectively. Field modulation strength and frequency were set as 0.03 mT and 100 kHz, respectively. The spectral simulation was performed using the included analysis software. The signal intensity (shown in Figure S6) was evaluated after integration of the ESR peaks using Igor software (version 9. 0. 5. 1).

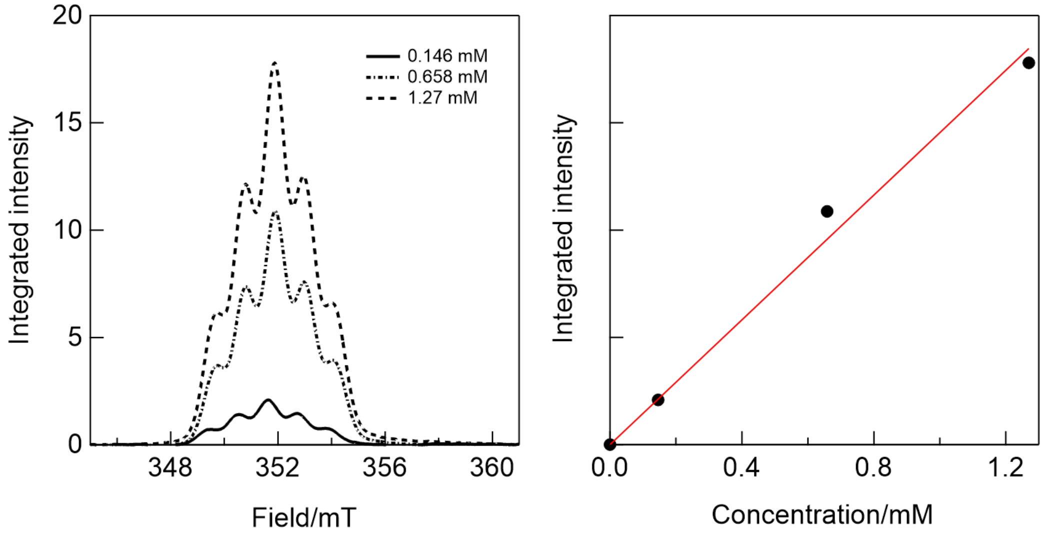

Figure S6. Concentration dependence of ESR signal intensity.

4-2. Theoretical Calculations Related to ESR Analysis

The optimization, frequency calculation, spin density analysis, and isotropic hyperfine coupling constant evaluation were performed using density functional methods with the unrestricted hybrid density functional B3LYP (UB3-LYP) functional in the Gaussian 16 program (Revision C01).[10] The triple-zeta basis function, EPR-III, was used for this purpose. The solvation effects were modeled using a polarizable continuum model (PCM) of CH_2Cl_2. The standard parameters for functional and basis set implemented in Gaussian 16 were used. The calculation was performed with Gaussian 16 installed on a PC (HP Z2 G5 mini).

Table S7. Cartesian coordinate of DAD cation radical after optimization by UB3LYP/EPR-III level of theory.

Tag	Symbol	X	Y	Z
1	C	-1.2558500	2.6555990	-0.0034990
2	H	-2.1853160	2.0928450	-0.0111710
3	C	1.2083070	2.2299600	0.0137750
4	H	1.9095940	1.4049010	0.0175260
5	C	1.3579650	3.0988230	-1.2491330
6	H	1.2789010	2.4747840	-2.1410530
7	H	2.3589420	3.5333990	-1.2402650
8	C	-1.1004070	3.5234550	-1.2652900
9	H	-1.8978120	4.2680810	-1.2674370
10	H	-1.2240270	2.9062930	-2.1571790
11	C	0.2813920	4.1951210	-1.2498120
12	H	0.3948360	4.8129150	-2.1418580
13	C	0.4220360	5.0650160	0.0126560
14	H	1.3972770	5.5575200	0.0206370
15	H	-0.3352970	5.8525320	0.0083350
16	C	-1.1188380	3.5192770	1.2632550
17	H	-1.2553580	2.8991790	2.1512050
18	H	-1.9162410	4.2638910	1.2563380
19	C	1.3395490	3.0947750	1.2814830
20	H	1.2478460	2.4679550	2.1702360

21	H	2.3404570	3.5294800	1.2883510
22	C	0.2629800	4.1910250	1.2700970
23	H	0.3633570	4.8059000	2.1657260
24	N	-0.1635410	1.6661060	0.0027460
25	N	-0.5026660	0.4111840	-0.0026830
26	N	0.5026660	-0.4111830	0.0026840
27	C	1.2558500	-2.6555990	0.0035010
28	C	-1.2083070	-2.2299600	-0.0137760
29	H	2.1853160	-2.0928450	0.0111740
30	C	1.1188390	-3.5192770	-1.2632540
31	C	1.1004060	-3.5234550	1.2652920
32	H	-1.9095940	-1.4049010	-0.0175290
33	C	-1.3395470	-3.0947750	-1.2814850
34	C	-1.3579670	-3.0988230	1.2491310
35	H	1.9162430	-4.2638910	-1.2563360
36	H	1.2553600	-2.8991790	-2.1512040
37	C	-0.2629780	-4.1910250	-1.2700970
38	H	1.2240240	-2.9062930	2.1571810
39	H	1.8978110	-4.2680800	1.2674400
40	C	-0.2813940	-4.1951210	1.2498120
41	H	-1.2478430	-2.4679550	-2.1702370
42	H	-2.3404560	-3.5294810	-1.2883540
43	H	-1.2789040	-2.4747830	2.1410510
44	H	-2.3589440	-3.5333990	1.2402630
45	H	-0.3633540	-4.8059000	-2.1657260
46	C	-0.4220360	-5.0650160	-0.0126560
47	H	-0.3948390	-4.8129150	2.1418580
48	H	-1.3972770	-5.5575200	-0.0206380
49	H	0.3352970	-5.8525320	-0.0083340
50	N	0.1635410	-1.6661050	-0.0027450

Table S8. Isotropic Fermi contact coupling terms calculated by UB3LYP/EPR-III level of theory.

	Atom	a.u.	MegaHertz	Gauss	10(-4) cm-1
1	C(13)	-0.01103	-12.40238	-4.42548	-4.13699
2	H(1)	0.00022	0.98424	0.35120	0.32831
3	C(13)	-0.00970	-10.90339	-3.89060	-3.63698
4	H(1)	0.00020	0.88025	0.31409	0.29362
5	C(13)	0.00982	11.03685	3.93822	3.68150
6	H(1)	-0.00041	-1.81306	-0.64695	-0.60477
7	H(1)	0.00100	4.48871	1.60168	1.49727
8	C(13)	0.01111	12.49377	4.45809	4.16747
9	H(1)	0.00099	4.44512	1.58613	1.48273
10	H(1)	-0.00044	-1.96879	-0.70251	-0.65672
11	C(13)	-0.00136	-1.52410	-0.54384	-0.50839
12	H(1)	0.00115	5.12703	1.82945	1.71019
13	C(13)	0.00014	0.16036	0.05722	0.05349
14	H(1)	-0.00005	-0.22273	-0.07947	-0.07429
15	H(1)	-0.00005	-0.21634	-0.07719	-0.07216
16	C(13)	0.01109	12.46937	4.44938	4.15933
17	H(1)	-0.00044	-1.96539	-0.70130	-0.65558
18	H(1)	0.00099	4.42365	1.57847	1.47557
19	C(13)	0.00980	11.01563	3.93065	3.67442
20	H(1)	-0.00041	-1.81030	-0.64596	-0.60385
21	H(1)	0.00100	4.46634	1.59370	1.48981
22	C(13)	-0.00135	-1.52198	-0.54308	-0.50768
23	H(1)	0.00115	5.11817	1.82629	1.70724
24	N(14)	0.07807	25.22392	9.00052	8.41379
25	N(14)	-0.00003	-0.01050	-0.00375	-0.00350
26	N(14)	-0.00003	-0.01050	-0.00375	-0.00350
27	C(13)	-0.01103	-12.40238	-4.42548	-4.13699
28	C(13)	-0.00970	-10.90339	-3.89060	-3.63698
29	H(1)	0.00022	0.98424	0.35120	0.32831
30	C(13)	0.01109	12.46934	4.44937	4.15932
31	C(13)	0.01111	12.49381	4.45810	4.16748
32	H(1)	0.00020	0.88025	0.31409	0.29362

33	C(13)	0.00980	11.01560	3.93064	3.67441
34	C(13)	0.00982	11.03688	3.93823	3.68151
35	H(1)	0.00099	4.42362	1.57846	1.47556
36	H(1)	-0.00044	-1.96538	-0.70130	-0.65558
37	C(13)	-0.00135	-1.52198	-0.54308	-0.50768
38	H(1)	-0.00044	-1.96879	-0.70251	-0.65672
39	H(1)	0.00099	4.44515	1.58614	1.48274
40	C(13)	-0.00136	-1.52411	-0.54384	-0.50839
41	H(1)	-0.00040	-1.81030	-0.64596	-0.60385
42	H(1)	0.00100	4.46633	1.59370	1.48981
43	H(1)	-0.00041	-1.81307	-0.64695	-0.60477
44	H(1)	0.00100	4.48872	1.60169	1.49727
45	H(1)	0.00115	5.11814	1.82628	1.70723
46	C(13)	0.00014	0.16036	0.05722	0.05349
47	H(1)	0.00115	5.12705	1.82946	1.71020
48	H(1)	-0.00005	-0.22273	-0.07947	-0.07429
49	H(1)	-0.00005	-0.21634	-0.07719	-0.07216
50	N(14)	0.07807	25.22392	9.00052	8.41379

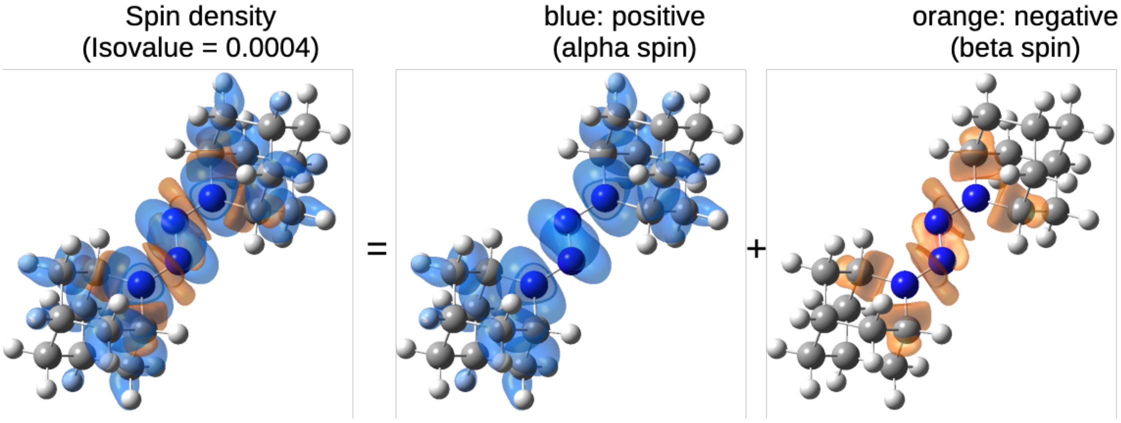

Figure S7. Spin density of the DAD cation radical calculated by UB3LYP/EPR-III level of theory. The alpha spin is shown in blue and the beta spin is shown in orange. Note that the nodal points of the alpha and beta spins are near the central N atoms.

5. Cyclic Voltammetry

Cyclic voltammograms were recorded on an ALS model 1202B electrochemical analyzer (BAS Inc.) using a conventional three-electrode configuration at room temperature (~25 °C). The working, counter, and reference electrodes were a glassy carbon disk (3 mm in diameter, BAS Inc.), platinum wire, and Ag/Ag^+ (10 mM $AgNO_3$ and 100 mM $TBAClO_4$ in MeCN, BAS Inc., RE-7), respectively. Potentials were calibrated against the ferrocene/ferrocenium (Fc/Fc^+) redox couple. The glassy carbon electrode was polished with 1 µm alumina powder (Struers K.K.) and rinsed with deionized water prior to use. Solutions of each 2-tetrazene compound (1 mM) in acetonitrile containing 100 mM $TBAPF_6$ as the supporting electrolyte were used throughout the measurements. EtOH (100 mM) and 2,4,6-collidine (10 mM) were added as substrate and base, respectively. The initial and switching potentials were –0.2 and +1.4 V vs. Ag/Ag^+, and the initial scan direction was positive, with a scan rate of 10 mV s^{-1} (Figure 7) or 100 mV s^{-1} (Figures S8–S12). Each measurement was run for two cycles, and the first cycle was plotted.

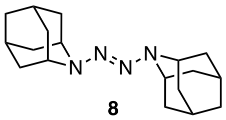

8

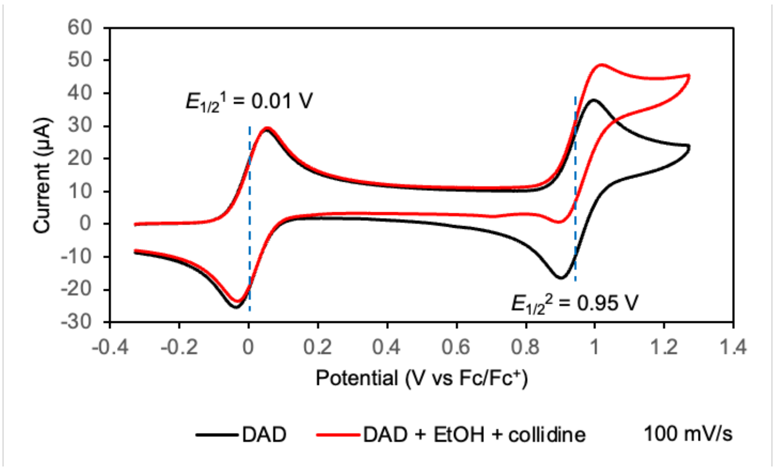

Figure S8. Cyclic voltammograms of DAD.

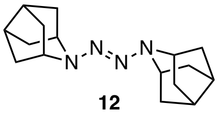

12

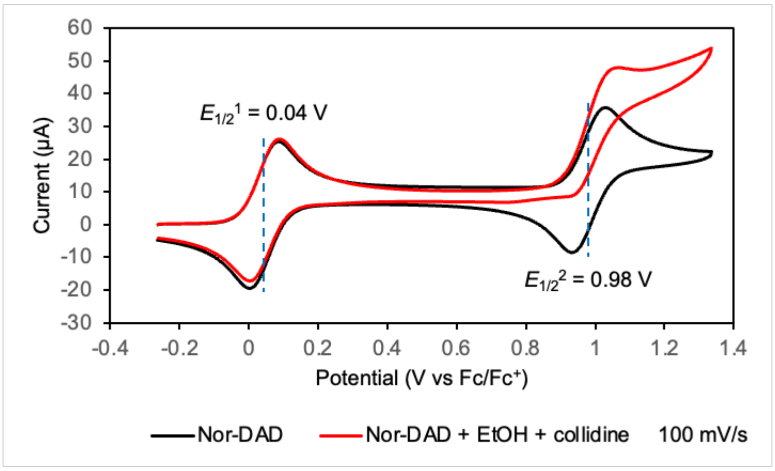

Figure S9. Cyclic voltammograms of Nor-DAD.

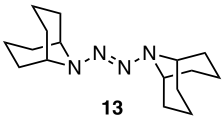

13

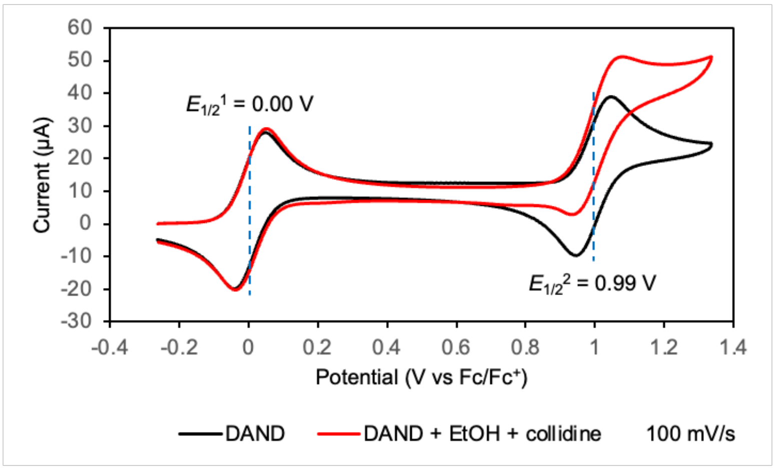

Figure S10. Cyclic voltammograms of DAND.

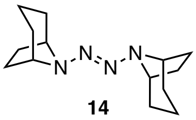

14

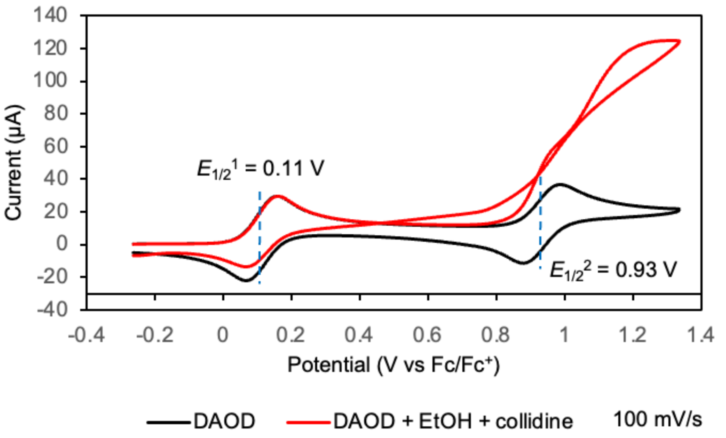

Figure S11. Cyclic voltammograms of DAOD.

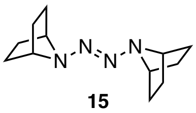

15

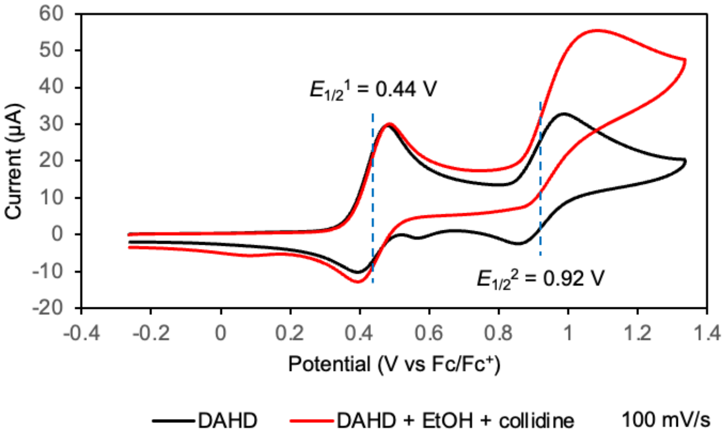

Figure S12. Cyclic voltammograms of DAHD.

6. Preparative-Scale Electrochemical Oxidation

6-1. Setup for Electrochemical Oxidation

Constant-current electrolysis was performed using a galvanostat (ECstat-302, EC Frontier) in a batch-type undivided cell (VB2, EC Frontier). Stirring was carried out with a cross-shaped, PTFE-coated magnetic stir bar (height: 7 mm; diameter: 19 mm). Carbon felt (thickness 2 mm) was cut into pieces of 11 mm × 22 mm, fixed with a Pt wire (φ = 0.5 mm), and used as the working electrode. A Pt spiral wire (CE-200, EC Frontier) was used as the counter electrode. Images of the electrolysis setup are shown in Figures S13 and S14.

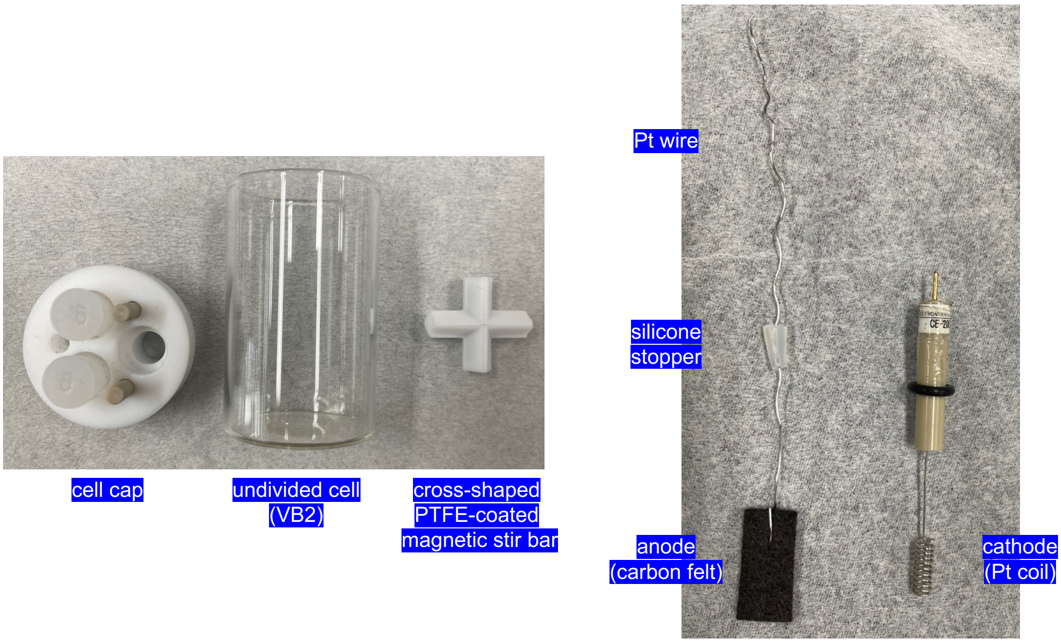

Figure S13. Photographs of individual components of the electrolysis setup.

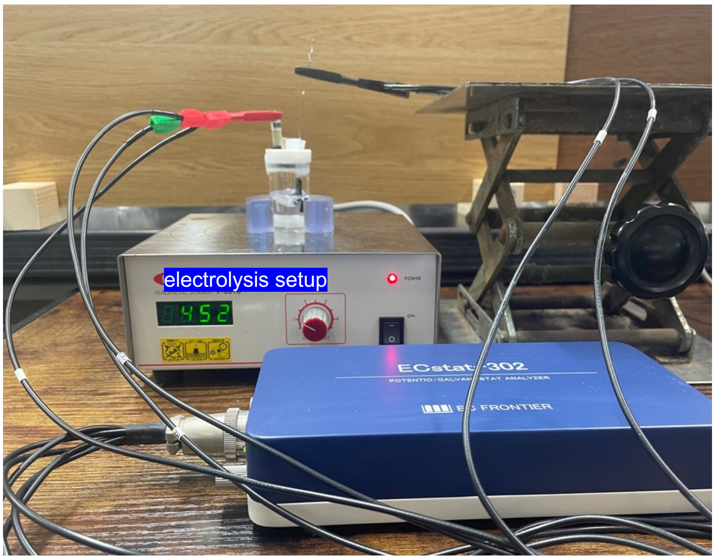

Figure S14. Photograph of the overall electrolysis setup.

6-2. Procedure for the Electrochemical Oxidation of Menthol

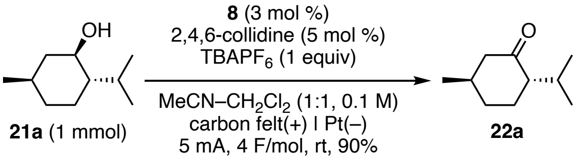

An undivided cell was charged with a solution of $TBAPF_6$ (387 mg, 1.00 mmol), menthol (**21a**, 156 mg, 1.00 mmol), DAD (**8**, 9.01 mg, 30.0 μmol), and 2,4,6-collidine (6.6 μL, 50 μmol) in $MeCN/CH_2Cl_2$ (1:1, 10 mL). A constant current (5.0 mA, 4.0 F mol^{-1}) was applied at room temperature with stirring at 450 rpm. After completion of electrolysis, the reaction mixture was diluted with water and extracted with CH_2Cl_2 (3 ×). The combined organic layers were washed with brine, dried over Na_2SO_4, and concentrated under reduced pressure. The residue was purified by column chromatography (Et_2O/*n*-hexane = 1:20) to afford menthone (**22a**, 140 mg, 904 μmol, 90%) as a colorless oil.

7. Availability of Alcohol Substrates

The following alcohols were purchased from commercial suppliers: *l*-menthol (**21a**), 2-adamantanol (**21b**), 2,2-dimethyl-3-octanol (**21c**), 4-nitrobenzylalcohol (**21h**) and 4-phenyl-1-butanol (**21i**). The following alcohols were prepared according to previously reported procedures: 1-((*tert*-butyldimethylsilyl)oxy)-2,2,4-trimethylpentan-3-ol (**21d**),[5] *N*-benzyloxycarbonyl-4-piperidinol (**21g**).[11]

1,2:4,5-Di-*O*-isopropylidene-β-D-fructopyranose (21e)[12]

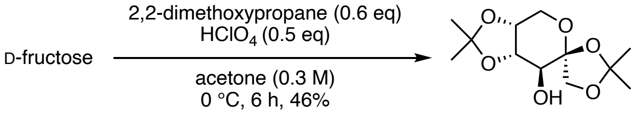

21e

To a suspension of D-fructose (92.1 g, 0.511 mol) in a mixture of acetone (1.85 L) and 2,2-dimethoxypropane (37 mL) was added 70% perchloric acid (21.5 mL, 0.249 mol) at 0 °C, and the mixture was stirred for 6 h. Concentrated aqueous ammonia (24 mL) was then added, and the volatile components (mainly acetone) were removed under reduced pressure. The residue was dissolved in CH_2Cl_2 (1.0 L), and the solution was washed with brine, dried over $MgSO_4$, and concentrated under reduced pressure. The residue was recrystallized from CH_2Cl_2/*n*-hexane to give acetal-protected fructose **21e** (61.9 g, 0.238 mol, 46%) as a colorless solid.

^{1}H NMR (400 MHz, $CDCl_3$): δ 4.26–4.07 (m, 4H), 4.06–3.94 (m, 2H), 3.67 (t, *J* = 7.5 Hz, 1H), 1.95 (d, *J* = 8.3 Hz, 1H), 1.54 (s, 3H), 1.52 (s, 3H), 1.45 (s, 3H), 1.37 (s, 3H). The ^{1}H NMR spectrum was consistent with the reported data.[13]

Trans-2-hydroxycyclohexyl benzoate (21f)[14]

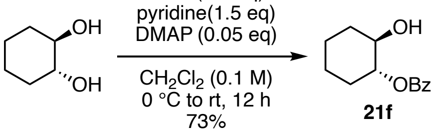

21f

To a solution of *trans*-1,2-cyclohexanediol (375 mg, 3.23 mmol) in CH_2Cl_2 (32 mL) were added pyridine (390 µL, 4.84 mmol), DMAP (19.6 mg, 161 µmol), and benzoyl chloride (450 µL, 3.87 mmol) at 0 °C. The mixture was warmed to room temperature and stirred for 12 h. The reaction was quenched with 1.0 M hydrochloric acid, and the mixture was extracted with CH_2Cl_2. The combined organic layers were washed with brine, dried

over Na_2SO_4, and concentrated under reduced pressure. The residue was purified by silica gel column chromatography (AcOEt/*n*-hexane = 1:5 → 1:3) to give ester **21f** (517 mg, 2.35 mmol, 73%) as a colorless solid.

IR (neat, cm^{-1}): 3454, 2939, 2862, 1714, 1602, 1451, 1319, 1275, 1114, 1071, 1027, 999, 712; ^1H NMR (400 MHz, $CDCl_3$): δ 8.09–8.03 (m, 2H), 7.57 (tt, *J* = 7.6, 1.5 Hz, 1H), 7.45 (t, *J* = 7.6 Hz, 2H), 4.85 (ddd, *J* = 10.4, 8.8, 4.6 Hz, 1H), 3.74 (ddd, *J* = 10.6, 8.8, 4.6 Hz, 1H), 2.22–2.07 (m, 2H), 1.82–1.72 (m, 2H), 1.52–1.27 (m, 4H); ^{13}C NMR (100 MHz, $CDCl_3$): δ 166.6, 132.9, 130.2, 129.5, 128.2, 78.4, 72.4, 32.8, 29.8, 23.7, 23.6; MS (EI) *m/z*: 221 ($[M+H]^+$), 105 (100%); HRMS–EI (*m/z*): $[M+H]^+$ calcd for $C_{13}H_{17}O_3$, 221.1178; found, 221.1173.

8. General Procedures for DAD-catalyzed Alcohol Oxidation

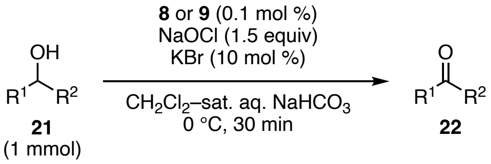

A 30 mL test tube equipped with a magnetic stirring bar was charged with a solution of alcohol **21** (1.00 mmol), DAD (**8**, 0.30 mg, 1.0 μmol) or DAD·HCl_2 (**9**, 0.37 mg, ca. 1 μmol), and KBr (11.9 mg, 0.100 mmol) in CH_2Cl_2 (2.5 mL) and saturated aqueous $NaHCO_3$ (1.25 mL). The mixture was cooled in an ice–water bath (0 °C) and stirred at 1400 rpm, and a pre-mixed solution of aqueous NaOCl (1.0 mL, 1.5 mmol, 1.54 M, purchased from Nacalai Tesque, Inc. and titrated) and saturated aqueous $NaHCO_3$ (1.25 mL) was added dropwise over 5 min. The reaction mixture was stirred at 0 °C for 30 min and then quenched with *i*-PrOH (0.58 mL). The organic layer was separated, and the aqueous layer was extracted with CH_2Cl_2. The combined organic layers were washed with brine, dried over Na_2SO_4, and concentrated under reduced pressure. The residue was purified by column chromatography to afford the corresponding carbonyl compound **22** in 87–99% yield.

Note on catalyst addition: Because of the very small quantities used, the catalysts were not weighed directly. Instead, stock solutions in CH_2Cl_2 were prepared and aliquots were added: for DAD (**8**), a 1.0 mg/mL solution (300 μL) was used; for DAD·HCl_2 (**9**), a 3.7 mg/mL solution (100 μL) was used.

9. Purification Methods and Spectral Data of the Carbonyl Products

Carbonyl products **22a**, **22b**, **22c**, **22d**, **22e**, and **22i** are known compounds. For each compound, the identity was confirmed by comparison of its ^{1}H NMR spectrum with the reported data, as indicated in the references given with the compound name.

***l*-Menthone (22a)**[13]

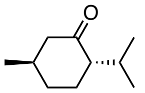

Purified by column chromatography (Et_2O/*n*-hexane = 1:20) to afford ketone **22a** as a colorless oil.

Catalyst **8**: **22a** (148 mg, 959 µmol, 96% from 1.00 mmol **21a**)
Catalyst **9**: **22a** (141 mg, 914 µmol, 92% from 998 µmol **21a**)
^{1}H NMR (400 MHz, $CDCl_3$): δ 2.36 (d, J = 13.0 Hz, 1H), 2.22–1.77 (m, 6H), 1.48–1.23 (m, 2H), 1.01 (d, J = 6.2 Hz, 3H), 0.91 (d, J = 6.8 Hz, 3H), 0.85 (d, J = 6.8 Hz, 3H).

2-Adamantanone (22b)[13]

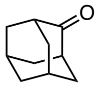

Purified by column chromatography (Et_2O/*n*-hexane = 1:10) to afford ketone **22b** as a colorless solid.

Catalyst **8**: **22b** (132 mg, 879 µmol, 88% from 998 µmol **21b**)
Catalyst **9**: **22b** (137 mg, 912 µmol, 91% from 1.00 mmol **21b**)
^{1}H NMR (400 MHz, $CDCl_3$): δ 2.55 (s, 2H), 2.13–1.91 (m, 12H).

2,2-Dimethyl-octan-3-one (22c)[13]

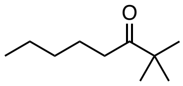

Purified by column chromatography (Et_2O/*n*-hexane = 1:20) to afford ketone **22d** as a colorless oil.

Catalyst **8**: **22c** (144 mg, 921 µmol, 92% from 1.00 mmol **21c**)
Catalyst **9**: **22c** (147 mg, 941 µmol, 94% from 1.00 mmol **21c**)
^{1}H NMR (400 MHz, $CDCl_3$): δ 2.47 (t, J = 7.4 Hz, 2H), 1.55 (quint, J = 7.4 Hz, 2H),

1.36–1.21 (m, 4H), 1.13 (s, 9H), 0.89 (t, J = 7.1 Hz, 3H).

1-(*Tert*-butyldimethylsilyloxy)-2,2,4-trimethylpentan-3-one (22d)[5]

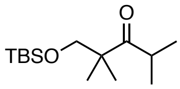

Purified by column chromatography (Et_2O/*n*-hexane = 1:100) to afford ketone **22e** as a colorless oil.

Catalyst **8**: **22d** (234 mg, 905 μmol, 91% from 998 μmol **21d**)

Catalyst **9**: **22d** (236 mg, 913 μmol, 91% from 1.00 mmol **21d**)

^{1}H NMR (400 MHz, $CDCl_3$): δ 3.60 (s, 2H), 3.11 (sept, J = 6.7 Hz, 1H), 1.11 (s, 6H), 1.03 (d, J = 6.7 Hz, 6H), 0.87 (s, 9H), 0.03 (s, 6H).

1,2:4,5-Di-*O*-isopropylidene-β-D-fructopyranose-3-one (22e)[13]

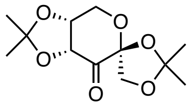

Purified by column chromatography (AcOEt /*n*-hexane = 1: 5) to afford ketone **22e** as a colorless solid.

Catalyst **8**: **22e** (253 mg, 978 μmol, 98% from 1.00 mmol **21e**)

Catalyst **9**: **22e** (257 mg, 995 μmol, 99% from 1.00 mmol **21e**)

^{1}H NMR (400 MHz, $CDCl_3$): δ 4.73 (d, J = 5.6 Hz, 1H), 4.62 (d, J = 9.5 Hz, 1H), 4.57–4.53 (m, 1H), 4.39 (dd, J = 13.5, 2.1 Hz, 1H), 4.13 (d, J = 13.5 Hz, 1H), 4.00 (d, J = 9.5 Hz, 1H), 1.55 (s, 3H), 1.47 (s, 3H), 1.40 (s, 6H).

2-Oxocyclohexyl benzoate (22f)

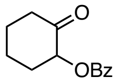

Purified by column chromatography (AcOEt/*n*-hexane = 1:10) to afford ketone **22f** as a colorless solid.

Catalyst **8**: **22f** (206 mg, 944 μmol, 94% from 1.00 mmol **21f**)

Catalyst **9**: **22f** (216 mg, 990 μmol, 99% from 1.00 mmol **21f**)

IR (neat, cm^{-1}): 2945, 2868, 1719, 1603, 1451, 1316, 1301, 1273, 1217, 1176, 1113, 1071, 1035, 884, 712; ^{1}H NMR (400 MHz, $CDCl_3$): δ 8.10 (d, J = 7.4 Hz, 2H), 7.57 (t, J = 7.0 Hz, 1H), 7.45 (t, J = 7.4 Hz, 2H), 5.42 (dd, J = 11.7, 6.3 Hz, 1H), 2.58 (d, J = 13.6 Hz,

1H), 2.53–2.38 (m, 2H), 2.19–2.09 (m, 1H), 2.09–2.00 (m, 1H), 2.01–1.78 (m, 2H), 1.77–1.63 (m, 1H); ^{13}C NMR (100 MHz, $CDCl_3$): δ 204.3, 165.5, 133.1, 129.8, 129.6, 128.3, 40.7, 33.1, 27.1, 23.7; MS (EI) *m/z*: 218 (M^+), 105 (100%); HRMS–EI (*m/z*): M^+ calcd for $C_{13}H_{14}O_3$, 218.0943; found, 218.0944.

Benzyl 4-oxopiperidine-1-carboxylate (22g)

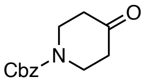

Purified by column chromatography (AcOEt/*n*-hexane = 1:2) to afford ketone **22g** as a colorless oil.

Catalyst **8**: **22g** (229 mg, 982 μmol, 98% from 1.01 mmol **21g**)

Catalyst **9**: **22g** (232 mg, 995 μmol, 99% from 1.00 mmol **21g**)

IR (neat, cm^{-1}): 3516, 2964, 2879, 1695, 1434, 1364, 1312, 1273, 1216, 1120, 1062, 992, 755, 699; ^{1}H NMR (400 MHz, $CDCl_3$): δ 7.40–7.31 (m, 5H), 5.18 (s, 2H), 3.80 (t, J = 6.3 Hz, 4H), 2.46 (brs, 4H); ^{13}C NMR (100 MHz, $CDCl_3$): δ 207.0, 154.9, 136.1, 128.4, 128.0, 127.8, 67.4, 42.8, 40.8; MS (EI) *m/z*: 233 (M^+), 91 (100%); HRMS–EI (*m/z*): M^+ calcd for $C_{13}H_{15}NO_3$, 233.1051; found, 233.1051.

4-Nitrobenzaldehyde (22h)

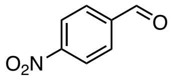

Purified by column chromatography (Et_2O/*n*-hexane = 1:20) to afford ketone **22h** as a colorless solid.

Catalyst **8**: **22h** (143 mg, 946 μmol, 95% from 1.00 mmol **21h**)

Catalyst **9**: **22h** (150 mg, 993 μmol, 99% from 1.00 mmol **21h**)

IR (neat, cm^{-1}): 3107, 2851, 1709, 1605, 1536, 1381, 1347, 1326, 1287, 1198, 1104, 1008, 850, 817, 739, 677; ^{1}H NMR (400 MHz, $CDCl_3$): δ 10.2 (s, 1H), 8.41 (d, J = 8.8 Hz, 2H), 8.09 (d, J = 8.8 Hz, 2H); ^{13}C NMR (100 MHz, $CDCl_3$): δ 190.3, 151.1, 140.0, 130.5, 124.3; MS (EI) *m/z*: 151 (M^+), 151 (100%); HRMS–EI (*m/z*): M^+ calcd for $C_7H_5NO_3$, 151.0269; found, 151.0273.

4-Phenyl-1-butanal (22i)[13]

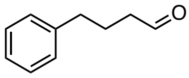

Purified by column chromatography (Et_2O/*n*-hexane = 1:20) to afford ketone **22i** as a colorless oil.

Catalyst **8**: **22i** (133 mg, 897 μmol, 90% from 1.00 mmol **21i**)

Catalyst **9**: **22i** (139 mg, 938 μmol, 91% from 1.03 mmol **21i**)

^{1}H NMR (400 MHz, $CDCl_3$): δ 9.76 (s, 1H), 7.29 (t, *J* = 7.3 Hz, 2H), 7.24–7.13 (m, 3H), 2.66 (t, *J* = 7.3 Hz, 2H), 2.45 (t, *J* = 7.3, 2H), 1.97 (quint, *J* = 7.3 Hz, 2H).

10. Recovery and Analysis of Catalyst-Derived Materials

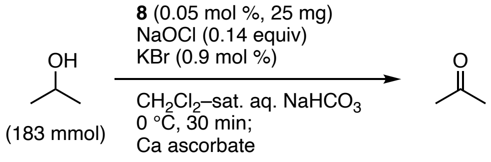

8 was recovered in 92% yield (23 mg).

A 300 mL two-necked round-bottom flask equipped with a magnetic stirring bar was charged with a solution of *i*-PrOH (14.0 mL, 183 mmol), KBr (198 mg, 1.66 mmol), and DAD (**8**, 25.0 mg, 83.2 μmol) in CH_2Cl_2 (41 mL) and saturated aqueous $NaHCO_3$ (20.5 mL). The mixture was cooled in an ice–water bath (0 °C), and a pre-mixed solution of aqueous NaOCl (16.0 mL, 24.6 mmol, 1.54 M, purchased from Nacalai Tesque, Inc., and titrated) and saturated aqueous $NaHCO_3$ (20.5 mL) was added dropwise over 10 min. The reaction mixture was stirred at 0 °C for 30 min (including the NaOCl addition time) and then quenched with calcium ascorbate dihydrate (70.7 mg, 166 μmol). Water was added until the remaining solid calcium ascorbate completely dissolved, and the mixture was stirred at room temperature for 10 min. The flask was then purged with argon by repeated evacuation and refilling cycles and the mixture was vigorously stirred. After phase separation was clearly observed, the organic layer was collected using a syringe, dried over Na_2SO_4, and concentrated under reduced pressure. The residue was purified by column chromatography (AcOEt/*n*-hexane = 1:16 → 1:8) to recover **8** (23.0 mg, 76.6 μmol, 92%) as a colorless solid.

Note: Although the exact factors responsible for the color change are not yet fully understood, it was observed that solutions containing **8** together with certain impurities (e.g., hydrogen chloride or calcium ascorbate) can gradually turn red upon exposure to air (possibly under ambient light), suggesting partial formation of the radical cation. Therefore, in this experiment, the separation of the organic and aqueous layers was carried out under an argon atmosphere to maximize the recovery of **8**.

11. Differential Scanning Calorimetry Analysis

Differential scanning calorimetry (DSC) was performed using a DSC8230 instrument (Rigaku Co., Ltd.). DAD (**8**, 3.54 mg) and α-Al_2O_3 (3.59 mg, used as a reference material) were sealed in aluminum pans and heated from 27 °C to 300 °C at a rate of 10 °C min^{-1} under a nitrogen flow of 25 mL min^{-1}.

The DSC trace exhibited a broad and gradual exothermic event with an onset at approximately 170 °C, consistent with the initiation of slow thermal decomposition (Figure S15). No sharp exothermic spike was observed, indicating the absence of rapid, self-accelerating decomposition under the measurement conditions.

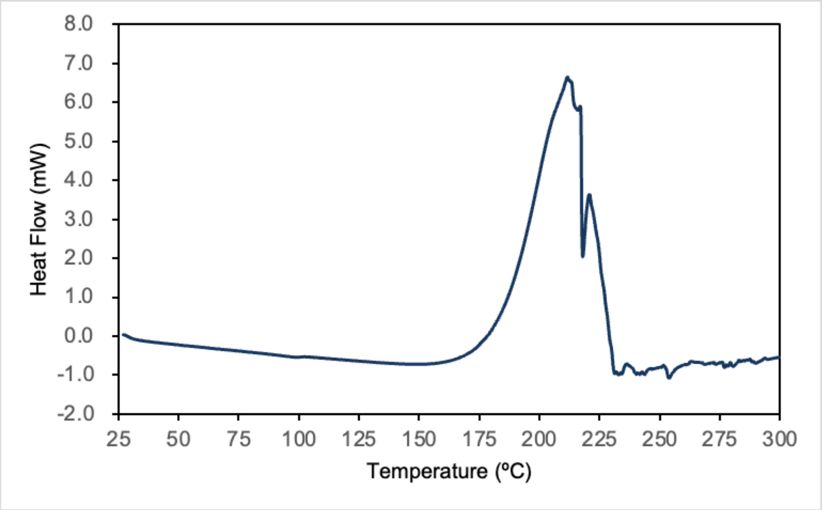

Figure S15. DSC thermogram of DAD, recorded under nitrogen at a heating rate of 10 °C min^{-1} from 27 to 300 °C.

12. References

(1) Shibuya, M.; Sasano, Y.; Tomizawa, M.; Hamada, T.; Kozawa, M.; Nagahama, N.; Iwabuchi, Y. Practical Preparation Methods for Highly Active Azaadamantane-Nitroxyl-Radical-Type Oxidation Catalysts. *Synthesis* **2011** (21), 3418-3425.

(2) Suzuki, S.; Sakai, T.; Takagi, S.; Naota, T. On-Demand Control of Short-Wave Infrared Light Transparency Based on Stimuli-Responsive Association of Tetrathiafulvalene Radical Cations. *Angew. Chem., Int. Ed.* **2023**, *62* (35), e202308570.

(3) Hayashi, M.; Sasano, Y.; Nagasawa, S.; Shibuya, M.; Iwabuchi, Y. 9-Azanoradamantane N-Oxyl (Nor-AZADO): A Highly Active Organocatalyst for Alcohol Oxidation. *Chem. Pharm. Bull.* **2011**, *59* (12), 1570-1573.

(4) Song, Z. J.; Zhou, G.; Ye, H.; Cohen, R.; Tan, L. Preparation of 9-Azabicyclo[3.3.1]nonane-N-oxyl (ABNO). *Org. Synth.* **2022**, *99*, 251-273.

(5) Toda, M.; Sasano, Y.; Takahashi, M.; Fujiki, S.; Kasabata, K.; Ono, T.; Sato, K.; Kashiwagi, Y.; Iwabuchi, Y. Identification of the Optimal Framework for Nitroxyl Radical/Hydroxylamine in Copper-Cocatalyzed Aerobic Alcohol Oxidation. *J. Org. Chem.* **2023**, *88* (3), 1434-1444.

(6) Dolomanov, O. V.; Bourhis, L. J.; Gildea, R. J.; Howard, J. A. K.; Puschmann, H. *OLEX2*: a complete structure solution, refinement and analysis program. *J. Appl. Crystallogr.* **2009**, *42*, 339-341.

(7) Bourhis, L. J.; Dolomanov, O. V.; Gildea, R. J.; Howard, J. A. K.; Puschmann, H. The anatomy of a comprehensive constrained, restrained refinement program for the modern computing environment - *Olex2* dissected. *Acta Crystallogr.* **2015**, *A71*, 59-75.

(8) Sheldrick, G. M. Crystal structure refinement with *SHELXL*. *Acta Crystallogr.* **2015**, *C71*, 3-8.

(9) Gaussian 16, Revision A.03; Frisch, M. J.; Trucks, G. W.; Schlegel, H. B.; Scuseria, G. E.; Robb, M. A.; Cheeseman, J. R.; Scalmani, G.; Barone, V.; Petersson, G. A.; Nakatsuji, H.; Li, X.; Caricato, M.; Marenich, A. V.; Bloino, J.; Janesko, B. G.; Gomperts, R.; Mennucci, B.; Hratchian, H. P.; Ortiz, J. V.; Izmaylov, A. F.; Sonnenberg, J. L.; Williams-Young, D.; Ding, F.; Lipparini, F.; Egidi, F.; Goings, J.; Peng, B.; Petrone, A.; Henderson, T.; Ranasinghe, D.; Zakrzewski, V. G.; Gao, J.;

Rega, N.; Zheng, G.; Liang, W.; Hada, M.; Ehara, M.; Toyota, K.; Fukuda, R.; Hasegawa, J.; Ishida, M.; Nakajima, T.; Honda, Y.; Kitao, O.; Nakai, H.; Vreven, T.; Throssell, K.; Montgomery, J. A., Jr.; Peralta, J. E.; Ogliaro, F.; Bearpark, M. J.; Heyd, J. J.; Brothers, E. N.; Kudin, K. N.; Staroverov, V. N.; Keith, T. A.; Kobayashi, R.; Normand, J.; Raghavachari, K.; Rendell, A. P.; Burant, J. C.; Iyengar, S. S.; Tomasi, J.; Cossi, M.; Millam, J. M.; Klene, M.; Adamo, C.; Cammi, R.; Ochterski, J. W.; Martin, R. L.; Morokuma, K.; Farkas, O.; Foresman, J. B.; Fox, D. J. Gaussian, Inc.: Wallingford, CT, 2016.

(10) Gaussian 16, Revision C.01; Frisch, M. J.; Trucks, G. W.; Schlegel, H. B.; Scuseria, G. E.; Robb, M. A.; Cheeseman, J. R.; Scalmani, G.; Barone, V.; Petersson, G. A.; Nakatsuji, H.; Li, X.; Caricato, M.; Marenich, A. V.; Bloino, J.; Janesko, B. G.; Gomperts, R.; Mennucci, B.; Hratchian, H. P.; Ortiz, J. V.; Izmaylov, A. F.; Sonnenberg, J. L.; Williams-Young, F.; Ding, F.; Lipparini, F.; Egidi, F.; Goings, J.; Peng, B.; Petrone, A.; Henderson, T.; Ranasinghe, D.; Zakrzewski, V. G.; Gao, J.; Rega, N.; Zheng, G.; Liang, W.; Hada, M.; Ehara, M.; Toyota, K.; Fukuda, R.; Hasegawa, J.; Ishida, M.; Nakajima, T.; Honda, Y.; Kitao, O.; Nakai, H.; Vreven, T.; Throssell, K.; Montgomery, J. A., Jr.; Peralta, J. E.; Ogliaro, F.; Bearpark, M. J.; Heyd, J. J.; Brothers, E. N.; Kudin, K. N.; Staroverov, V. N.; Keith, T. A.; Kobayashi, R.; Normand, J.; Raghavachari, K.; Rendell, A. P.; Burant, J. C.; Iyengar, S. S.; Tomasi, J.; Cossi, M.; Millam, J. M.; Klene, M.; Adamo, C.; Cammi, R.; Ochterski, J. W.; Martin, R. L.; Morokuma, K.; Farkas, O.; Foresman, J. B.; Fox, D. J. Gaussian, Inc.: Wallingford, CT, 2016.

(11) Sasano, Y.; Nagasawa, S.; Yamazaki, M.; Shibuya, M.; Park, J.; Iwabuchi, Y. Highly Chemoselective Aerobic Oxidation of Amino Alcohols into Amino Carbonyl Compounds. *Angew. Chem., Int. Ed.* **2014**, *53* (12), 3236-3240.

(12) Mio, S.; Kumagawa, Y.; Sugai, S. Synthetic Studies on (+)-Hydantocidin (3): a New Synthetic Method for Construction of the Spiro-Hydantoin Ring at the Anomeric Position of D-Ribofuranose. *Tetrahedron* **1991**, *47* (12-13), 2133-2144.

(13) Sasano, Y.; Murakami, K.; Nishiyama, T.; Kwon, E.; Iwabuchi, Y. 3-Methyl-4-oxa-5-azahomoadamantane: Alkoxyamine-Type Organocatalyst for Alcohol Oxidation. *Angew. Chem., Int. Ed.* **2013**, *52* (48), 12624-12627.

(14) Ochiai, H.; Niwa, T.; Hosoya, T. Stereoinversion of Stereocongested Carbocyclic Alcohols via Triflylation and Subsequent Treatment with Aqueous N,N-

Dimethylformamide. *Org. Lett.* **2016**, *18* (23), 5982-5985.

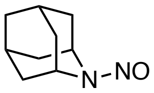

6
(400 MHz, $CDCl_3$)

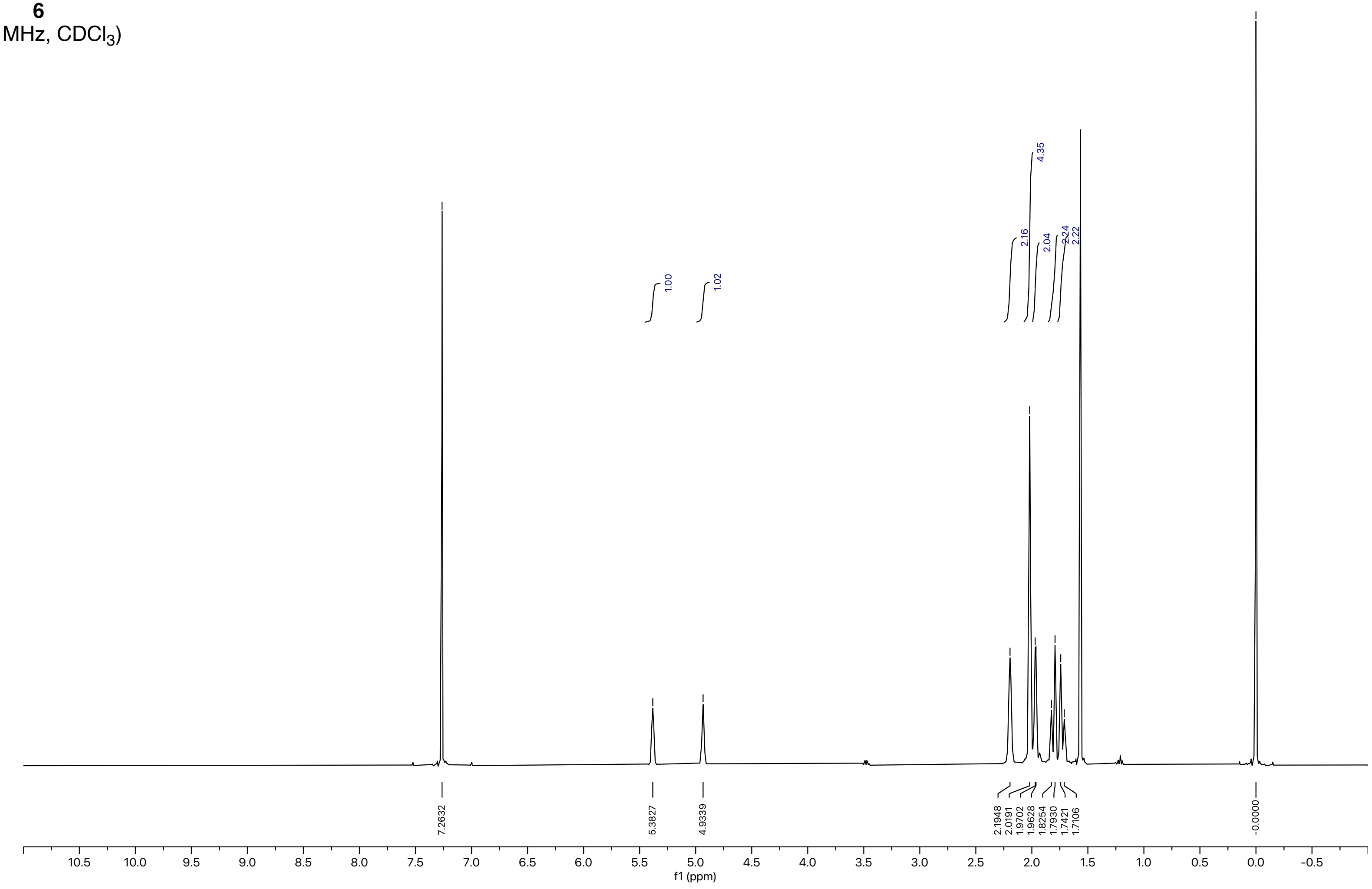

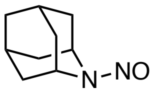

6

(100 MHz, $CDCl_3$)

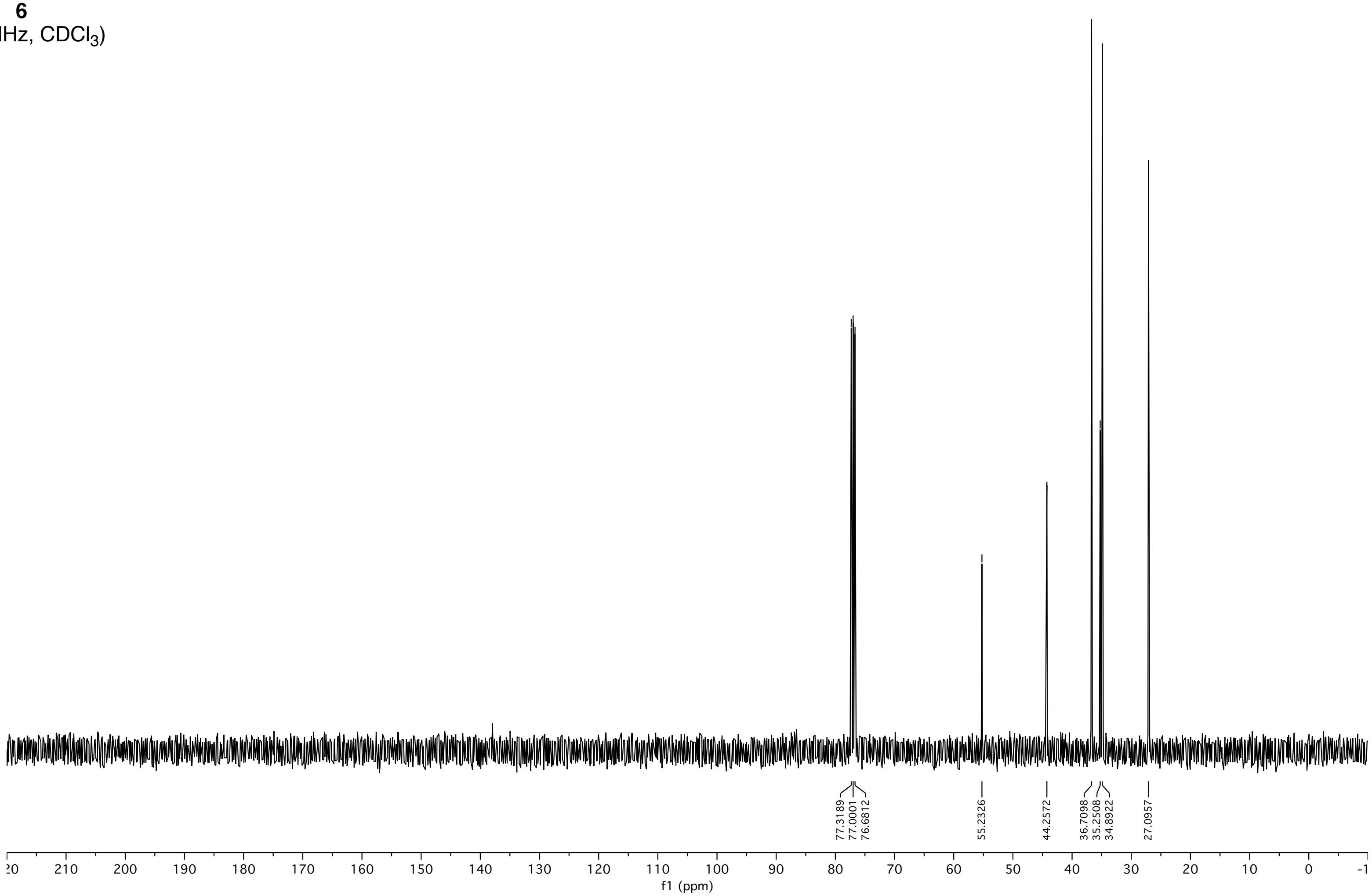

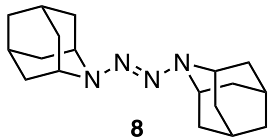

8
(400 MHz, $CDCl_3$)

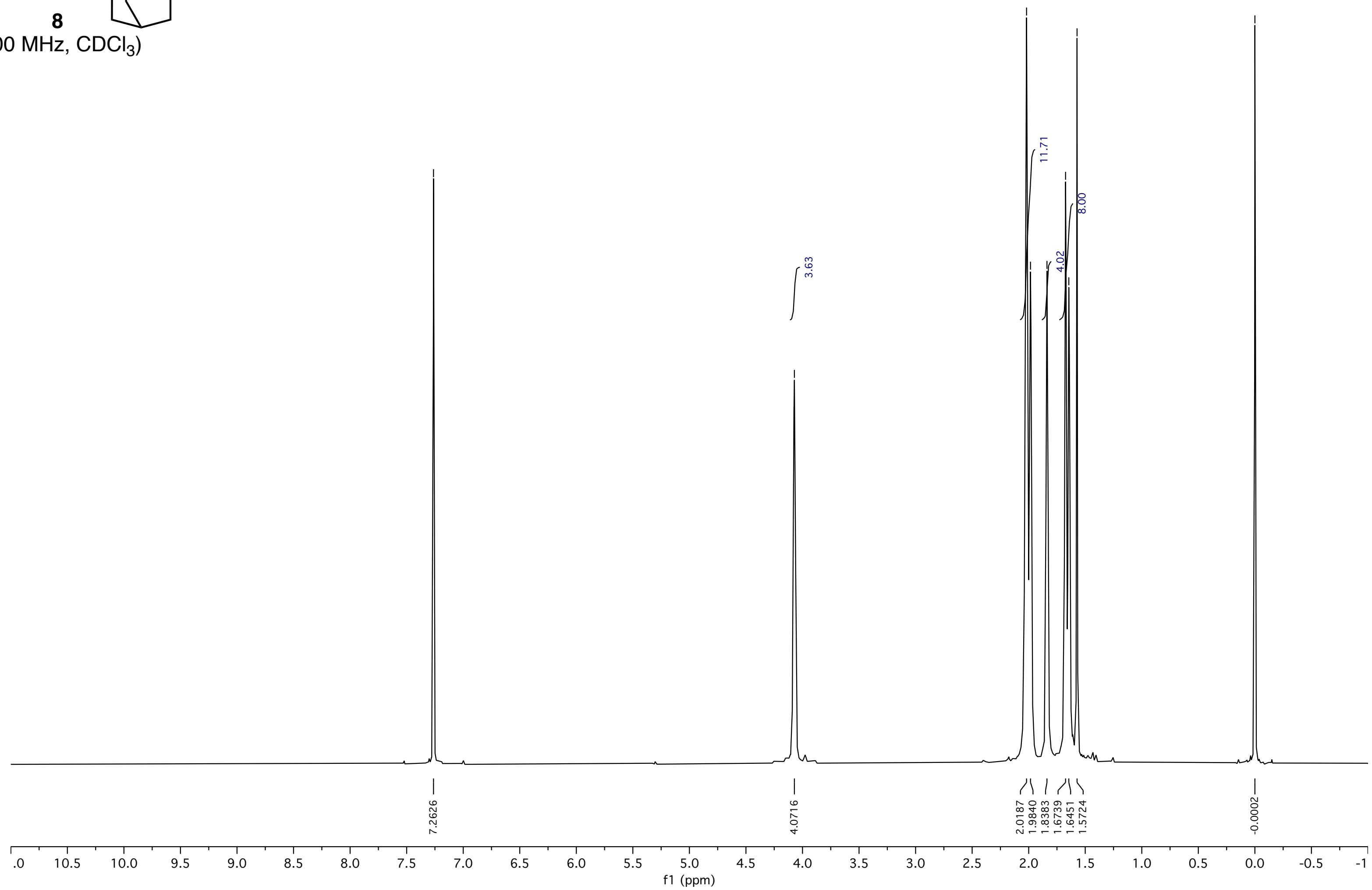

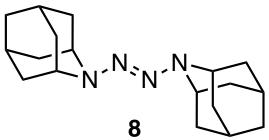

8
(100 MHz, $CDCl_3$)

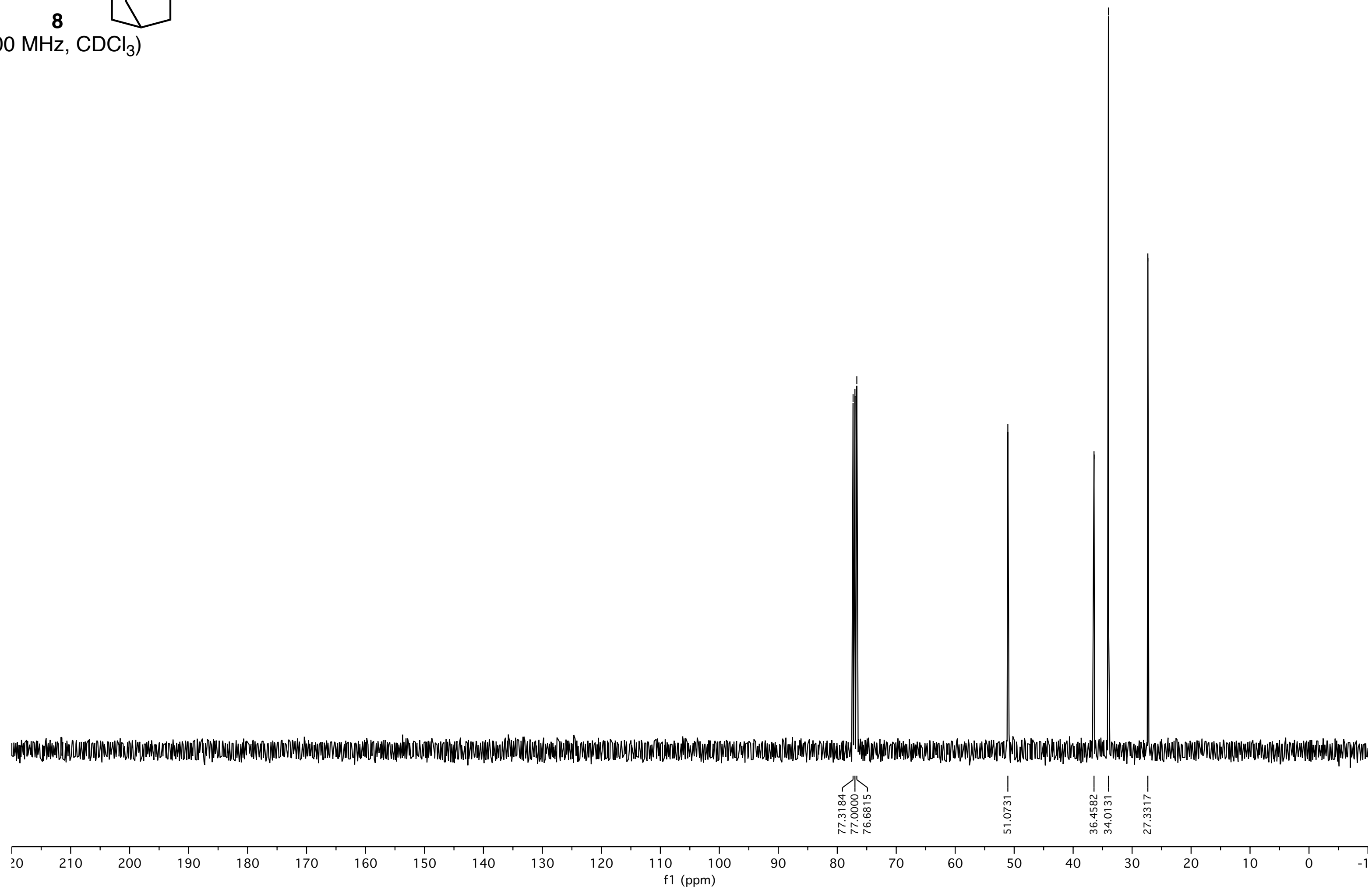

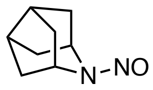
S3
(400 MHz, $CDCl_3$)

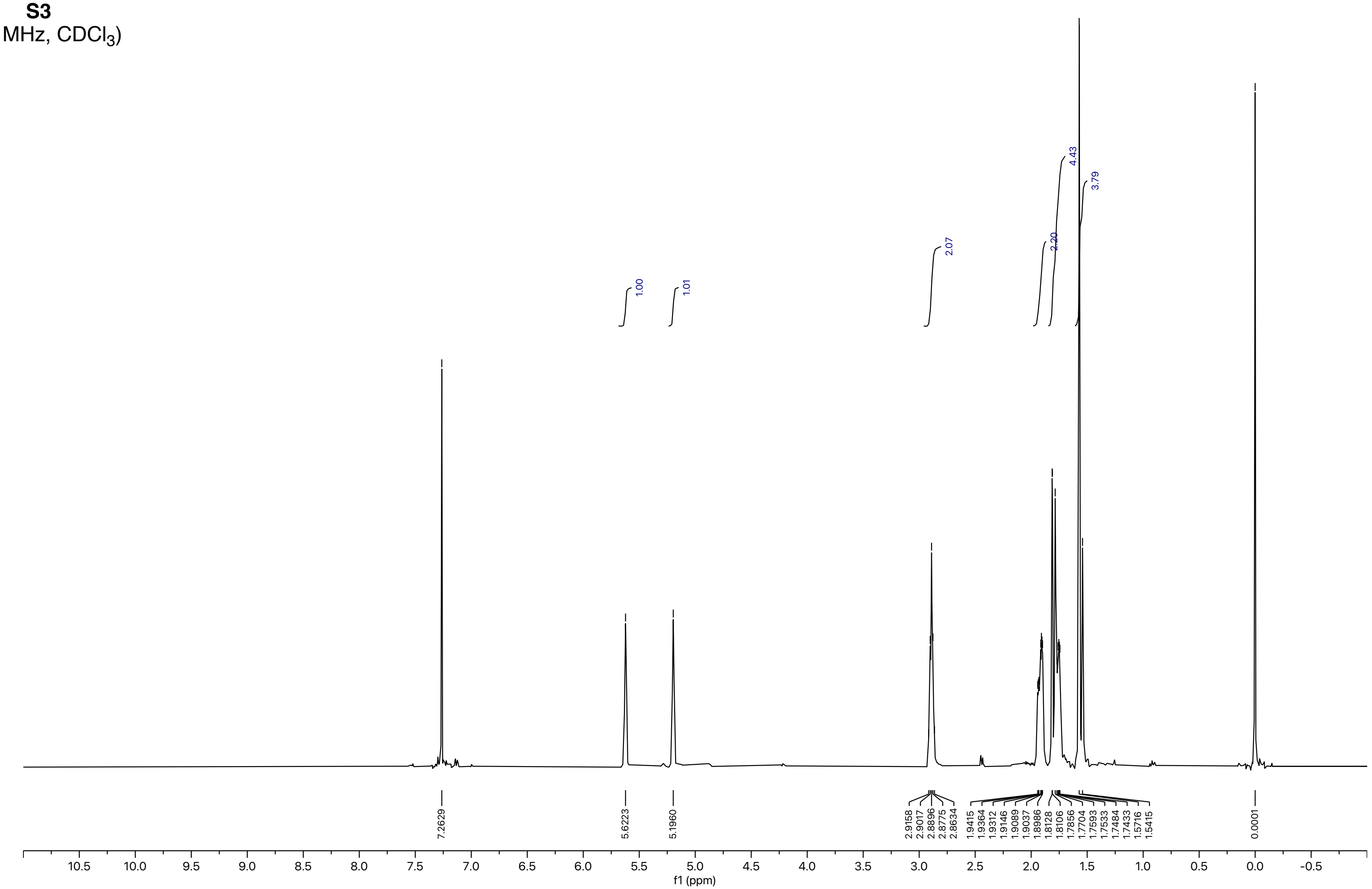

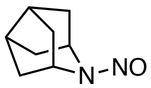

S3
(100 MHz, $CDCl_3$)

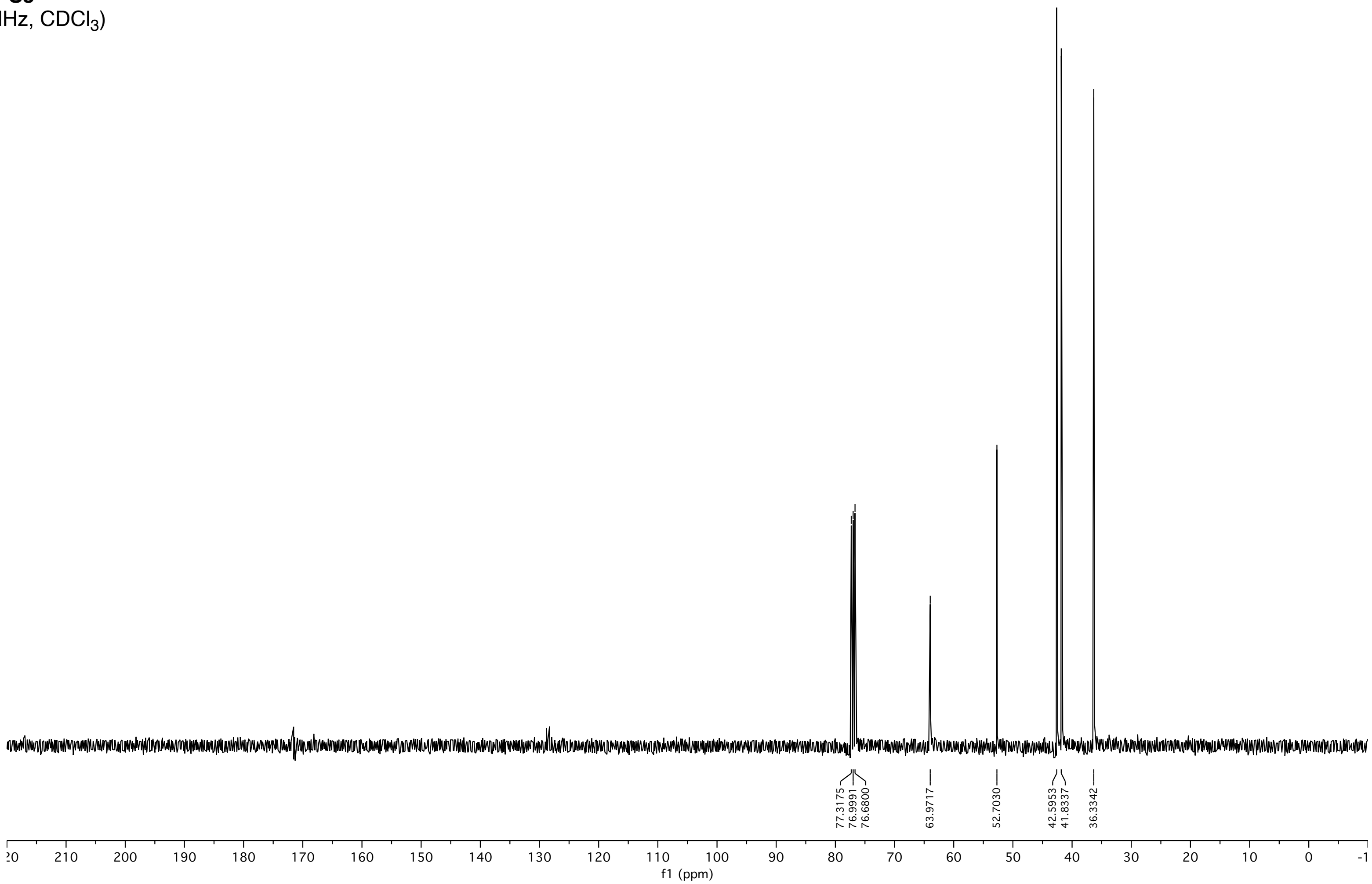

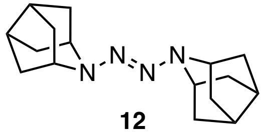

12
(400 MHz, $CDCl_3$)

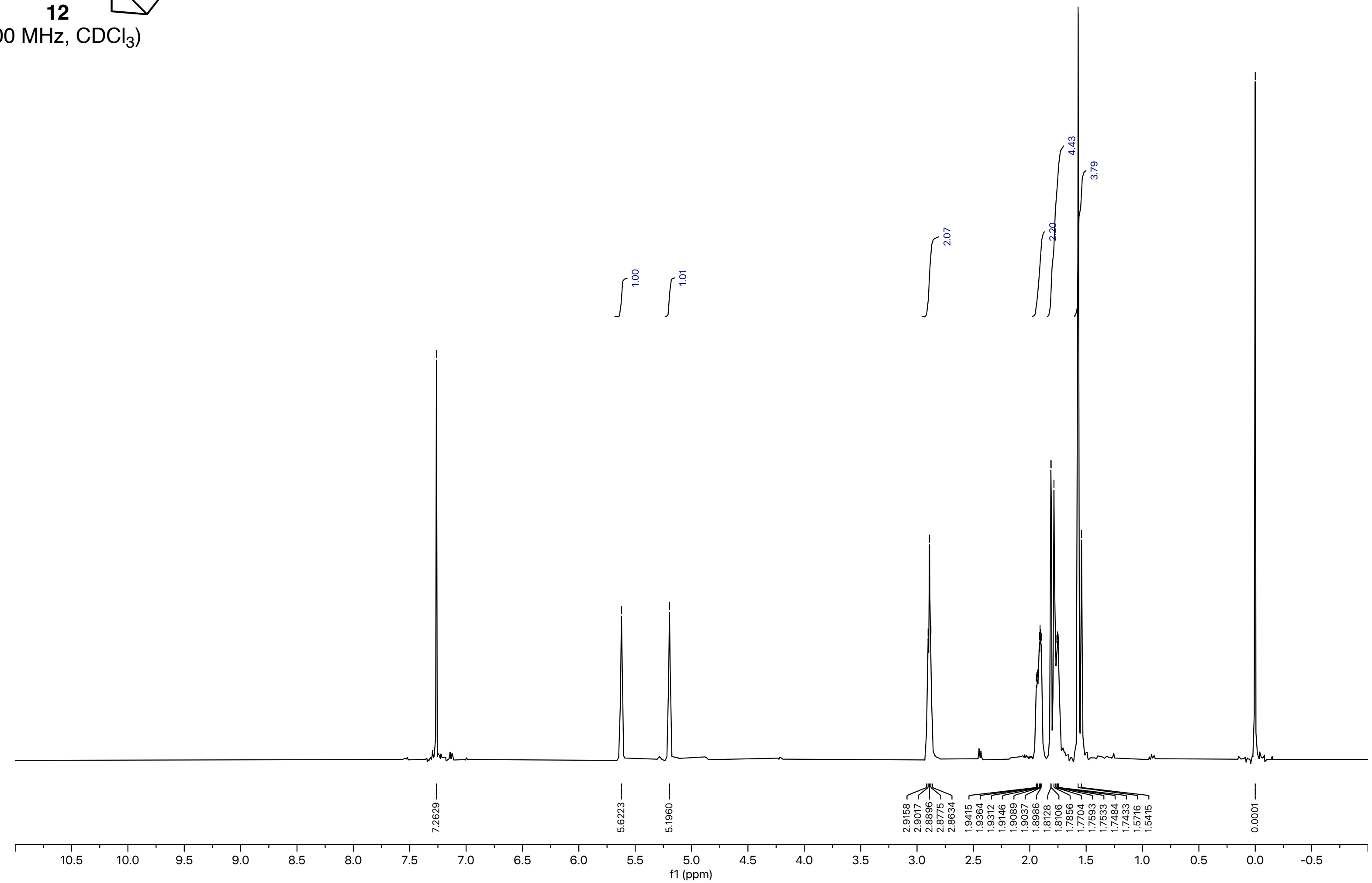

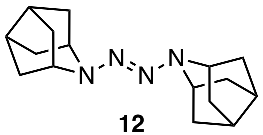

12
(100 MHz, $CDCl_3$)

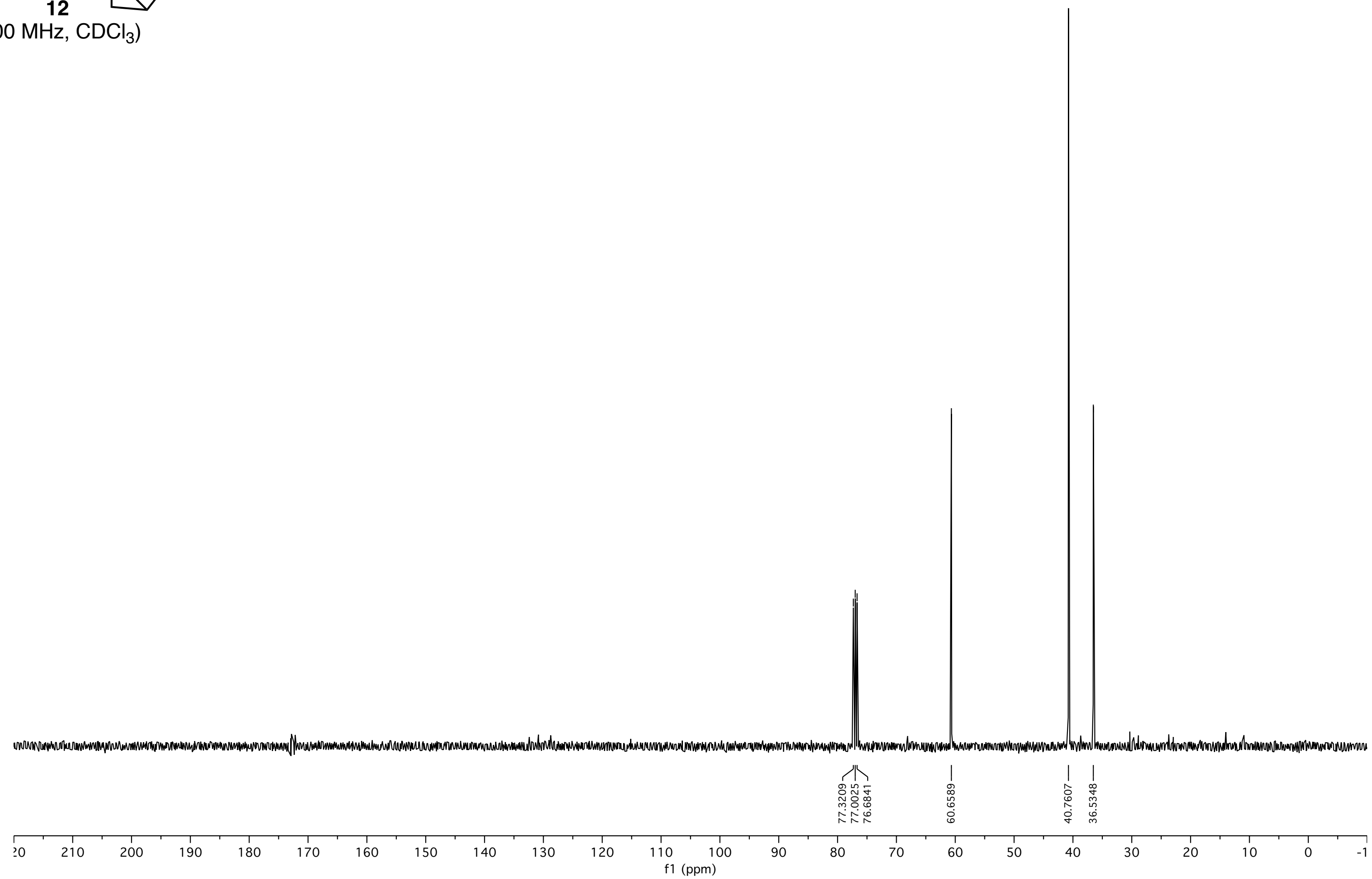

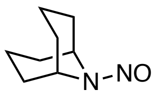

S8
(400 MHz, $CDCl_3$)

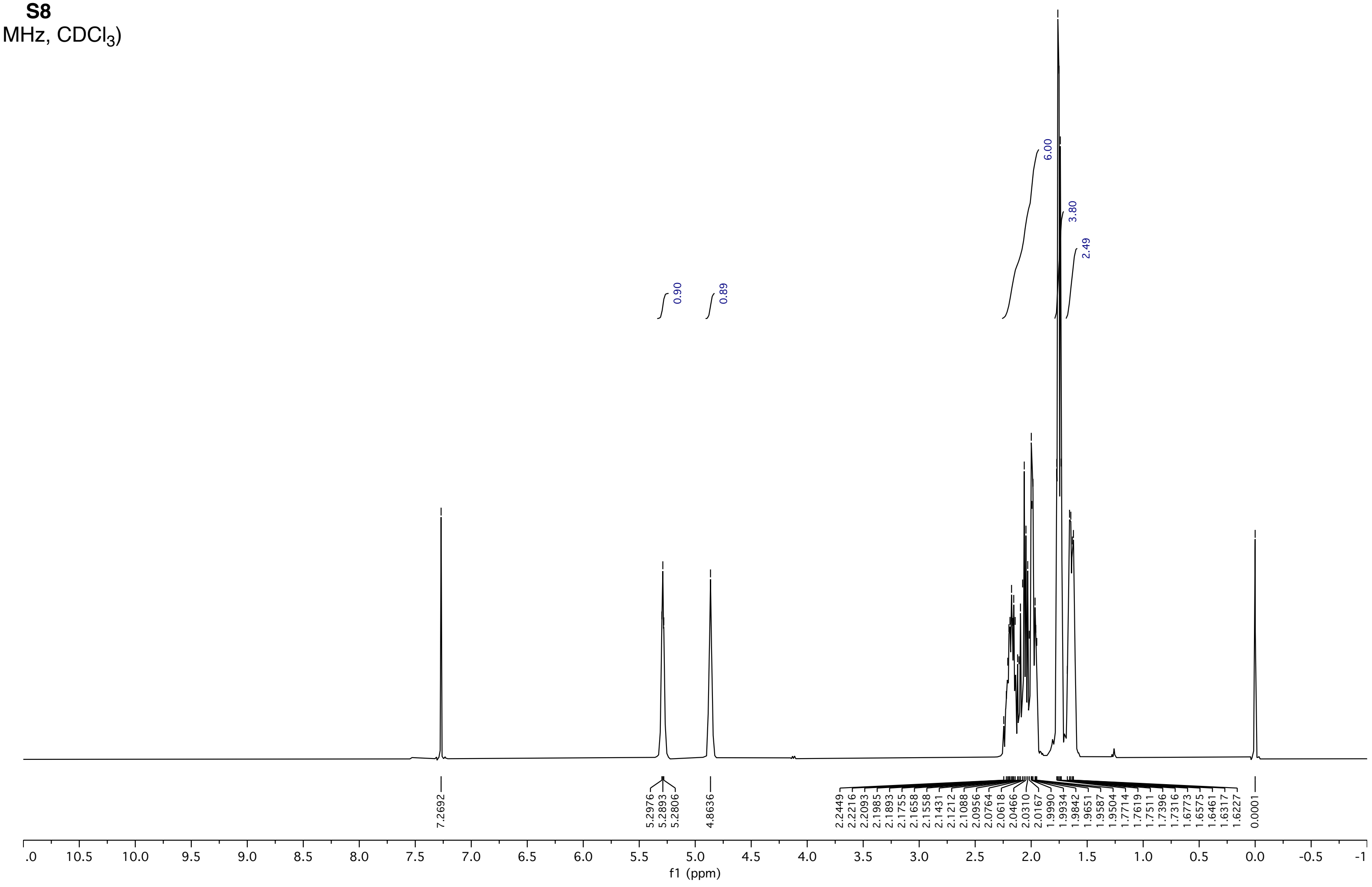

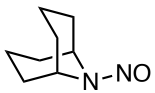

S8
(100 MHz, $CDCl_3$)

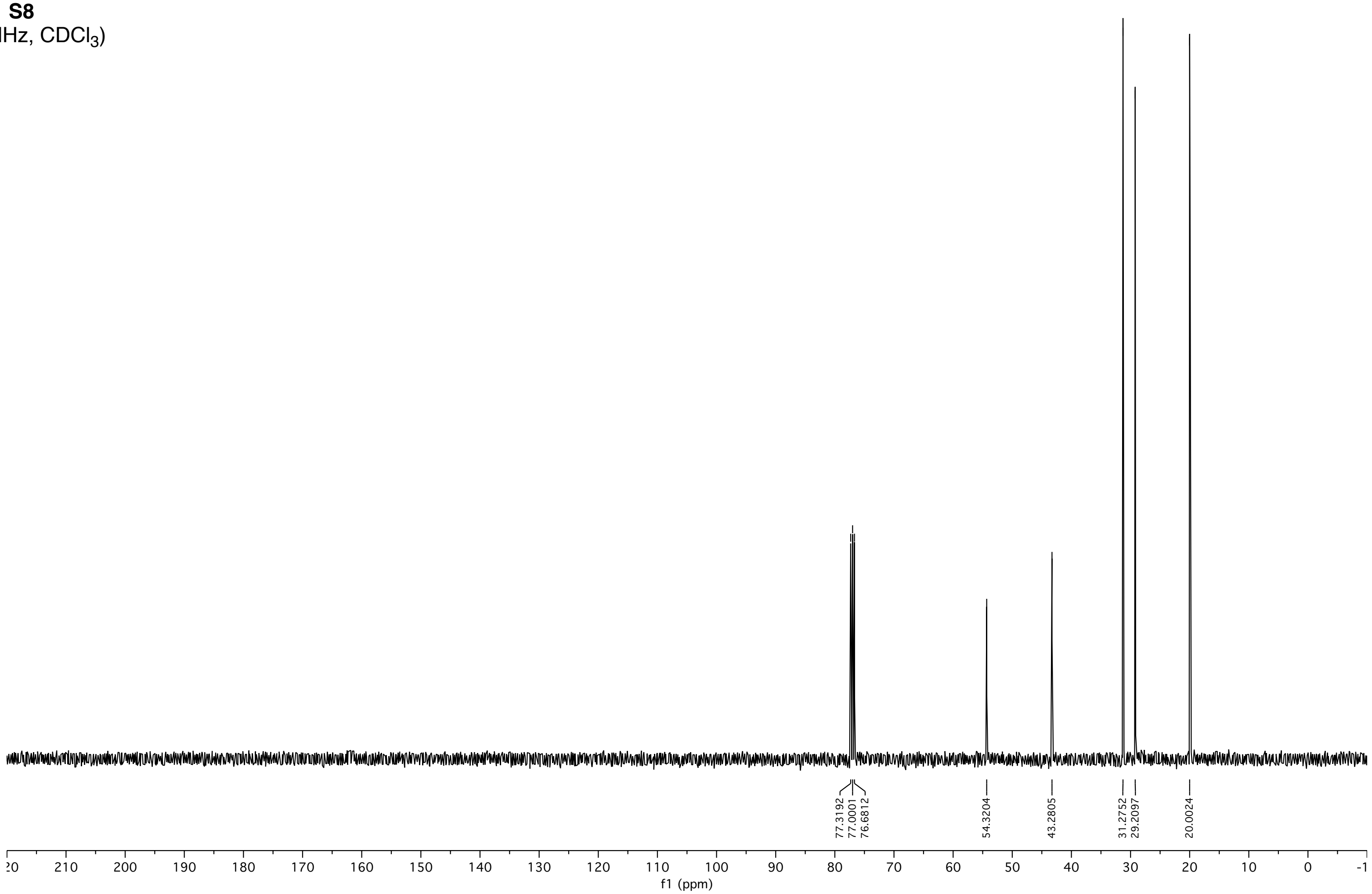

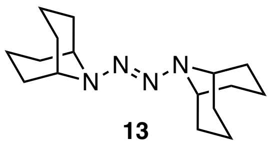

13
(400 MHz, $CDCl_3$)

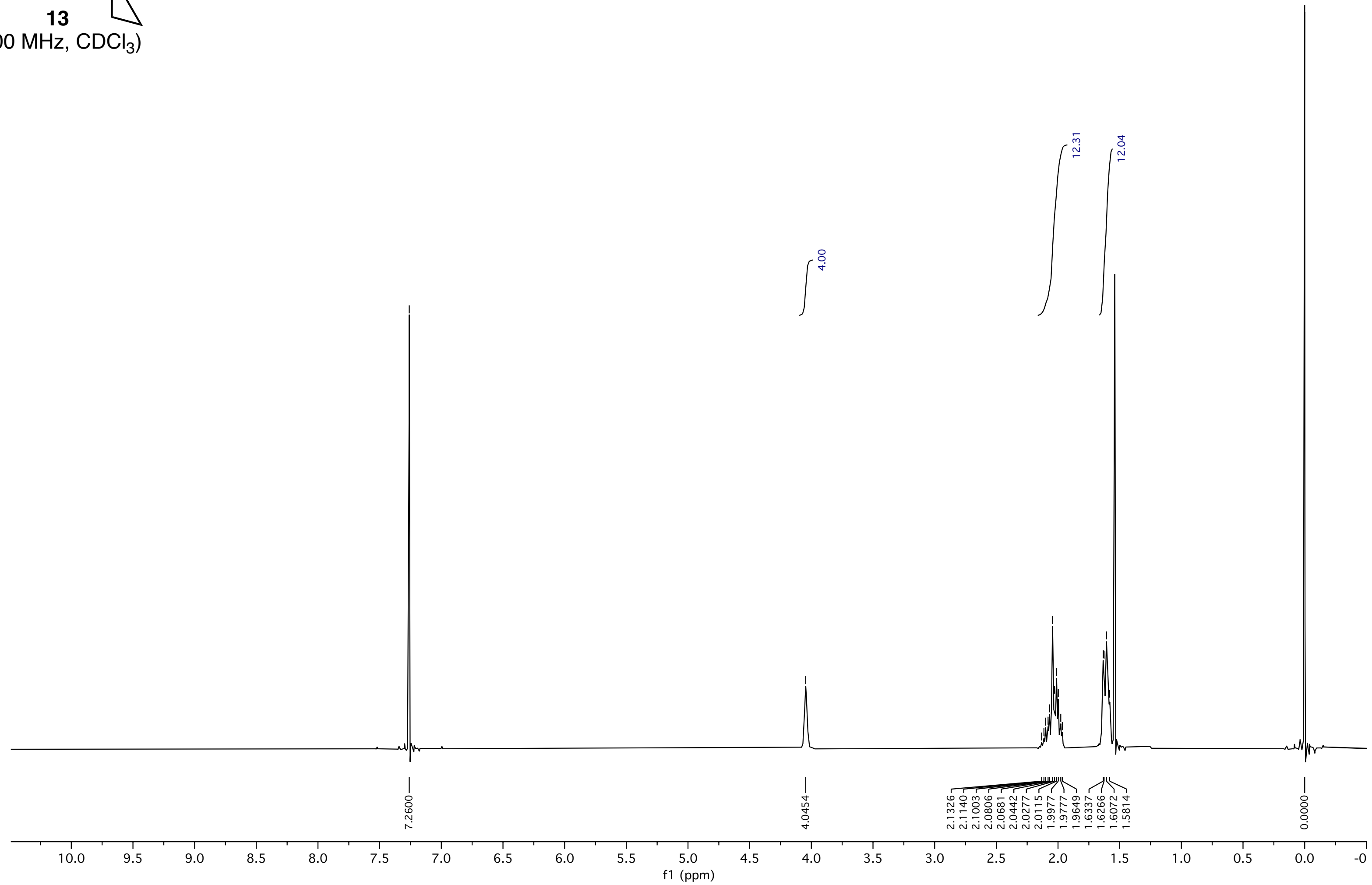

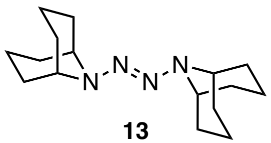

13
(100 MHz, $CDCl_3$)

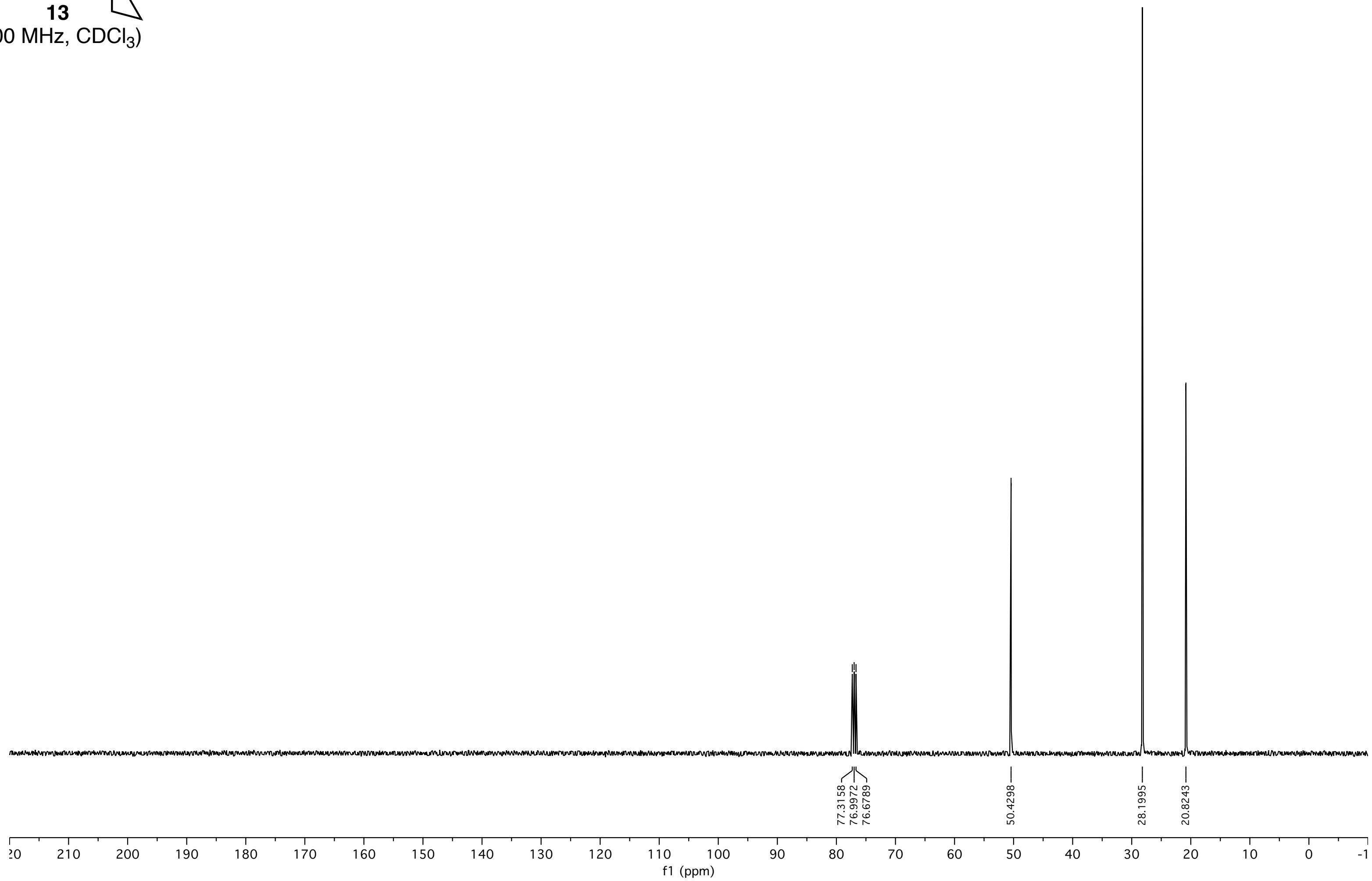

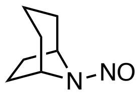

S13
(400 MHz, $CDCl_3$)

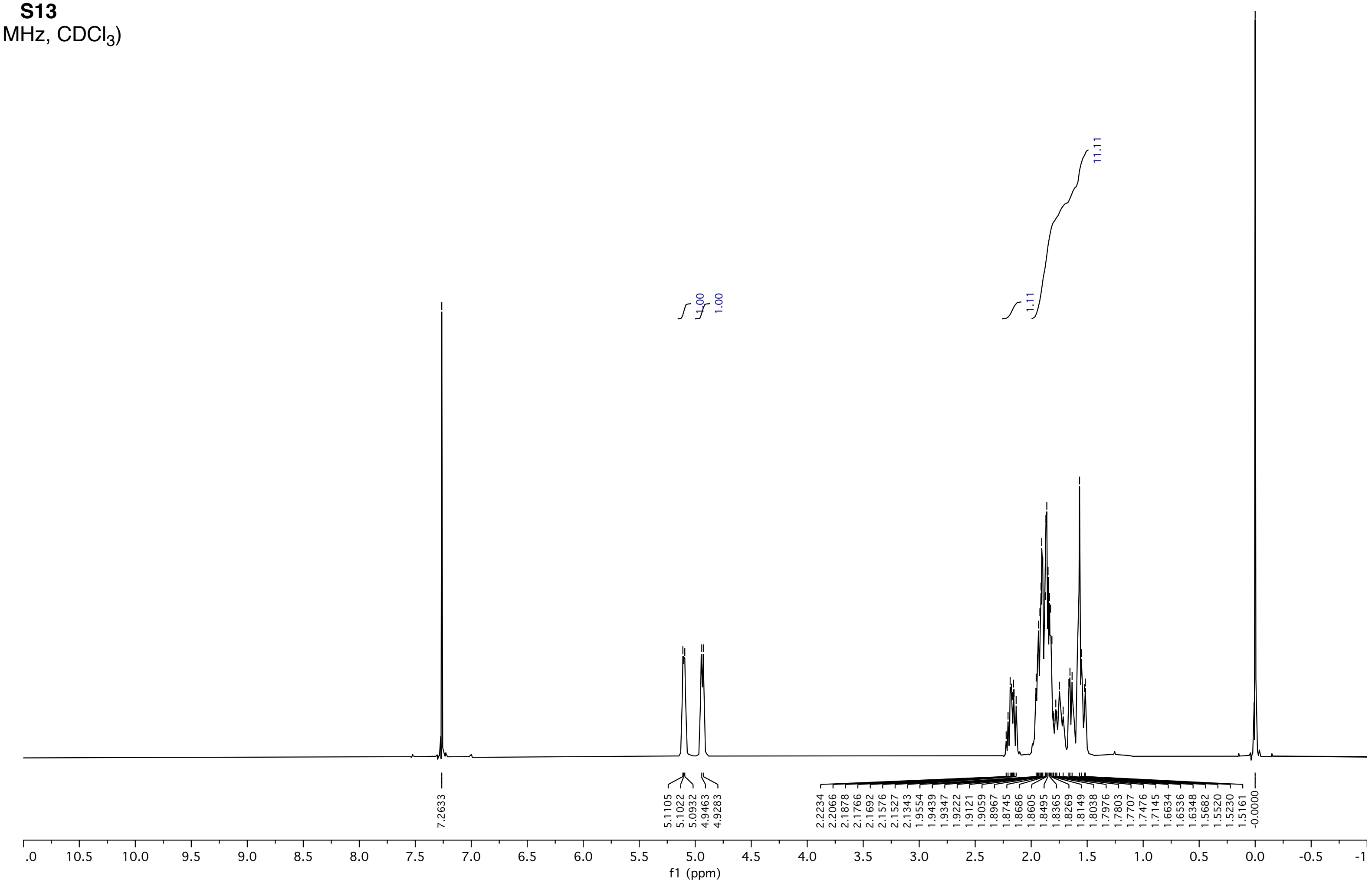

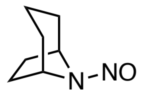

S13
(100 MHz, $CDCl_3$)

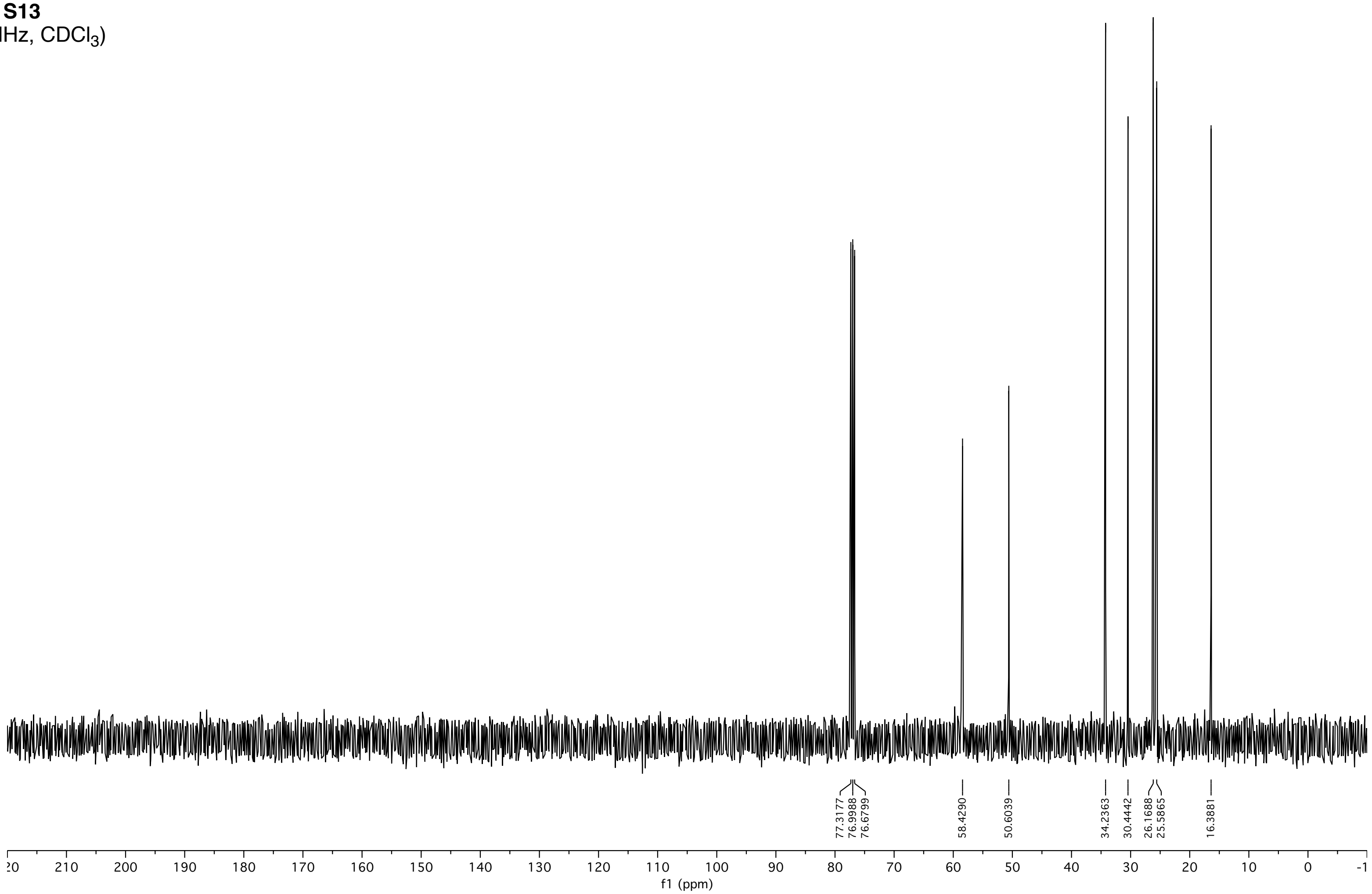

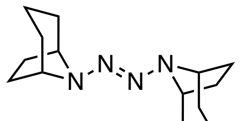

14
(400 MHz, $CDCl_3$)

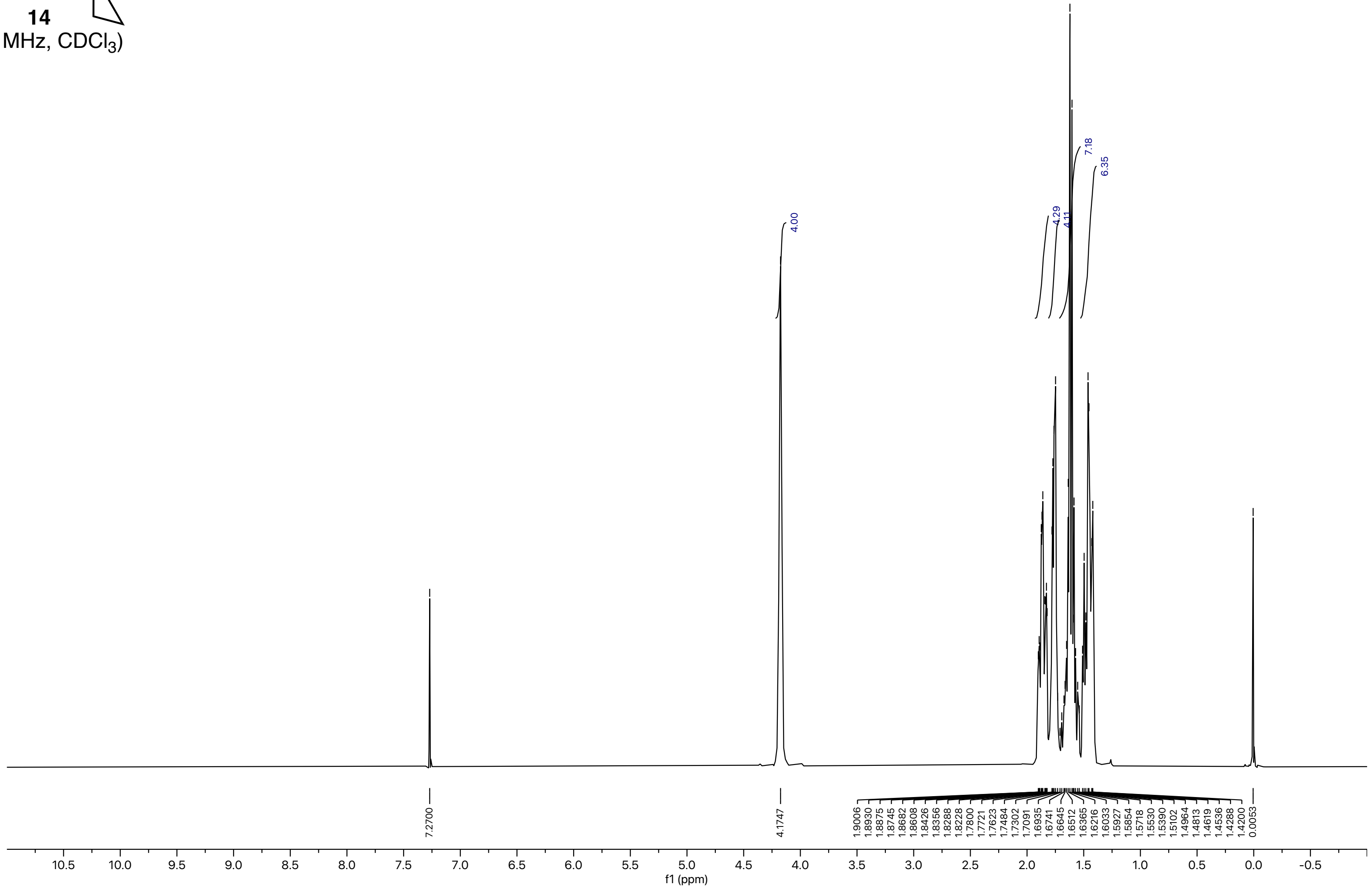

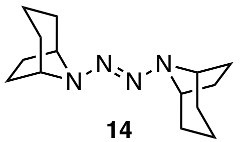

14
(100 MHz, $CDCl_3$)

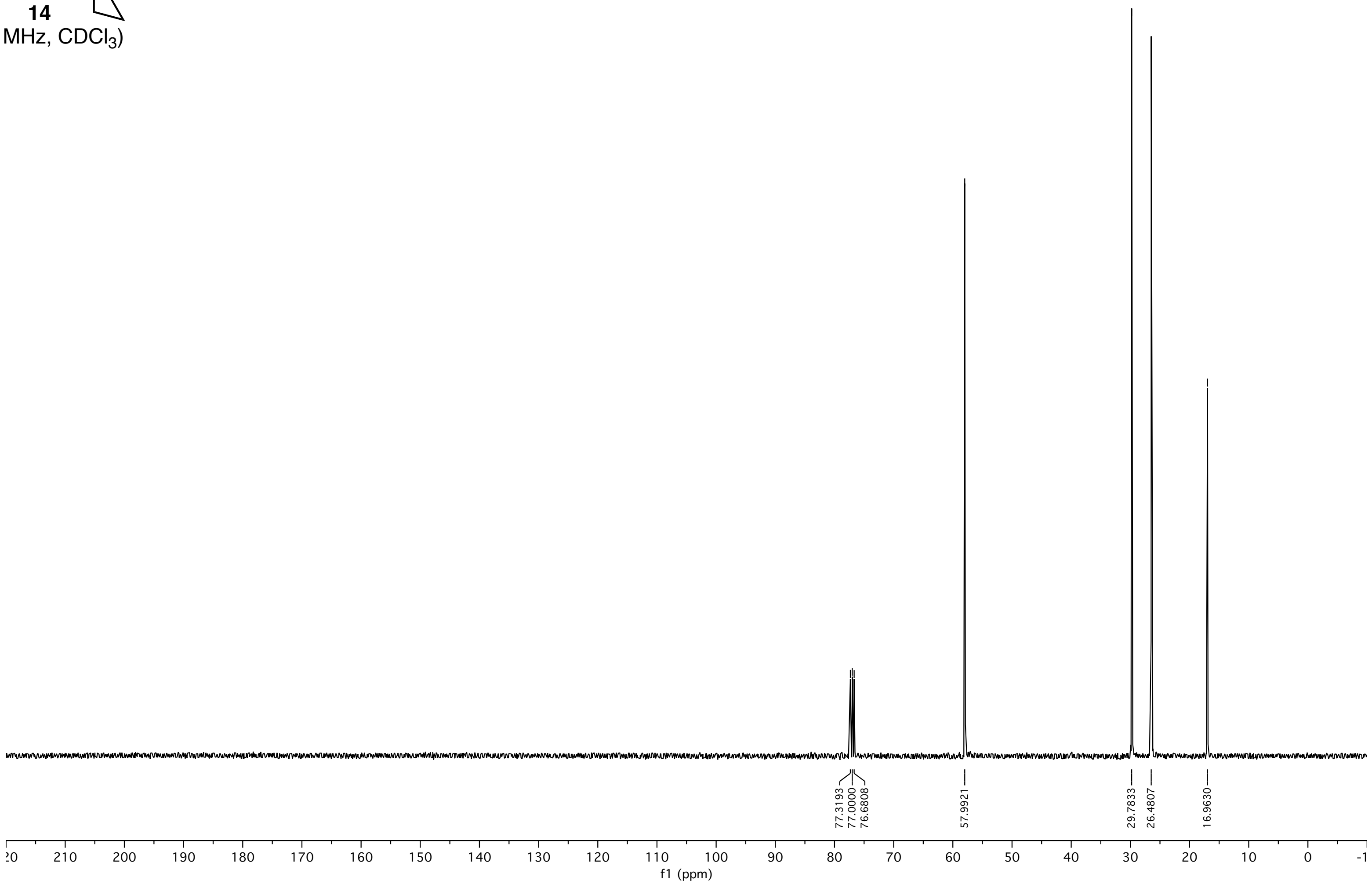

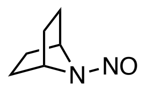

S18
(400 MHz, $CDCl_3$)

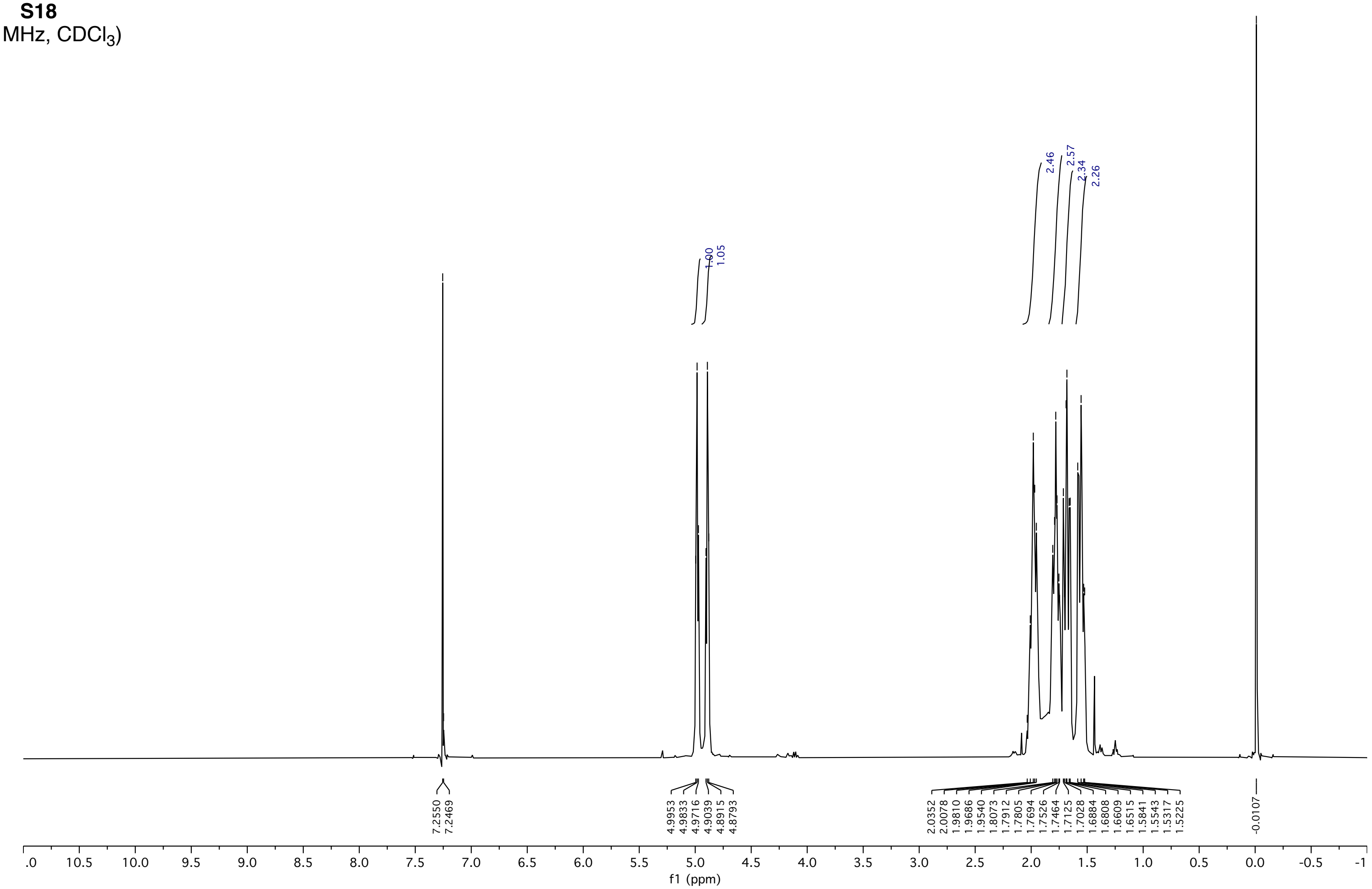

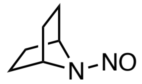

S18
(100 MHz, $CDCl_3$)

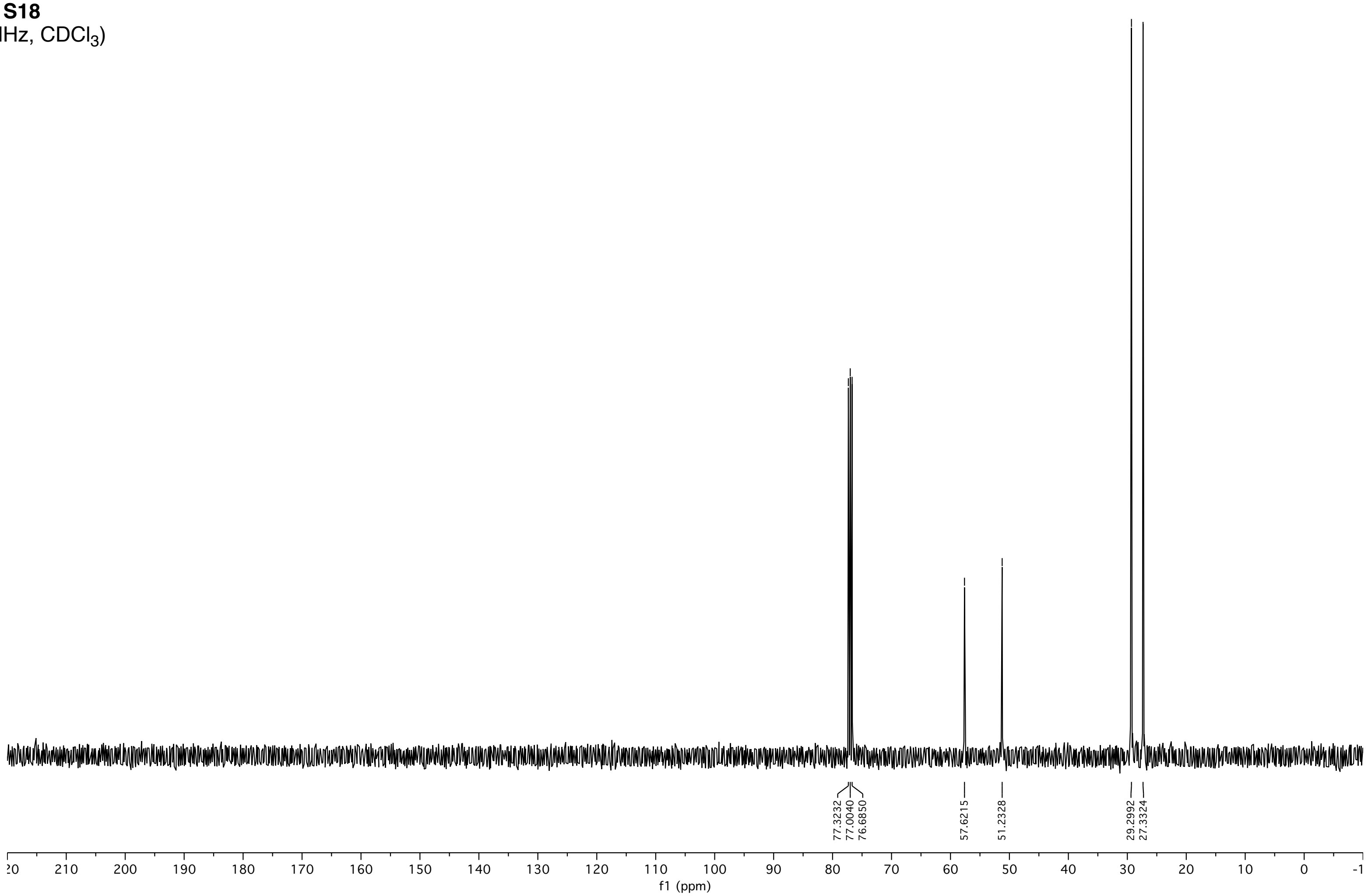

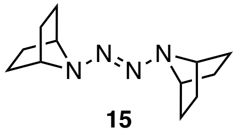

15
(400 MHz, $CDCl_3$)

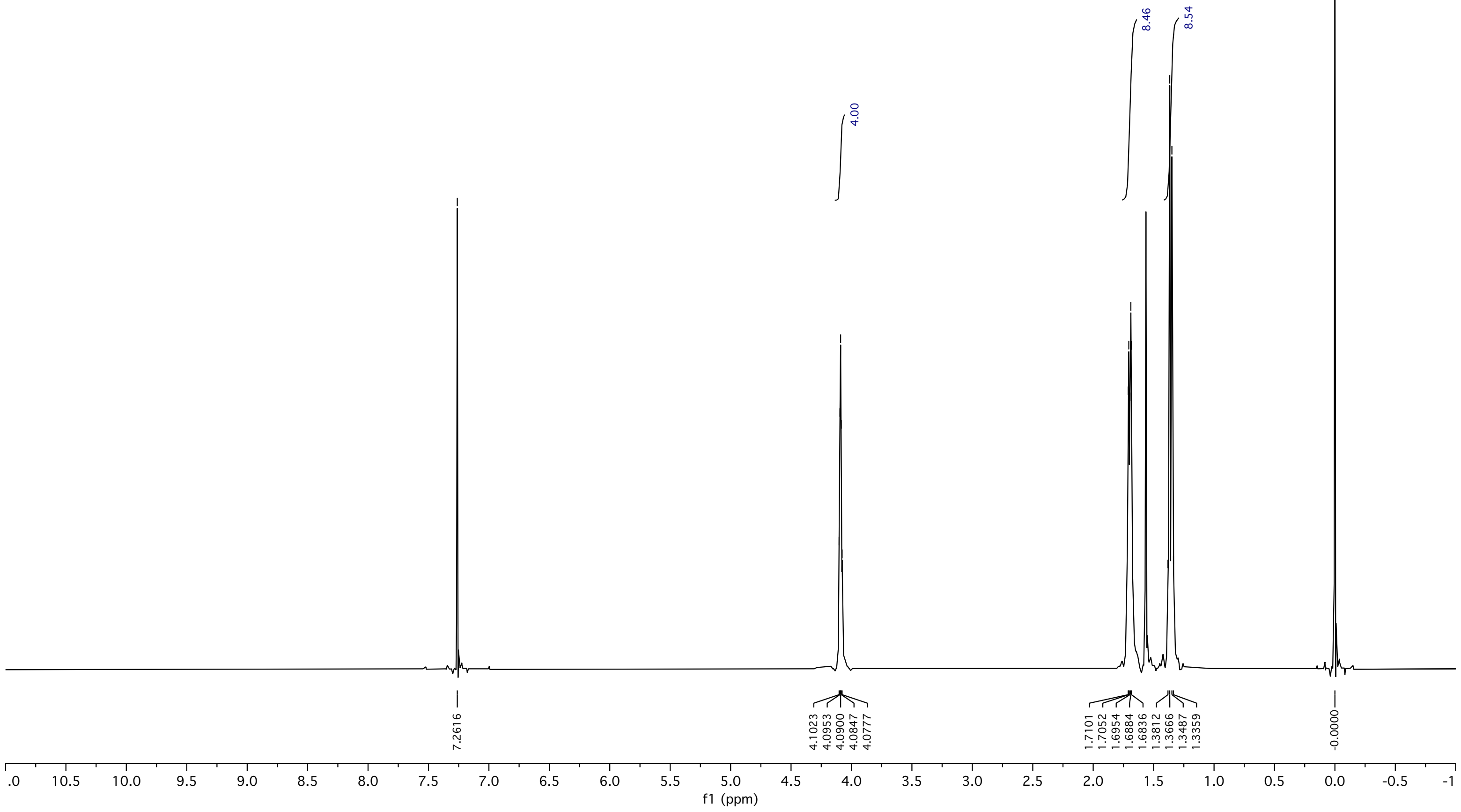

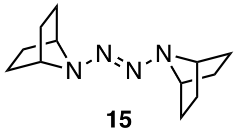

15
(100 MHz, $CDCl_3$)

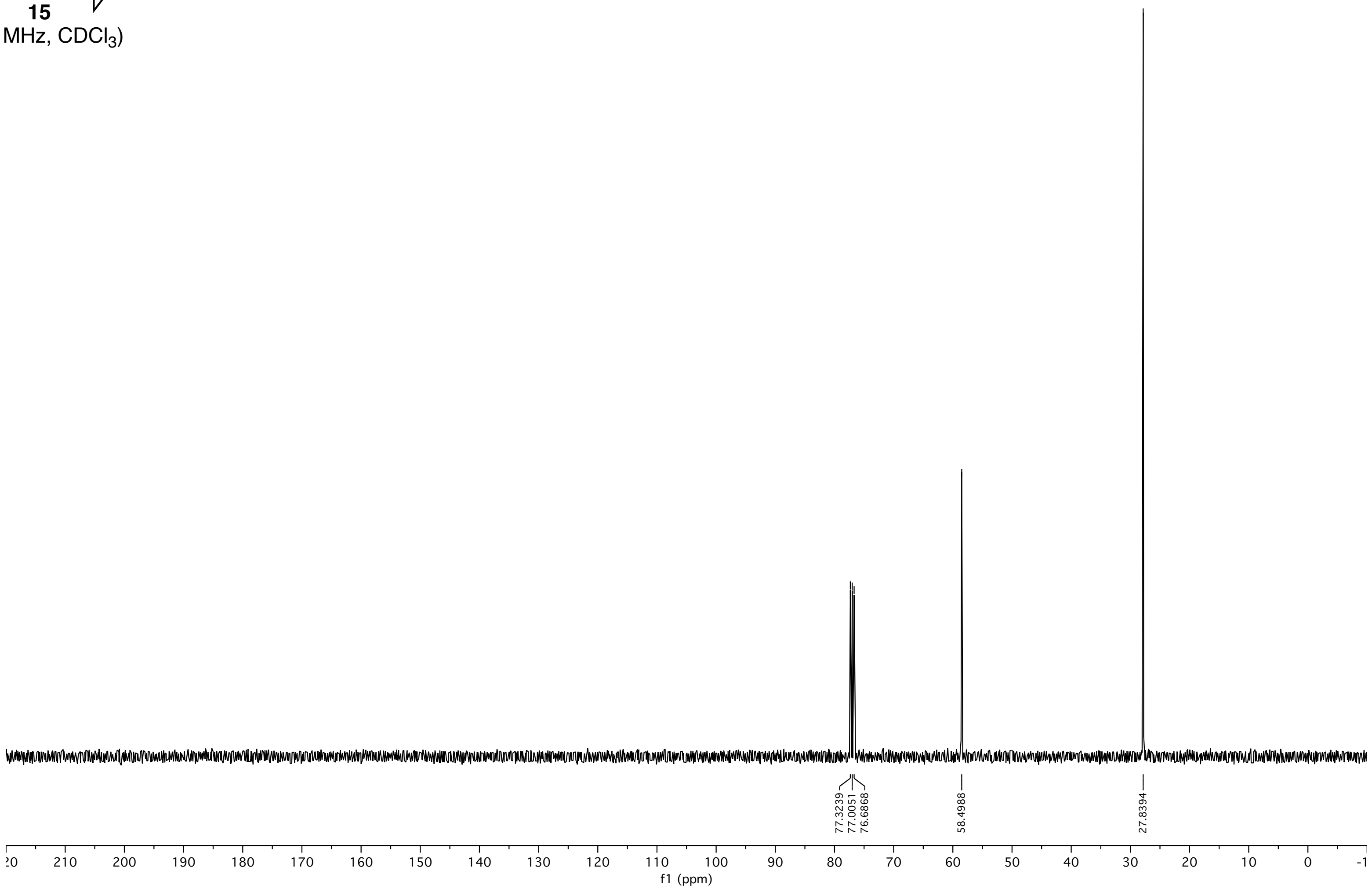

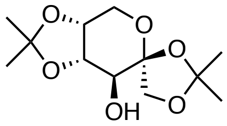

21e
(400 MHz, $CDCl_3$)

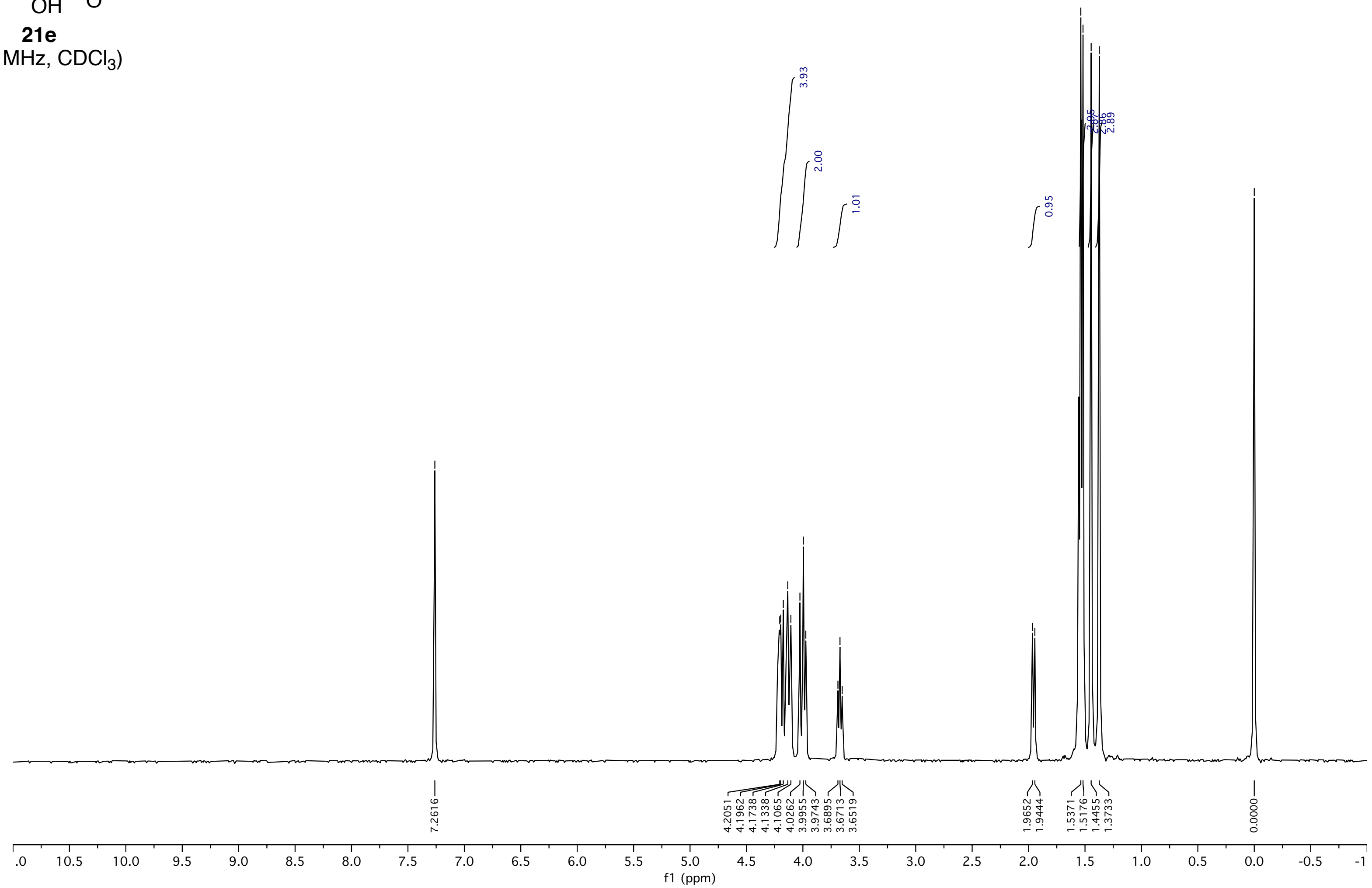

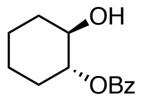

21f
(400 MHz, $CDCl_3$)

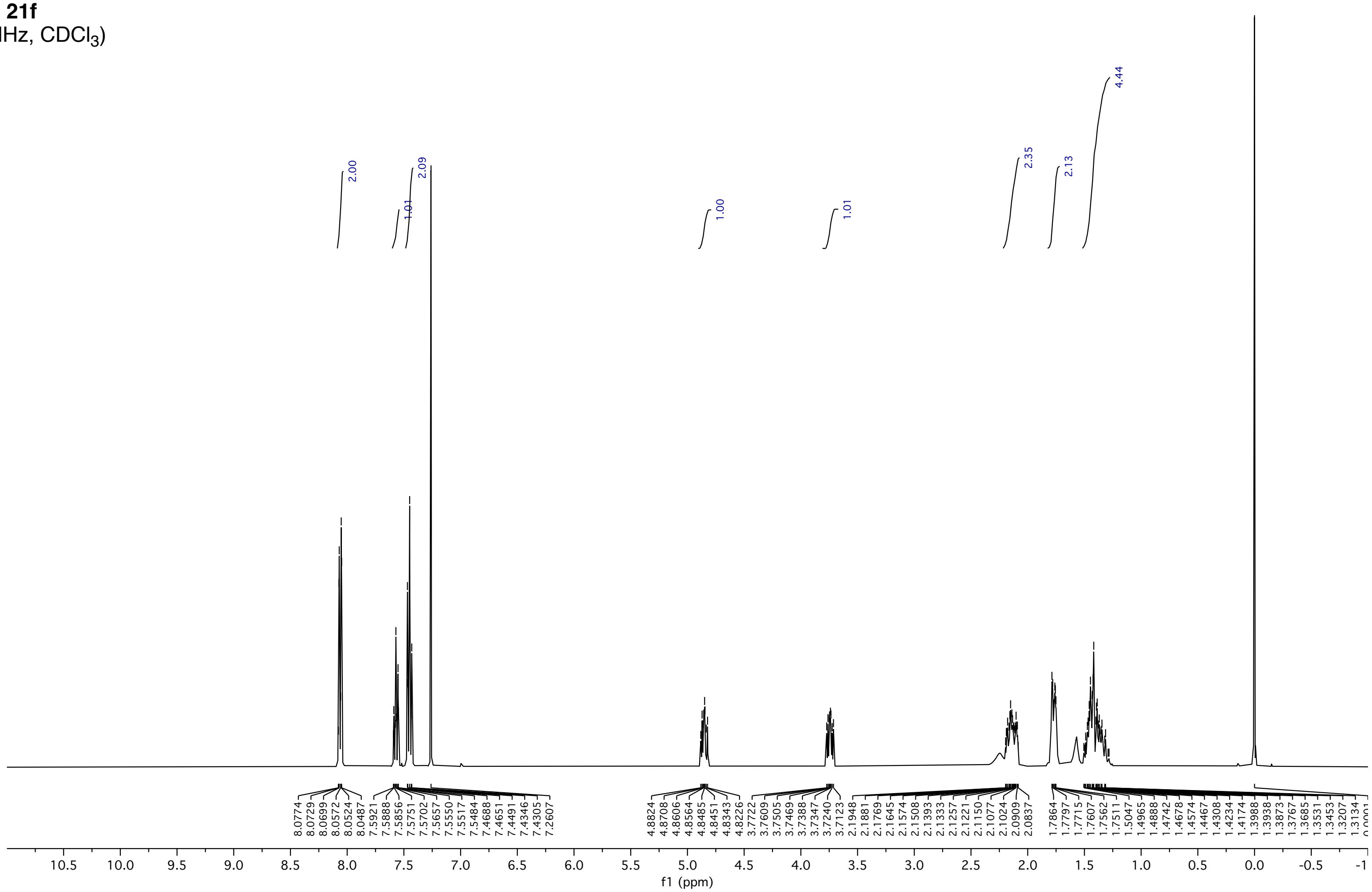

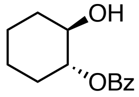

21f
(100 MHz, $CDCl_3$)

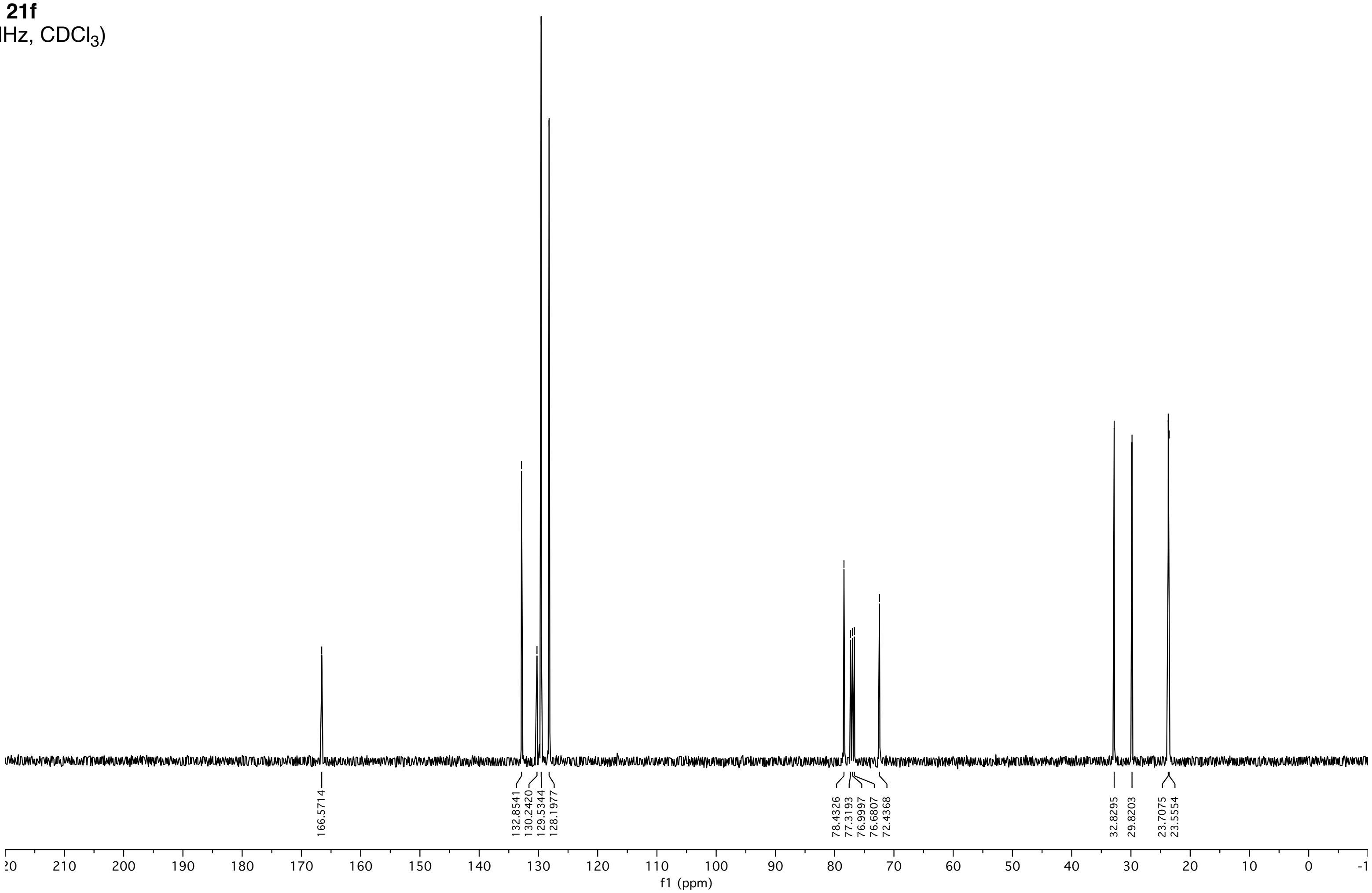

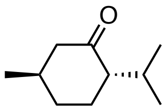

22a
(400 MHz, $CDCl_3$)

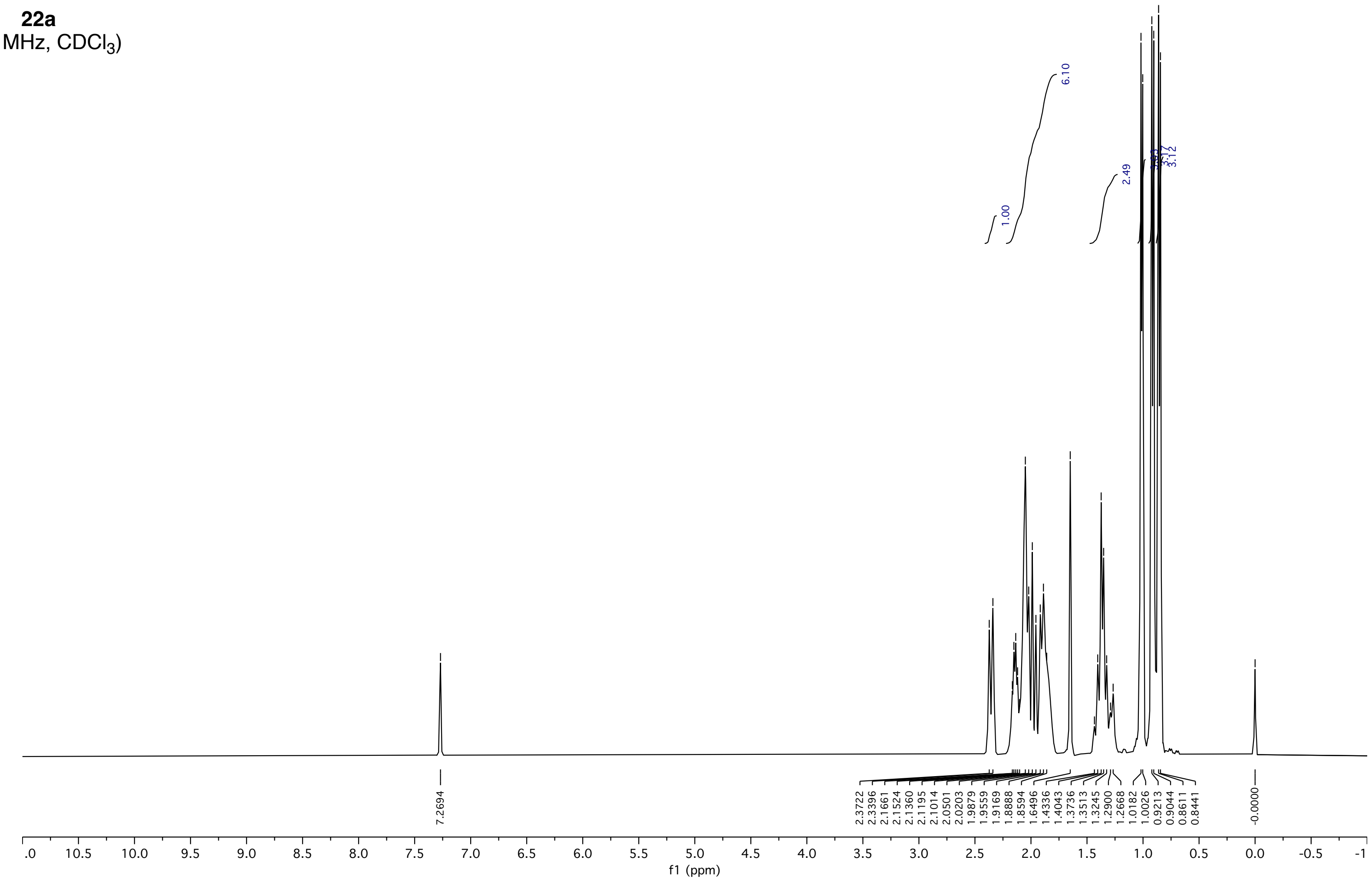

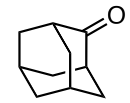

22b
(400 MHz, $CDCl_3$)

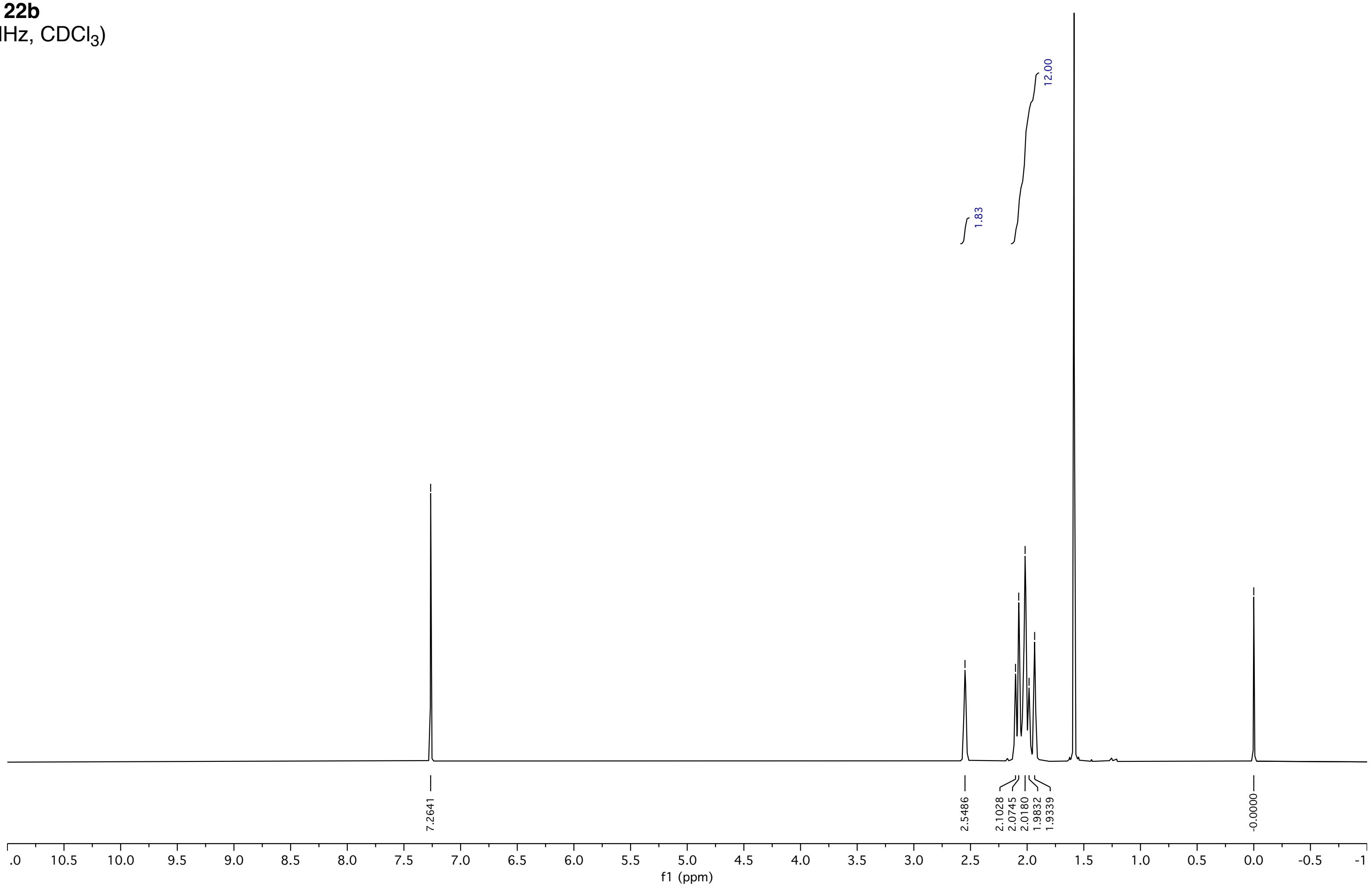

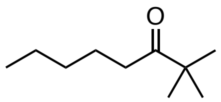

22c
(400 MHz, $CDCl_3$)

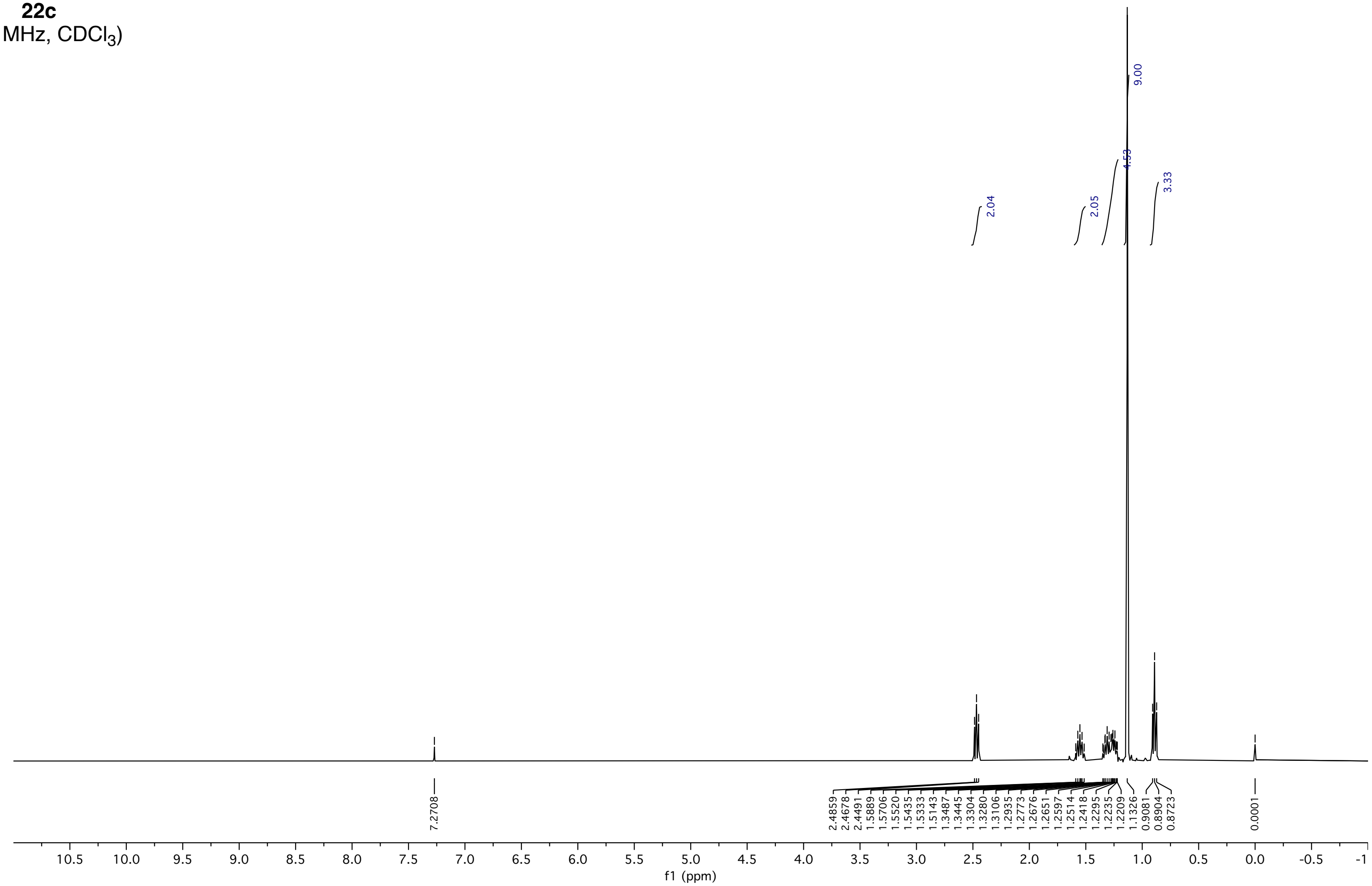

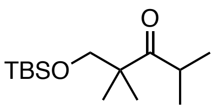

22d
(400 MHz, $CDCl_3$)

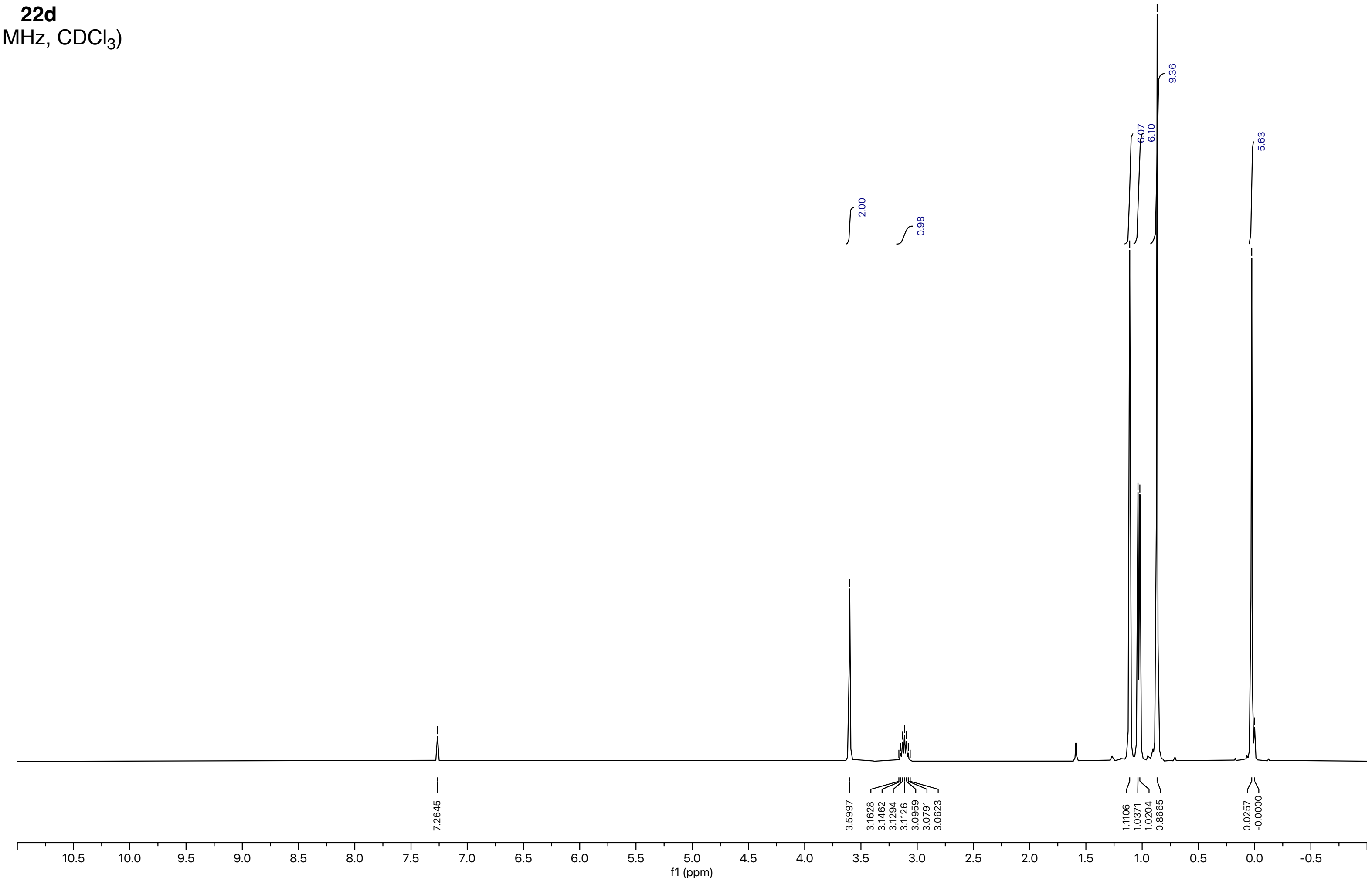

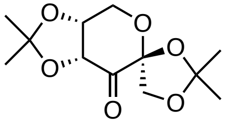

22e
(400 MHz, $CDCl_3$)

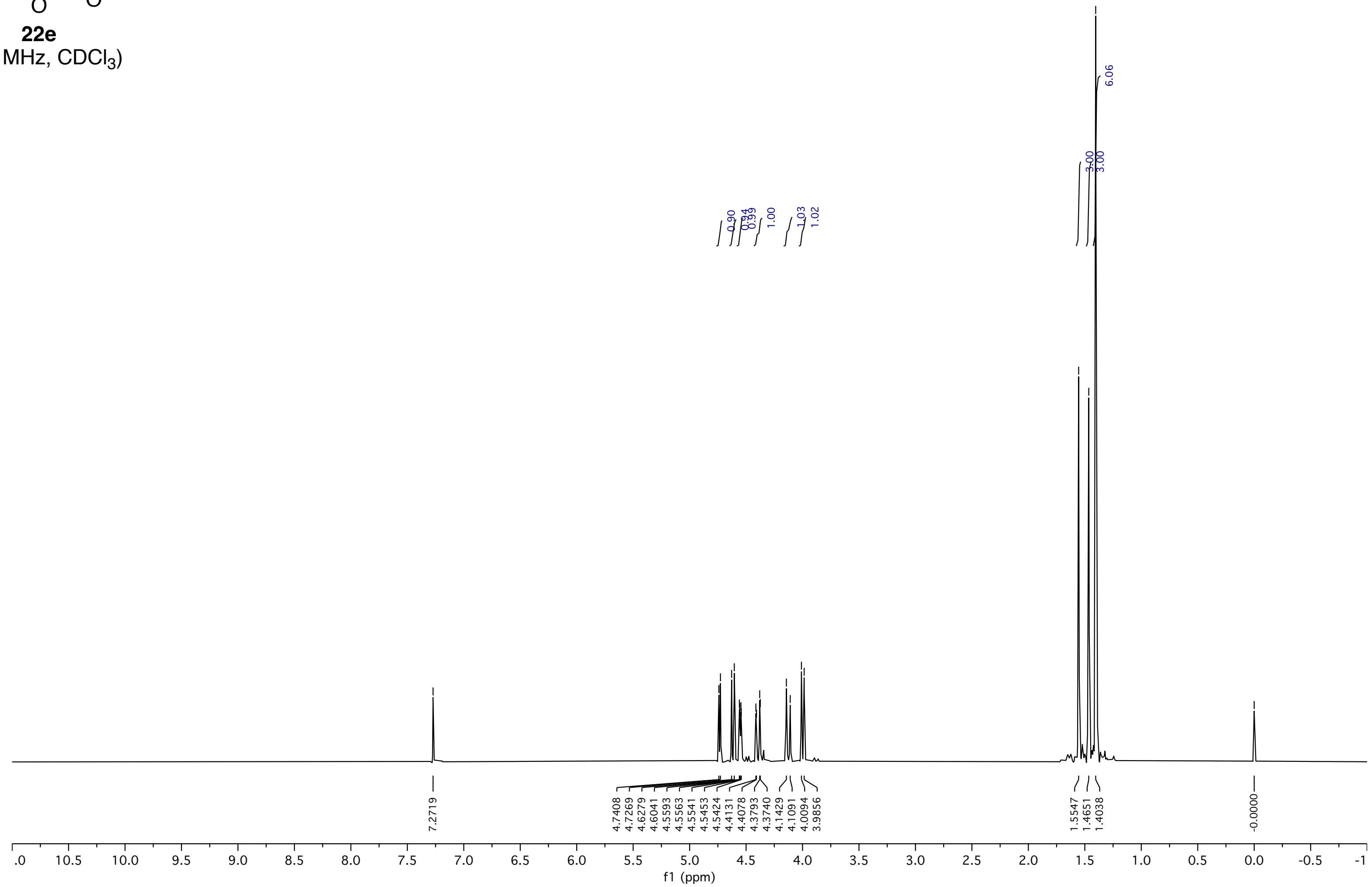

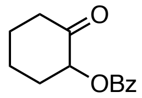

22f
(400 MHz, $CDCl_3$)

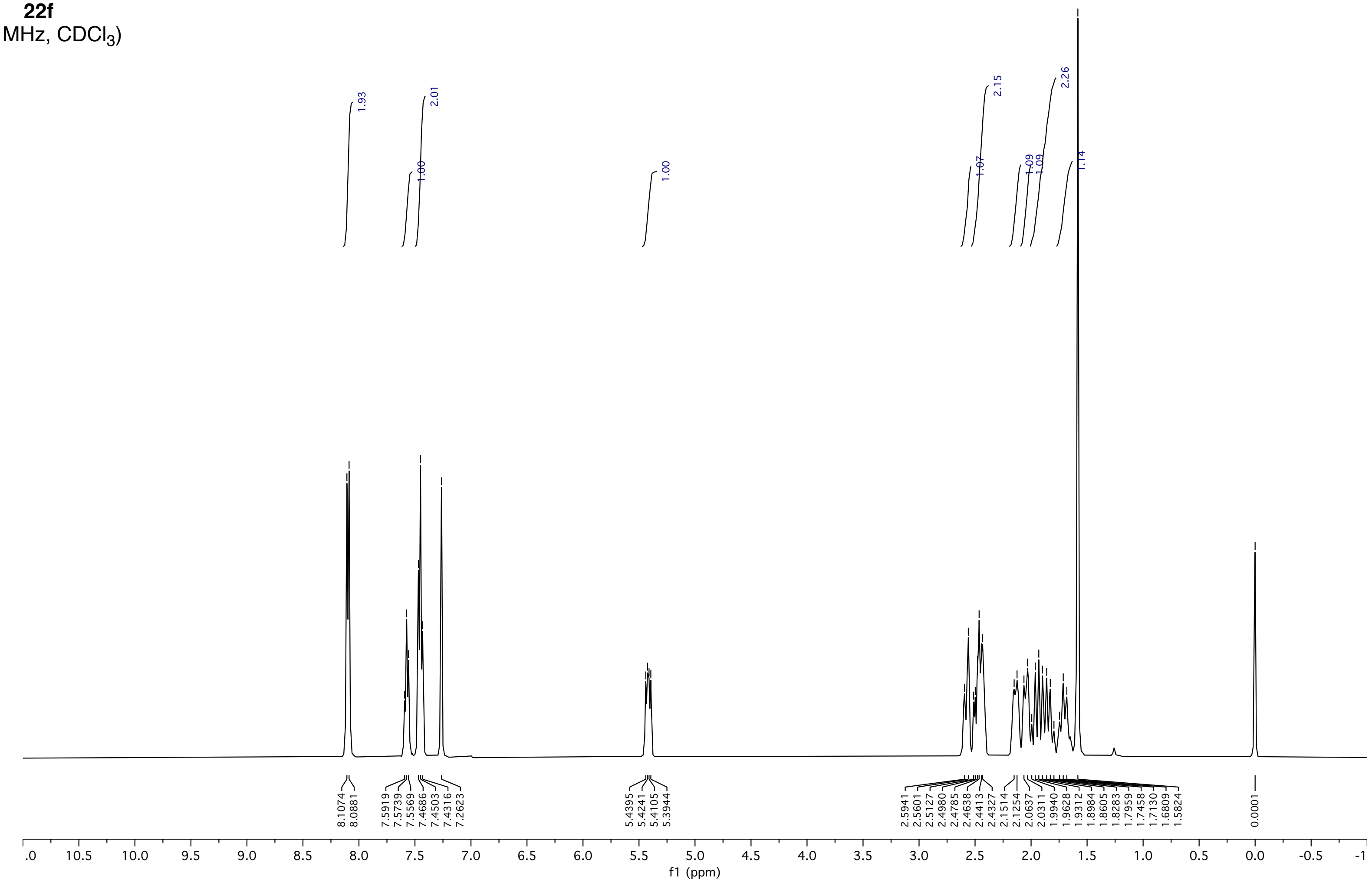

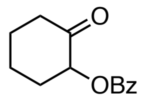

22f
(100 MHz, $CDCl_3$)

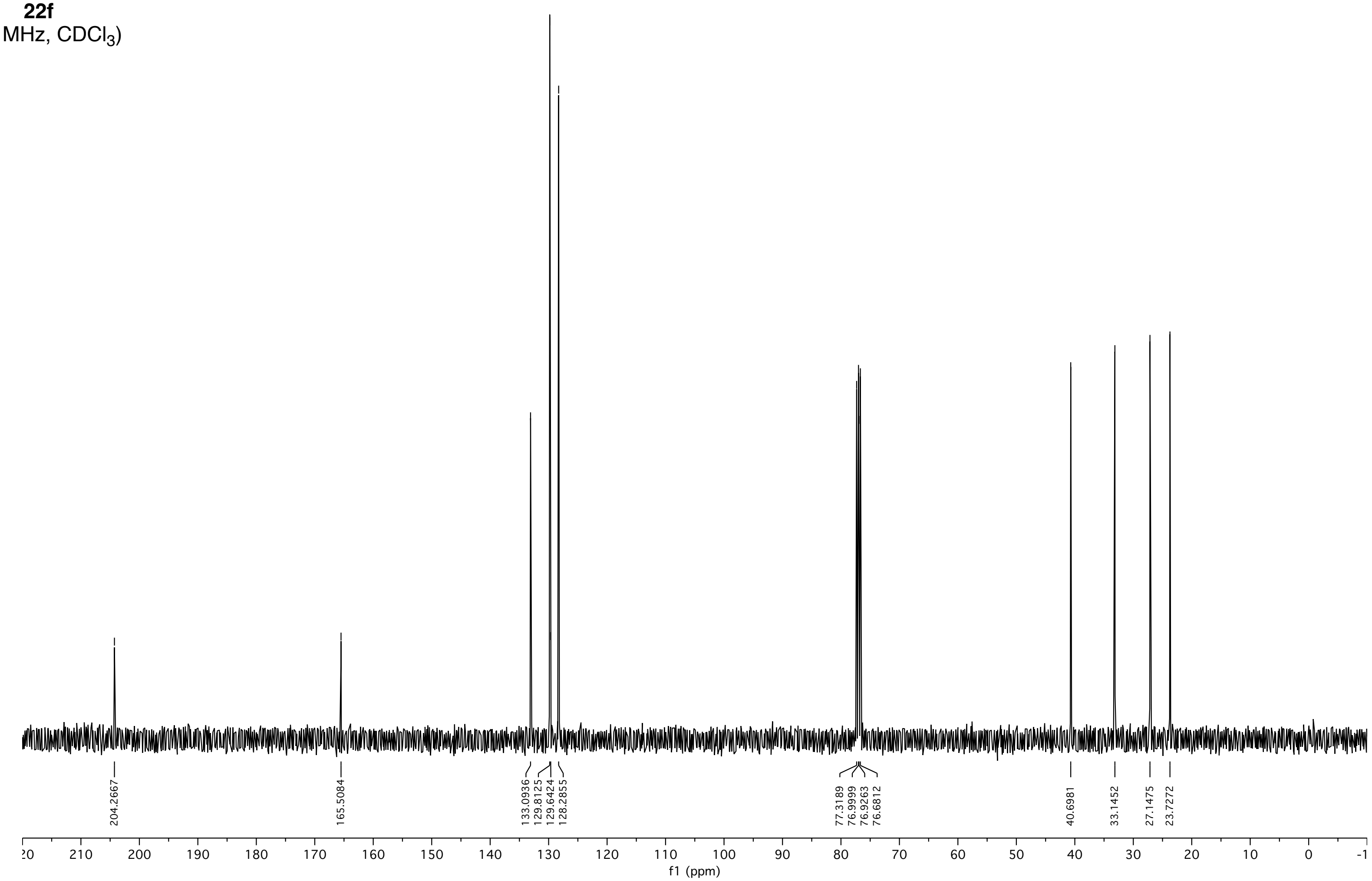

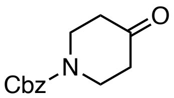

22g
(400 MHz, $CDCl_3$)

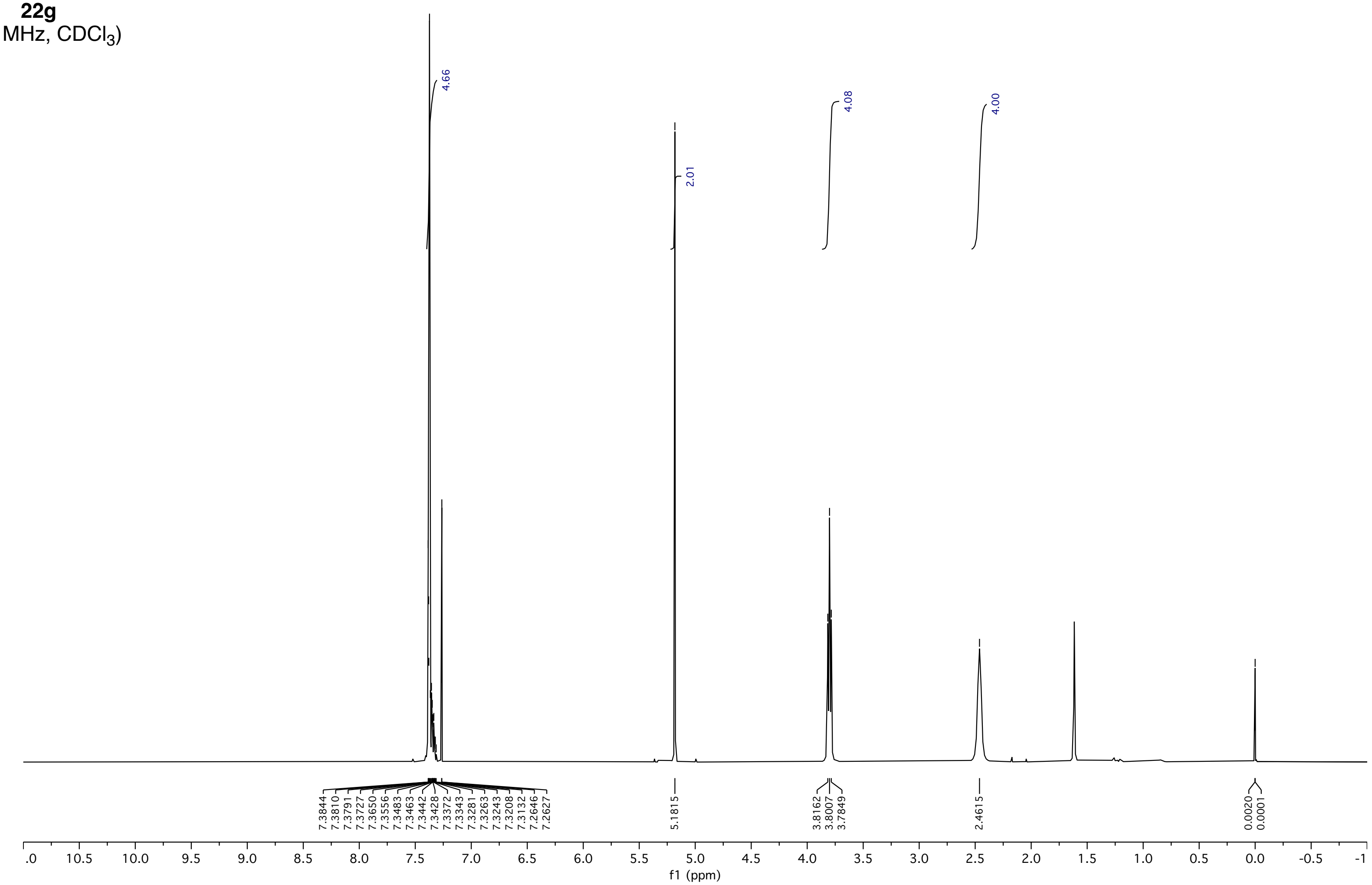

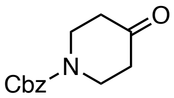

22g
(100 MHz, $CDCl_3$)

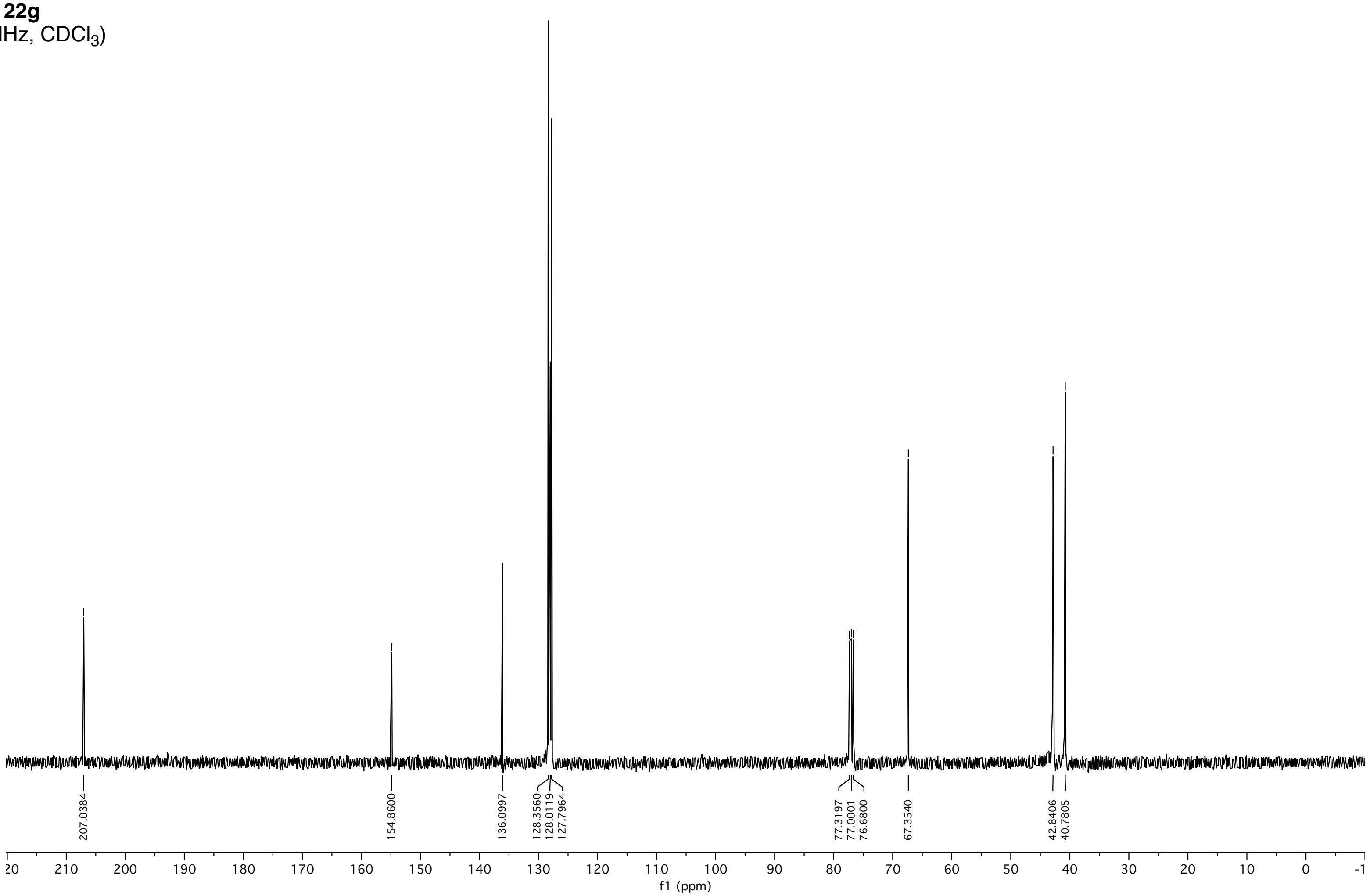

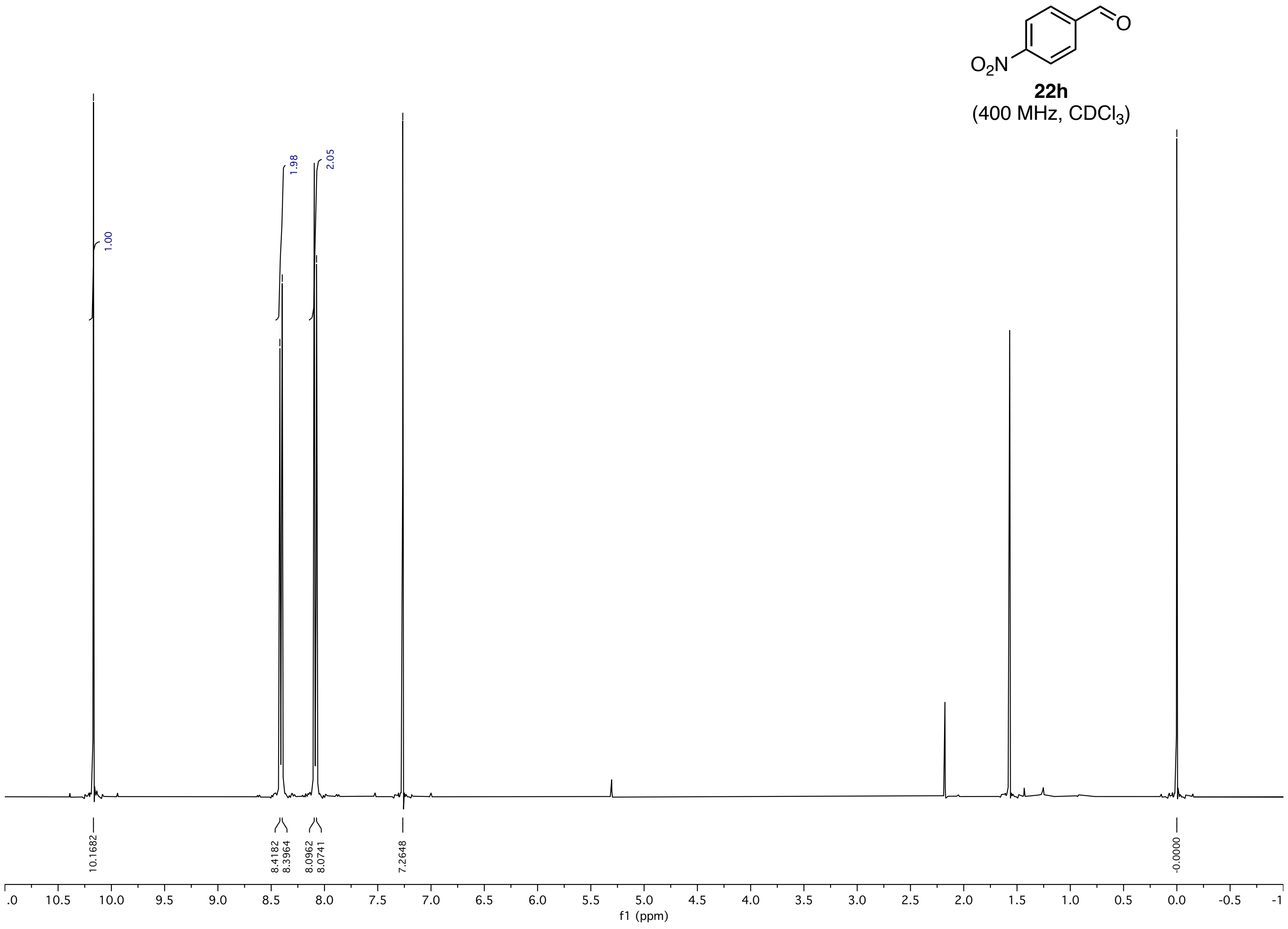

O2N
O
22h
(400 MHz, CDCl3)
1.00
1.98
2.05
10.1682
8.4182
8.3964
8.0962
8.0741
7.2648
-0.0000
.0
10.5
10.0
9.5
9.0
8.5
8.0
7.5
7.0
6.5
6.0
5.5
5.0
4.5
4.0
3.5
3.0
2.5
2.0
1.5
1.0
0.5
0.0
-0.5
-1
f1 (ppm)

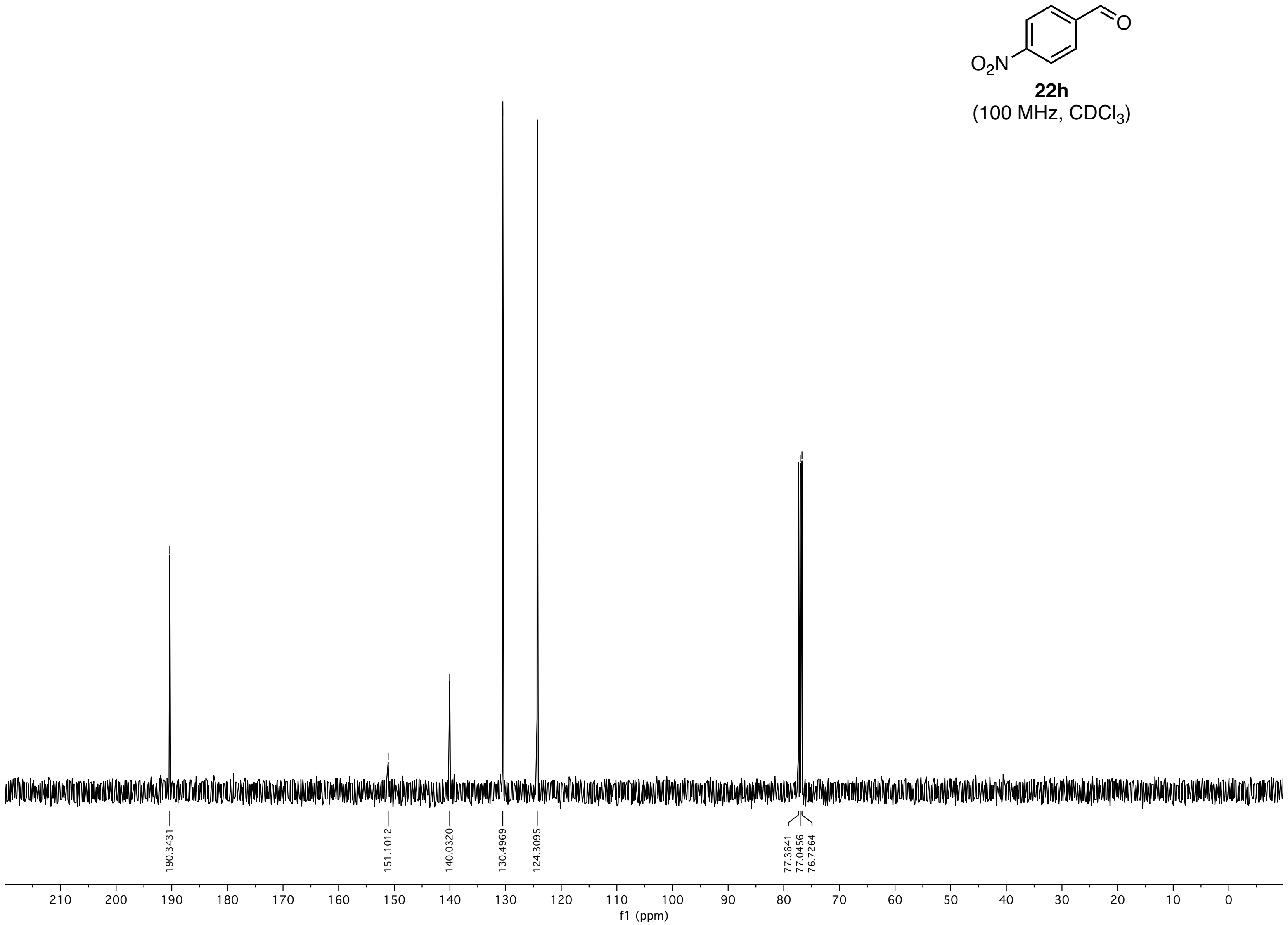

O_2N
O
22h
(100 MHz, $CDCl_3$)
190.3431
151.1012
140.0320
130.4969
124.3095
77.3641
77.0456
76.7264
210
200
190
180
170
160
150
140
130
120
110
100
90
80
70
60
50
40
30
20
10
0
f1 (ppm)

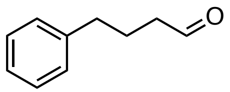

22i
(400 MHz, $CDCl_3$)

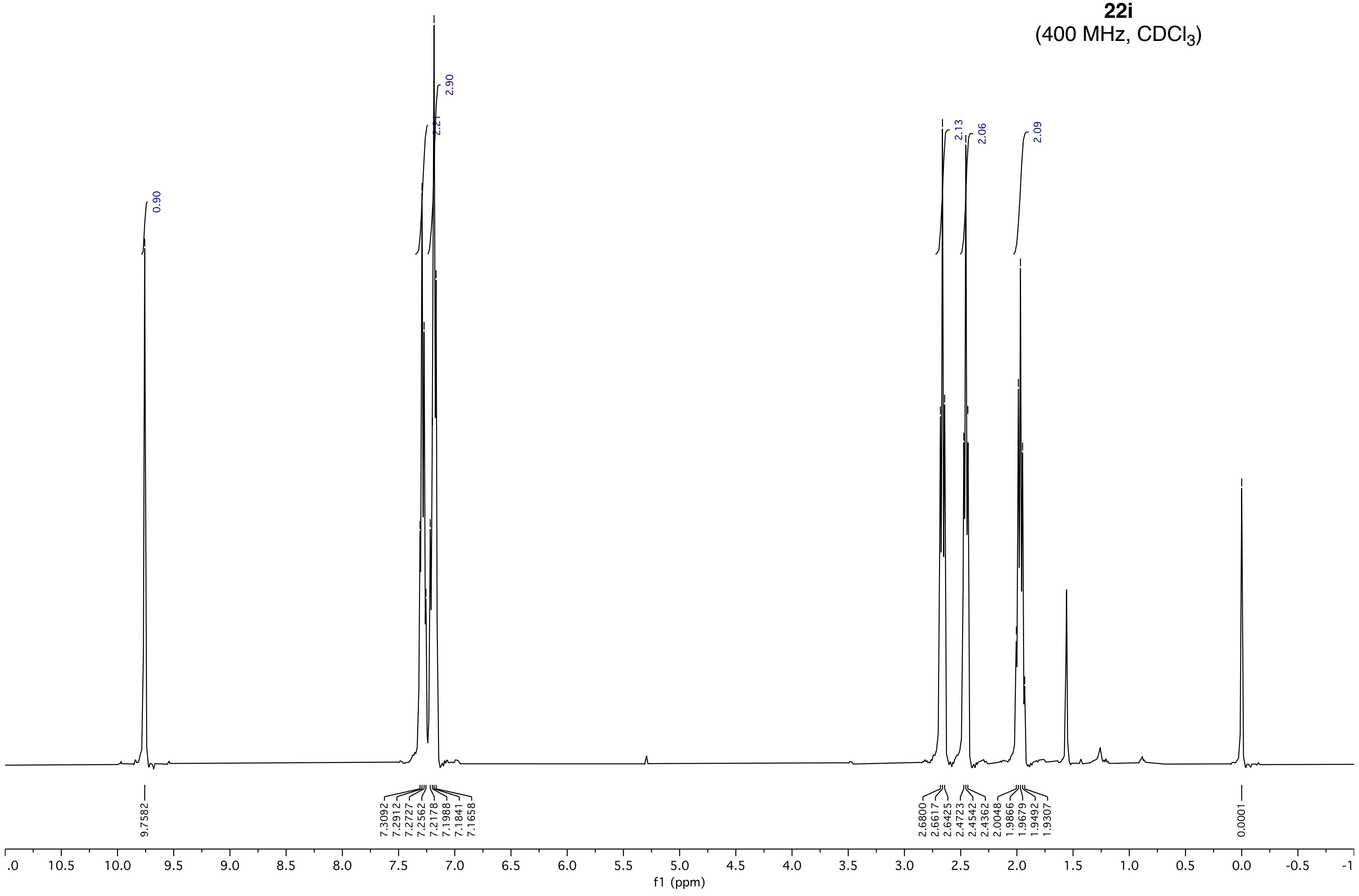

Supplement: Supplementary file 1 [file ja5c15272_si_001.pdf]
